# The Optimal Machine Learning-Based Missing Data Imputation for the Cox Proportional Hazard Model

**DOI:** 10.3389/fpubh.2021.680054

**Published:** 2021-07-05

**Authors:** Chao-Yu Guo, Ying-Chen Yang, Yi-Hau Chen

**Affiliations:** ^1^Institute of Public Health, School of Medicine, National Yang-Ming University, Taipei, Taiwan; ^2^Institute of Public Health, School of Medicine, National Yang Ming Chiao Tung University, Hsinchu, Taiwan; ^3^Institute of Statistical Science, Academia Sinica, Taipei, Taiwan

**Keywords:** machine learning, k-nearest neighbors imputation, random forest imputation, survival data simulation, cox proportional hazard model

## Abstract

An adequate imputation of missing data would significantly preserve the statistical power and avoid erroneous conclusions. In the era of big data, machine learning is a great tool to infer the missing values. The root means square error (RMSE) and the proportion of falsely classified entries (PFC) are two standard statistics to evaluate imputation accuracy. However, the Cox proportional hazards model using various types requires deliberate study, and the validity under different missing mechanisms is unknown. In this research, we propose supervised and unsupervised imputations and examine four machine learning-based imputation strategies. We conducted a simulation study under various scenarios with several parameters, such as sample size, missing rate, and different missing mechanisms. The results revealed the type-I errors according to different imputation techniques in the survival data. The simulation results show that the non-parametric “missForest” based on the unsupervised imputation is the only robust method without inflated type-I errors under all missing mechanisms. In contrast, other methods are not valid to test when the missing pattern is informative. Statistical analysis, which is improperly conducted, with missing data may lead to erroneous conclusions. This research provides a clear guideline for a valid survival analysis using the Cox proportional hazard model with machine learning-based imputations.

## Background

Before statistical analysis, data management plays a crucial role and missing data occur frequently. If there are too many missing values, excluding the missing data from the analysis is not ideal since the loss of information is substantial. In addition to the reduced power, missing data may introduce potential biases or an unsolvable issue in statistical modeling. There are three significant missingness mechanisms (1). They are missing completely at random (MCAR), missing at random (MAR), and missing not at random (MNAR). Under MCAR, one could simply exclude the missing data from the analysis. However, it may introduce bias if the missing pattern is MAR or MNAR.

To preserve statistical power, one should conduct missing data imputation techniques before the analysis. The single imputation is a simple way that substitutes the mean, mode, or median for the missing data. Unfortunately, this intuitive concept may not capture the variability in the study sample and underestimate the variance, which reduces the correlation between variables or introduces a bias in the inference of the population distribution (2).

The U.S. Census Bureau developed the hot-deck imputation to investigate the missing value of current population income (3), a non-parametric imputation based on Euclidean distance (4). A new way of finding the donor is the random hot-deck, cold-deck, or sequential hot-deck imputation (5). The imputation does not require strong assumptions about the distribution, and it is applied to different types of variables. However, the primary issue is the assumption of MCAR.

The multiple imputations perform better than the simple imputation, but it still requires the assumption of MCAR or MAR (6) based on the multivariate imputation by chained equations or the Markov chain Monte Carlo.

The k-nearest neighbors (KNN) is a simple discriminatory analysis (7). Algorithms of the KNN were studied, and the minimum probability of error was pointed out (8). The KNN also implemented the direct gradient analysis (9). The concept of the training and testing sets using the KNN was further proposed (10). Later, the iterative KNN imputation based on the gray relational analysis was carried out (11). Regarding the truncated data, a previous work developed the KNN-truncated imputation to deal with the chemical compound (12).

The randomness of a decision tree could enhance predictive accuracy (13), and a random forest is a powerful tool for classification problems (14). The missing data imputation is the “rfImpute” function of the “randomForest” package. We denoted it as *RF*_*prx*_ in the simulation study. It is based on the proximity matrix to update the imputation of the missing values. For continuous predictors, the imputed value is the weighted average of the non-missing observations, where the weights are the proximities.

The “missForest” imputation is non-parametric missing value imputation using the random forest (15). We denoted it as *RF*_*mf*_ in the simulations. The fast unified random forests for survival, regression, and classification (RF-SRC) solved the problem when estimating the missing data with out-of-bag errors (16). This method not only applies to classification problems and the regression model but also fits the survival analysis. The random forest on-the-fly is the missing data imputation of RF-SRC. We denoted it as *RF*_*otf*_ in the simulation study. Despite the promising development of missing data imputation, none of the strategies further examined the validity of imputed data using the Cox proportional hazard model. In this research, four machine learning-based imputation strategies were compared, including the KNN, *RF*_*prx*_, *RF*_*mf*_, and *RF*_*otf*_.

In this research, we define supervised and unsupervised missing data imputation as the following. The supervised imputation techniques refer to methods that included the outcome variable as predictors to infer the missing data. In contrast, the unsupervised missing imputation is the one that excludes the outcome of interest in the process. The impact of various missing mechanisms, including MCAR, MAR, and MNAR, would be carefully examined under numerous scenarios. In addition to the conventional approach that evaluated the root mean square error (RMSE) or the proportion of falsely classified entries (PFC) of imputed values, we further analyzed the whole imputed data by the Cox proportional hazard model. Type-I errors of the Cox model using imputed data reveal how the imputation technique performs in the survival analysis. If the Type-I error is over 5% of the nominal level, then, the method is invalid.

## Methods

We want to assess how machine learning-based missing data imputation techniques perform in the survival analysis. The Cox proportional hazard model would incorporate the imputed data, and the results under various scenarios demonstrate overall type-I error. Therefore, the first step is to simulate the survival data under the null hypothesis, including the time to the event, censoring status, six continuous, and four categorical predictive variables. It is noted that the 10 predictors denoted as *x*_1_, *x*_2_, ⋯ , *x*_10_ are uncorrelated, and they are not associated (independent) with the two outcome variables. One of the outcome variables, t, denotes the time to the event, and e denotes the censoring status. Note that if “e = 1,” then the subject has an event, and it also means that the individual is not censored. Thus, “e = 0” identifies the censored subject. Each of the four categorical predictors (*x*_1_, *x*_2_, *x*_3_, *x*_4)_ follows a binomial distribution with *p* = 0.5. Each of the six continuous predictors (*x*_5_, *x*_6_, ⋯ , *x*_10)_ follow a normal distribution with the mean of zero and SD of one. The censoring status “e” follows a uniform distribution between zero and one, representing the random censoring. The time to the event “t” follows the exponential distribution with λ = 0.5. We employed four categorical and six continuous unrelated variables to assess the validity of various methods. The reason is that if under such a simplistic scenario, a strategy could not yield a valid estimate or result, it is unrealistic that the method would be valid under a more complicated structure.

The second step is to assign missing values for the two predictors. One of the predictors (*x*_1_) is categorical, and the other one (*x*_5_) is continuous. Each scenario simulated 1,000 repetitions. Parameters included the sample size (100, 250, 500, and 1,000), the overall missing rate (10, 20, and 30%), and missing mechanisms (MCAR, MAR, and MNAR). Hence, we carefully examined a total of 36 scenarios for the 4 imputation strategies. It is noted that within each overall missing rate, the weights of missingness are 0.2 (*x*_1_ is missing), 0.4 (*x*_5_ is missing), and 0.4 (both *x*_1_ and *x*_1_ are missing), respectively.

Two statistics evaluate the four machine learning-based imputation methods, including the RMSE for the continuous variable and the PFC for the categorical variable. This research examines the performance of imputed data in survival analysis based on the overall type-I error of the Cox model.

Root mean square error is a measure used to measure the difference between the imputed value and the actual value for continuous outcomes. A smaller RMSE indicates a smaller prediction error. The equation is $RMSE=\sqrt{\frac{1}{m}\overset{m}{\underset{i=1}{\sum }}{\left(y_{i}-\hat{y_{i}}\right)}^{2}}$, where m represents the number of missing values, *y*_*i*_ is the actual value, and $\hat{y_{i}}$ is the imputed value.

The PFC is used to determine the imputation situation of category variables. The PFC equation is given by, $PFC=\frac{\sum count\left(y_{i}\neq \hat{y_{i}}\right)}{\sum count\left(y_{i}\right)}$. The denominator is the number of missing values of the categorical variable, and the numerator is the number of imputed values that are not identical to the actual values. The PFC ranges from 0 to 1, and the smaller the value means better imputation.

In addition to the RMSE and PFC, this research further examines the type I error of the Cox model using the imputed data. The likelihood ratio test derives the type-I error. In this way, the type-I error could reveal the impact of imputation on the correlation structure between the predictors and the two survival outcome variables. Finally, we recorded the computation speed that tells the practicality of the different strategies. In this research, we selected machine learning-based imputation strategies that may or may not be suitable for the survival data. In addition, we considered models that any researcher could implement effortlessly. Thus, the KNN and random forest were selected.

The programming language used in this study is R language, version 3.6.1 [(17). R: A language and environment for statistical computing. R Foundation for Statistical Computing]. The Supplementary Materials of the R code (user_utility.r and main.r) listed packages used to simulate the study samples, missing mechanisms, and the imputation methods. The “VIM” package implemented the KNN. The three packages “randomForest,” “randomForestSRC,” and “missForest” are the random forest-based imputation methods. We clarified some notations as to the following: *RF*_*prxt*_ included time to the event as the continuous outcome to generate the proximity matrix. *RF*_*prxe*_ treated the censoring status as the categorical outcome and calculated the proximity matrix. *RF*_*mf*_ excluded both time to the event and the censoring status in the imputation procedure. *RF*_*mfy*_ included both time to the event and the censoring status as two more predictors when inferring the missing values in the dataset. *RF*_*otf*_ is designed for survival analysis; thus, it included both time to the event and the censoring status when inferring the missing values. In summary, *RF*_*mf*_ is an unsupervised imputation, *RF*_*prxt*_ and *RF*_*prxe*_ are partially supervised imputation methods, and KNN, *RF*_*mfy*_, and *RF*_*otf*_ are the three supervised imputation techniques.

## Results

**Figure 1:**
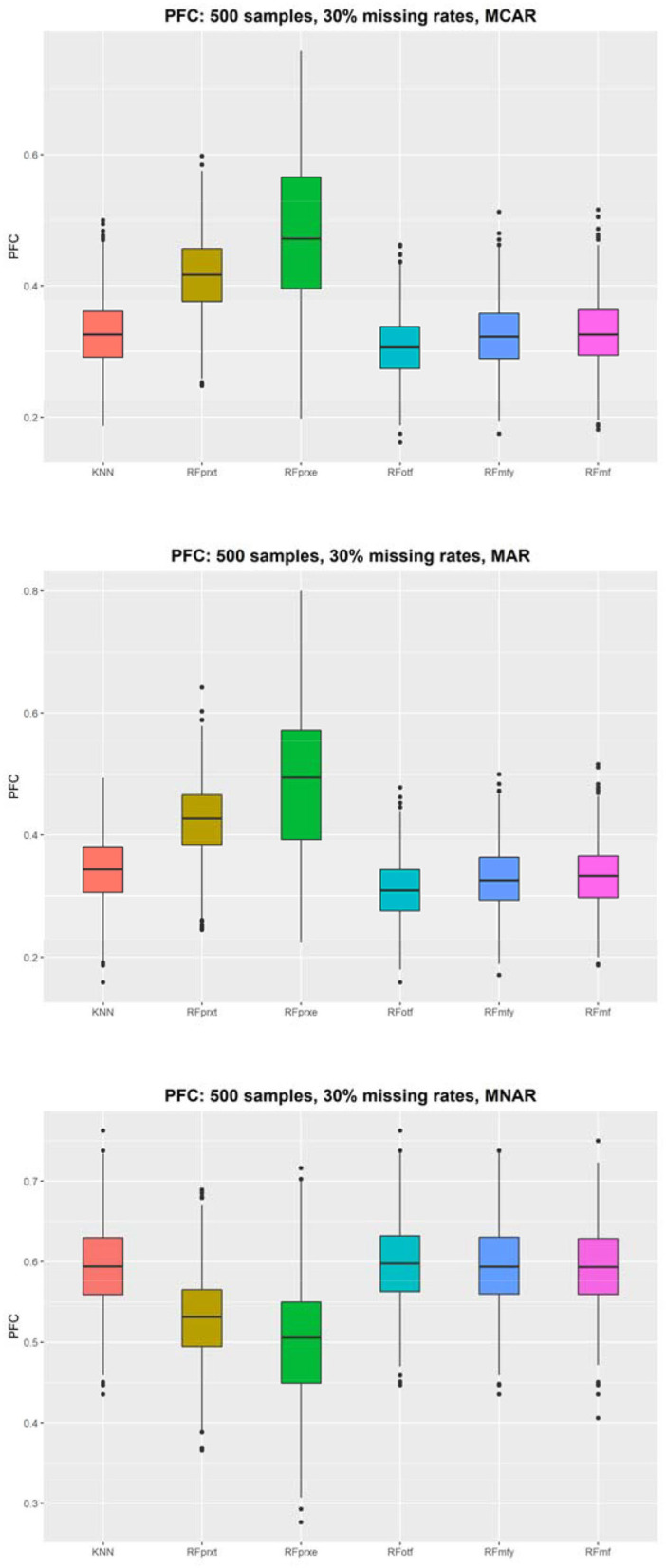
The proportion of falsely classified (PFC) using 500 subjects.

The simulations were conducted under the null hypothesis that 10 predictors and the survival outcome are independent. However, the missing mechanism, MNAR, altered the correlation structure that introduced the dependence between the complete data and imputed values. When the model failed to adjust for the condition, the independent variables and the outcome are correlated under MAR. Therefore, this study has 36 scenarios, and each presents a comparison between four methods. Figure 1 displays the distribution of the PFC using 500 samples. Supplementary Materials displayed results based on different sample sizes and missing rates that yielded a similar pattern. The *RF*_*otf*_ has the best performance in the absence of MCAR and MAR. The *RF*_*prxe*_ has the best performance in the absence of MNAR, but the accuracy is ~0.5, which means that the predictive accuracy is not satisfying.

**Figure 2:**
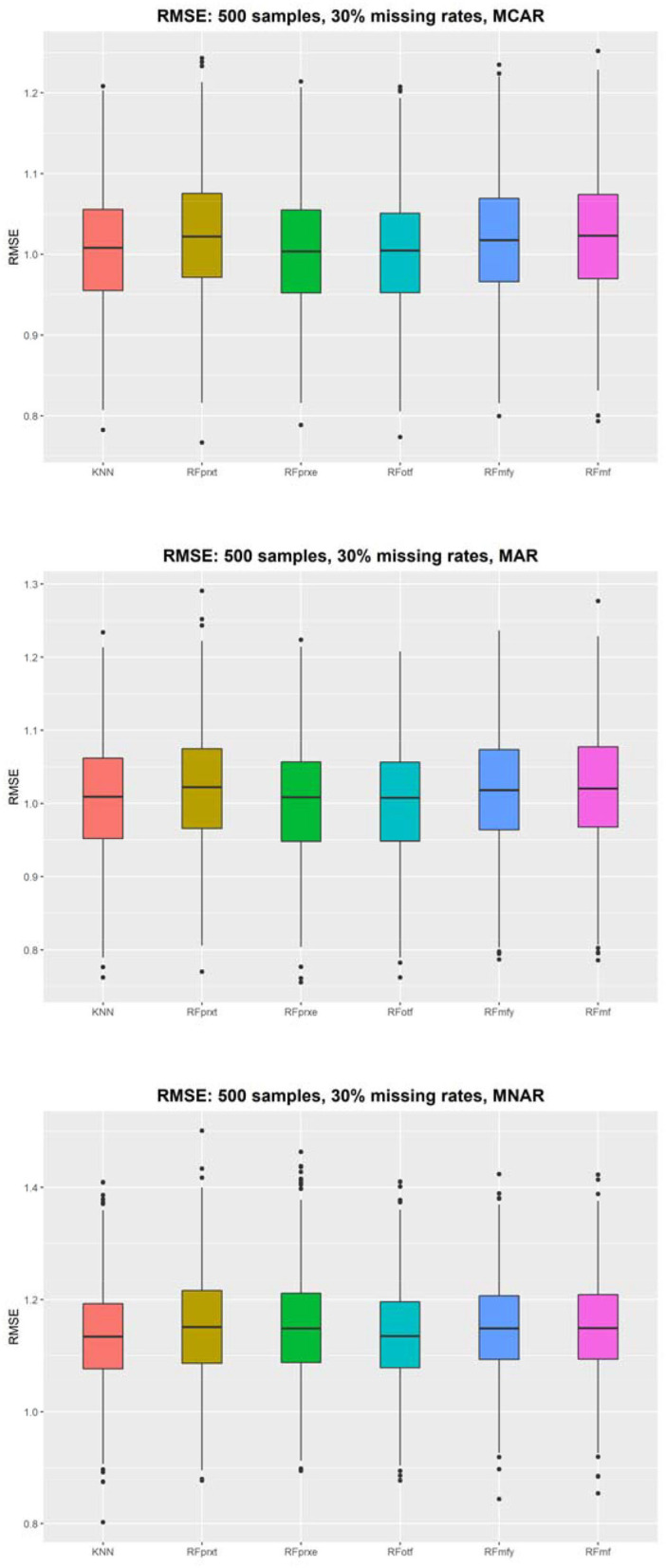
The root means square error (RMSE) using 500 subjects.

Figure 2 shows that the RMSE evaluates the imputation accuracy of a continuous variable and the distributions of the RMSE using 500 samples. The results with different sample sizes and missing rates yielded a similar pattern (refer to Supplementary Materials). KNN performs the best in each scenario, but the differences between the KNN and other random forest-based imputation methods are not discernable. The size of RMSE is approximately one SD. When the missing rate is higher, the gap of RMSE among the four methods will be smaller. The RMSE decreased from 0.08 to 0.01. The *RF*_*otf*_ is similar to the KNN. The higher the missing rate is, the higher the PFC and RMSE, which means that the higher missing rate decreases the imputation accuracy.

The KNN consistently performs better in RMSE, but the superiority is minor. It is not easy to identify the most prominent method in Figure 2. Regarding the PFC, the best performer is the *RF*_*otf*_, because the two outcome variables, “time to the event, t” and “censoring status, e,” are incorporated in the random survival forest. Table 1 summarizes the best performer of the RMSE and PFC under different scenarios.

**Table 1:** The best performer for proportion of falsely classified (PFC) and root means square error (RMSE).

***n***	**Missing rate**	**Missing pattern**	**PFC**	**RMSE**
100	0.1	MCAR	RF_otf_ (0.3062)	KNN (0.8781)
100	0.1	MAR	RF_otf_ (0.3147)	KNN (0.8744)
100	0.1	MNAR	RF_prxe_ (0.4983)	KNN (1.0584)
100	0.2	MCAR	RF_otf_ (0.3071)	KNN (0.9522)
100	0.2	MAR	RF_otf_ (0.3184)	KNN (0.9519)
100	0.2	MNAR	RF_prxe_ (0.4862)	KNN (1.1101)
100	0.3	MCAR	RF_otf_ (0.3049)	KNN (0.9871)
100	0.3	MAR	RF_otf_ (0.3058)	KNN (0.9942)
100	0.3	MNAR	RF_prxe_ (0.4998)	KNN (1.0973)
250	0.1	MCAR	RF_otf_ (0.396)	KNN (0.9603)
250	0.1	MAR	RF_otf_ (0.3069)	KNN (0.9669)
250	0.1	MNAR	RF_prxe_ (0.4987)	KNN (1.1751)
250	0.2	MCAR	RF_otf_ (0.3022)	KNN (0.9977)
250	0.2	MAR	RF_otf_ (0.3079)	KNN (0.9938)
250	0.2	MNAR	RF_prxe_ (0.4999)	KNN (1.1489)
250	0.3	MCAR	RF_otf_ (0.3107)	KNN (1.0083)
250	0.3	MAR	RF_otf_ (0.3143)	KNN (1.0099)
250	0.3	MNAR	RF_prxe_ (0.4971)	KNN (1.1278)
500	0.1	MCAR	RF_otf_ (0.3114)	KNN (0.98)
500	0.1	MAR	RF_otf_ (0.3057)	KNN (0.9852)
500	0.1	MNAR	RF_prxe_ (0.5072)	KNN (1.2091)
500	0.2	MCAR	RF_otf_ (0.3069)	KNN (1.0045)
500	0.2	MAR	RF_otf_ (0.307)	KNN (1.0044)
500	0.2	MNAR	RF_prxe_ (0.5046)	KNN (1.1735)
500	0.3	MCAR	RF_otf_ (0.3073)	KNN (1.01)
500	0.3	MAR	RF_otf_ (0.3087)	KNN (1.0098)
500	0.3	MNAR	RF_prxe_ (0.502)	KNN (1.1367)
1,000	0.1	MCAR	RF_otf_ (0.3093)	KNN (0.9958)
1,000	0.1	MAR	RF_otf_ (0.3067)	KNN (0.9963)
1,000	0.1	MNAR	RF_prxe_ (0.5272)	KNN (1.2208)
1,000	0.2	MCAR	RF_otf_ (0.3074)	KNN (1.0034)
1,000	0.2	MAR	RF_otf_ (0.3102)	KNN (1.0056)
1,000	0.2	MNAR	RF_prxe_ (0.5274)	KNN (1.1766)
1,000	0.3	MCAR	RF_otf_ (0.3089)	KNN (1.0081)
1,000	0.3	MAR	RF_otf_ (0.31)	KNN (1.0102)
1,000	0.3	MNAR	RF_prxe_ (0.5142)	KNN (1.1385)

Type-I error of the Cox proportional hazard model under different situations further evaluated the overall performance of each imputation strategy (Table 2). This step is crucial since the comparisons between the PFC and RMSE after imputation could not warrant a valid Cox regression analysis. There are some scenarios where the results of *RF*_*otf*_ and *RF*_*mf*_ are very close, but the *RF*_*otf*_ is consistently larger than the *RF*_*mf*_. In conclusion, the overall performance of the *RF*_*mf*_ method is the best, a non-parametric and unsupervised imputation method that excludes the two survival outcome variables (t and e). It is noted that the *RF*_*prxe*_ and *RF*_*prxt*_ have much inflated type-I error, since this type of imputation considers only one dependent variable (time to the event or censoring status) when constructing the proximity matrix. However, the simulation study was based on survival data with two outcome variables. Therefore, including one of the two survival outcome variables will result in an inflated type-I error. We highly recommended avoiding the “rfImpute” function in survival data. The KNN imputation also demonstrated inflated type-I errors and should not be used for survival analysis.

**Table 2:** The type-I error of the Cox model.

**Sample**	**Missing rate**	**Missing mechanism**	**Complete data**	**KNN**	**RF_**prxt**_**	**RF_**prxe**_**	**RF_**otf**_**	**RF_**mfy**_**	**RF_**mf**_**
100	0.1	MCAR	0.079	0.088	0.085	0.092	0.082	0.088	0.08
100	0.1	MAR	0.084	0.095	0.094	0.098	0.09	0.094	0.085
100	0.1	MNAR	0.087	0.093	0.095	0.093	0.09	0.091	0.089
100	0.2	MCAR	0.091	0.092	0.102	0.113	0.081	0.095	0.079
100	0.2	MAR	0.076	0.083	0.089	0.105	0.073	0.085	0.073
100	0.2	MNAR	0.075	0.08	0.09	0.106	0.075	0.082	0.074
100	0.3	MCAR	0.085	0.101	0.12	0.134	0.094	0.114	0.086
100	0.3	MAR	0.072	0.092	0.114	0.13	0.087	0.107	0.078
100	0.3	MNAR	0.085	0.105	0.114	0.136	0.092	0.104	0.086
250	0.1	MCAR	0.055	0.057	0.063	0.069	0.051	0.059	0.051
250	0.1	MAR	0.06	0.066	0.079	0.089	0.064	0.072	0.064
250	0.1	MNAR	0.054	0.059	0.072	0.083	0.054	0.058	0.053
250	0.2	MCAR	0.052	0.065	0.082	0.095	0.055	0.068	0.055
250	0.2	MAR	0.062	0.078	0.093	0.117	0.069	0.087	0.067
250	0.2	MNAR	0.054	0.072	0.087	0.115	0.059	0.068	0.056
250	0.3	MCAR	0.069	0.094	0.125	0.169	0.075	0.097	0.07
250	0.3	MAR	0.059	0.09	0.126	0.153	0.073	0.09	0.064
250	0.3	MNAR	0.07	0.083	0.126	0.165	0.069	0.089	0.059
500	0.1	MCAR	0.061	0.057	0.073	0.082	0.058	0.062	0.057
500	0.1	MAR	0.051	0.059	0.062	0.077	0.055	0.061	0.055
500	0.1	MNAR	0.068	0.066	0.08	0.096	0.069	0.071	0.066
500	0.2	MCAR	0.05	0.065	0.105	0.141	0.055	0.062	0.053
500	0.2	MAR	0.056	0.069	0.113	0.141	0.055	0.067	0.055
500	0.2	MNAR	0.063	0.072	0.102	0.147	0.064	0.067	0.061
500	0.3	MCAR	0.046	0.065	0.137	0.201	0.053	0.074	0.046
500	0.3	MAR	0.047	0.078	0.154	0.224	0.058	0.079	0.054
500	0.3	MNAR	0.057	0.068	0.131	0.204	0.061	0.071	0.056
1,000	0.1	MCAR	0.053	0.059	0.074	0.094	0.055	0.057	0.053
1,000	0.1	MAR	0.042	0.049	0.077	0.1	0.044	0.048	0.044
1,000	0.1	MNAR	0.047	0.054	0.07	0.081	0.052	0.056	0.051
1,000	0.2	MCAR	0.043	0.054	0.121	0.173	0.048	0.057	0.043
1,000	0.2	MAR	0.048	0.057	0.127	0.191	0.052	0.06	0.051
1,000	0.2	MNAR	0.053	0.065	0.151	0.217	0.06	0.069	0.055
1,000	0.3	MCAR	0.047	0.061	0.21	0.32	0.047	0.07	0.046
1,000	0.3	MAR	0.043	0.066	0.237	0.362	0.049	0.069	0.049
1,000	0.3	MNAR	0.045	0.07	0.217	0.351	0.061	0.071	0.055

The *RF*_*otf*_ includes both times to the event and censoring status in the random survival forest to impute missing values under MCAR or assumption of MAR. Thus, the *RF*_*otf*_ is valid for survival data, and the type-I error behaves well under MCAR and MAR. However, when the missing pattern is MNAR, the *RF*_*otf*_ showed an inflated type-I error.

The type-I error of the *RF*_*mf*_ is lower than *RF*_*mfy*_, which also included time to the event and censoring status as the predictors. This phenomenon is probably due to the missing mechanism of the MNAR, and the conditional missingness introduced correlation between the observed predictors and the two survival outcome variables, time to the event and censoring.

For a small sample study, estimators, in general, have a large variance. As a result, the missing data imputed by *RF*_*mf*_ also showed an inflated type-I error in the Cox model. When the sample size increases, the type-I error decreases and approaches the significance level of 0.05.

Finally, the run time is also studied. In each scenario, the fastest method is the KNN, followed by *RF*_*otf*_, and the rest of the methods are similar. When the sample size is 100, the simulation time of each method is within 0.5 s, where the KNN and *RF*_*otf*_ only spend 0.1 s. When the missing rate is higher and the missing mechanism is MNAR, the run time of two methods is almost identical.

When the sample size is 250, the run time of KNN and *RF*_*otf*_ is <0.5 s, but the other methods take 1–2 s. If the sample size is 500, the KNN only requires 0.5 s, *RF*_*otf*_ takes 1.5 s, and the rest methods take about 5–6 s. When the number of samples is 1,000, the KNN takes about 1 s, *RF*_*otf*_ takes about 5 s, and the other methods take about 15–23 s. The greater the sample size, the more significant difference in the run time between various methods.

In summary, according to the type-I error of the Cox model, the *RF*_*mf*_ strategy that excludes the two survival outcome variables in the imputation procedure is the optimal method. However, if the run time is the only concern, the random forest on-the-fly imputation is better.

## Discussions

This research examined four machine learning-based imputation methods, including the KNN, and three strategies based on the random forest. We proposed the concepts of supervised and unsupervised imputations. Although the RMSE and PFC are similar for the four machine learning-based imputation strategies, type-I error of the Cox model could be inflated dramatically for the different methods under MNAR. Hence, the validity of the Cox model using imputed data changes dramatically under different settings. The simulation results showed that the *RF*_*mf*_ performs the best even under the most challenging situation, MNAR. Therefore, this strategy would be valid under all types of missing mechanisms.

One of the most significant advantages of machine learning is that it is suitable for high-dimensional data, time-series data, or complex data interactions. Although this study focuses on survival data with 10 predictive variables, the concept of supervised or unsupervised imputation and the structure of predictors could be easily extended for different study designs.

Finally, the simulation study is the null hypothesis of the Cox model, where the predictive and survival outcomes are independent. Therefore, the type-I error is the essential tool when comparing performances of the four imputation strategies. Power study is not meaningful since the only valid imputation method is the *RF*_*mf*_. In addition, the rest strategies revealed inflated type-I errors under MNAR.

The R code implemented in the simulations is freely available. We have included the code as Supplementary Materials. The file “USER_UTILITY” is the first program that generates the study samples, missing mechanisms, and imputation strategies. The second file, “MAIN,” generates all statistical results and figures. Researchers could quickly adopt supervised and unsupervised imputations for the four methods by using the two R codes for future applications.

### Limitations

In machine learning, there are many methods for prediction and classification, such as the support vector machine (SVM) (18), extreme gradient boosting machine (XGBoost) (19), and artificial neural network (ANN) (20). In future studies, these methods may also develop novel imputation strategies. Therefore, we did not include the three methods in this research. We simulated the four dichotomous and six continuous predictors as independent variables. A high correlation among them may cause more bias in type-I errors. The categorical predictors could have more levels in simulations, but we expect that the comparisons and patterns between the methods studied in this research are likely to be similar.

This machine learning-based research revealed a robust missing data imputation strategy for survival analysis under various missing mechanisms. The non-parametric “missForest” imputation (*RF*_*mf*_), that excludes the survival time and censoring status from the imputation scheme, could provide valid results using the Cox proportional hazard model under the impact of MCAR, MAR, and MNAR.

## Data Availability Statement

The original contributions presented in the study are included in the article/Supplementary Material, further inquiries can be directed to the corresponding author/s.

## Author Contributions

C-YG proposed the research concept, supervised the project, and wrote the manuscript. Y-CY conducted the analysis and prepared figures and tables. Y-HC jointly supervised the research and provided a scholarship for Y-CY's work. All authors have read and approved the final manuscript.

## Conflict of Interest

The authors declare that the research was conducted in the absence of any commercial or financial relationships that could be construed as a potential conflict of interest.

## Abbreviations

RMSE: root means square error
PFC: proportion of falsely classified entries
MCAR: missing completely at random
MAR: missing at random
MNAR: missing not at random
KNN: k-nearest neighbors
RF-SRC: Random Forests for Survival, Regression, and Classification
SVM: support vector machine
XGBoost: Extreme Gradient Boosting Machine
ANN: artificial neural network.
