# Supplementary material for: The Optimal Machine Learning-Based Missing Data Imputation for the Cox Proportional Hazard Model
Source: Front Public Health. 2021 Jul 5;9:680054. doi: 10.3389/fpubh.2021.680054 (PMC8289437; doi:10.3389/fpubh.2021.680054)

Supplement Materials

Figure S1: PFC with different sample sizes and missing rates


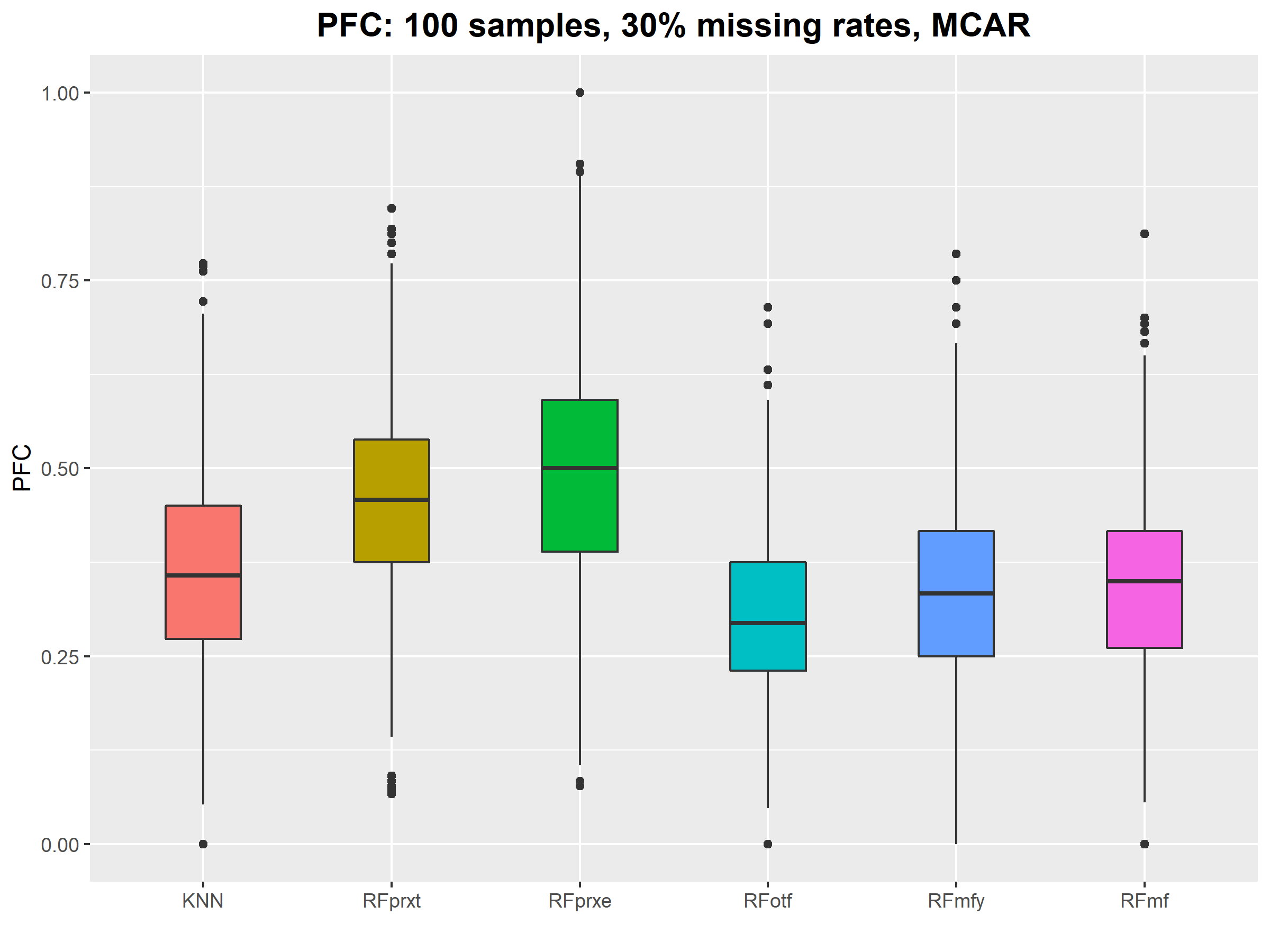

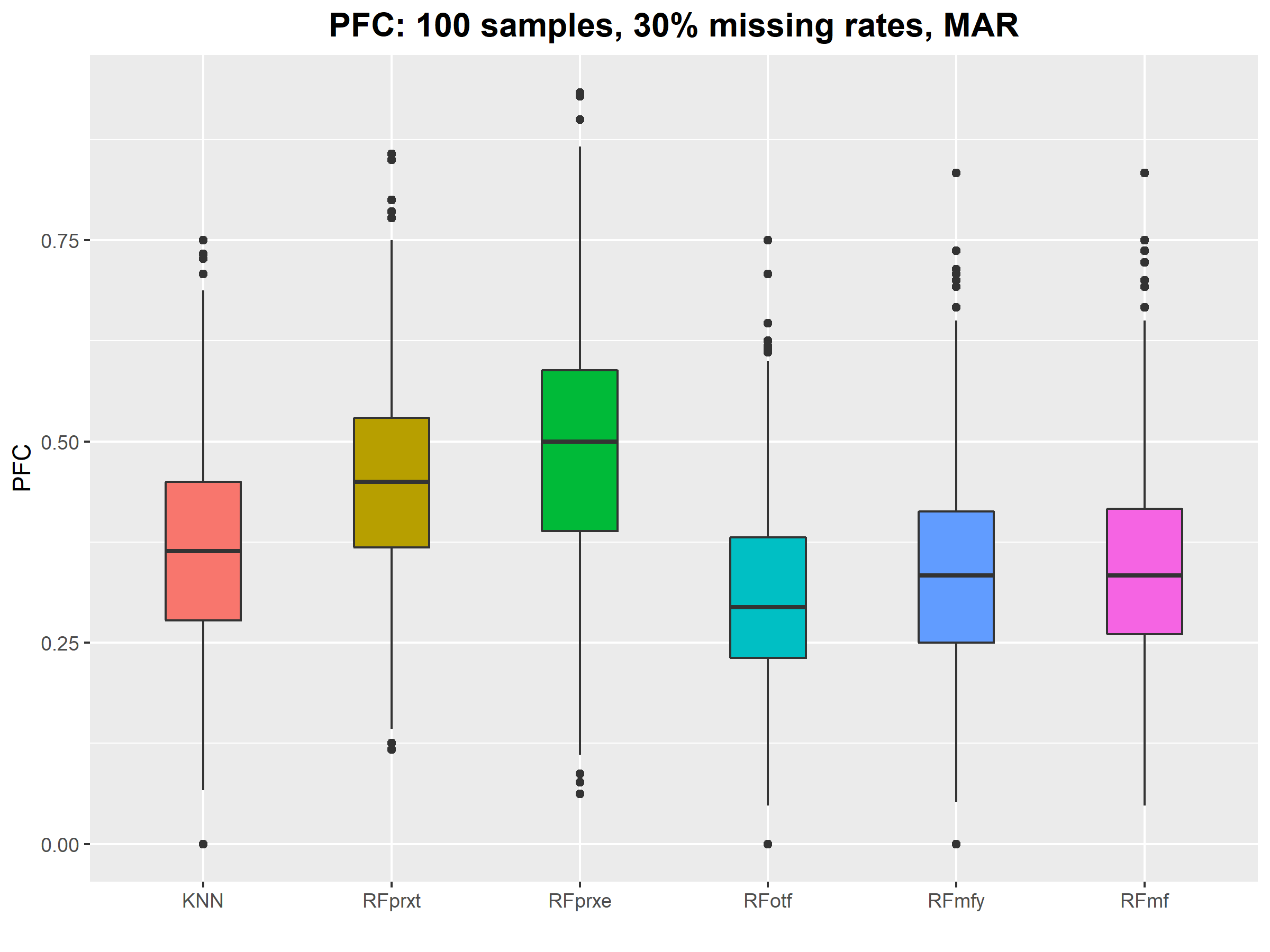

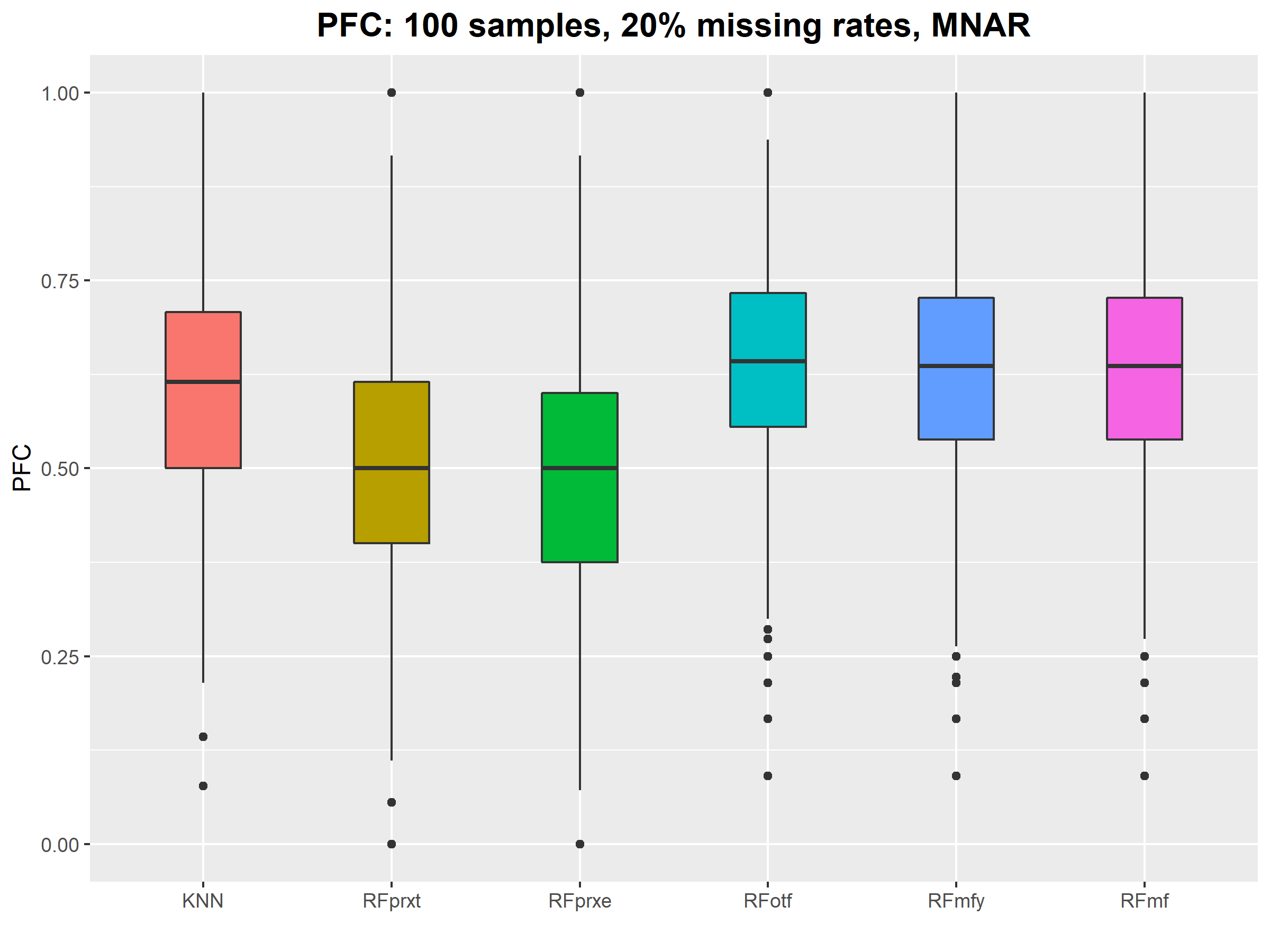

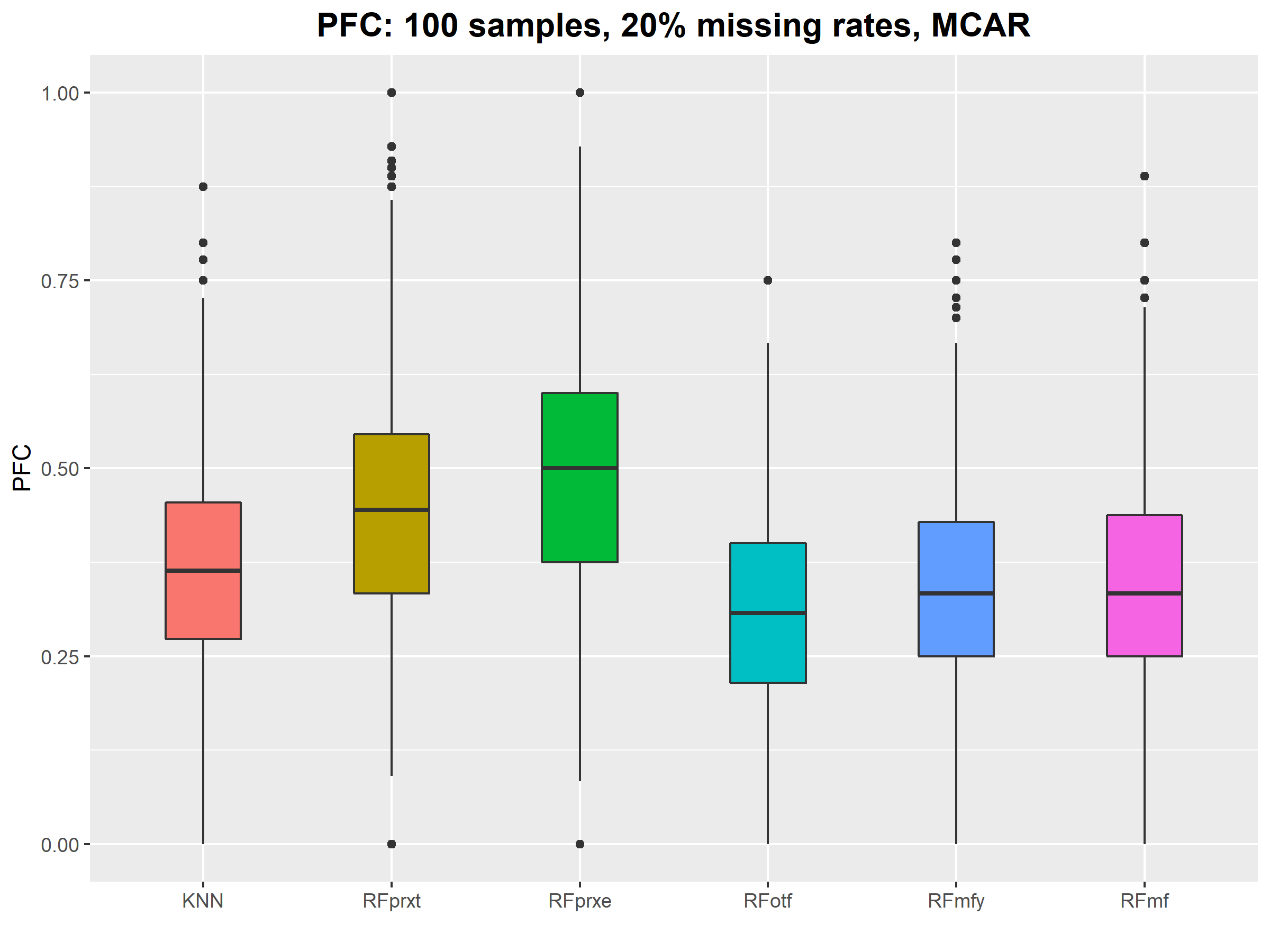

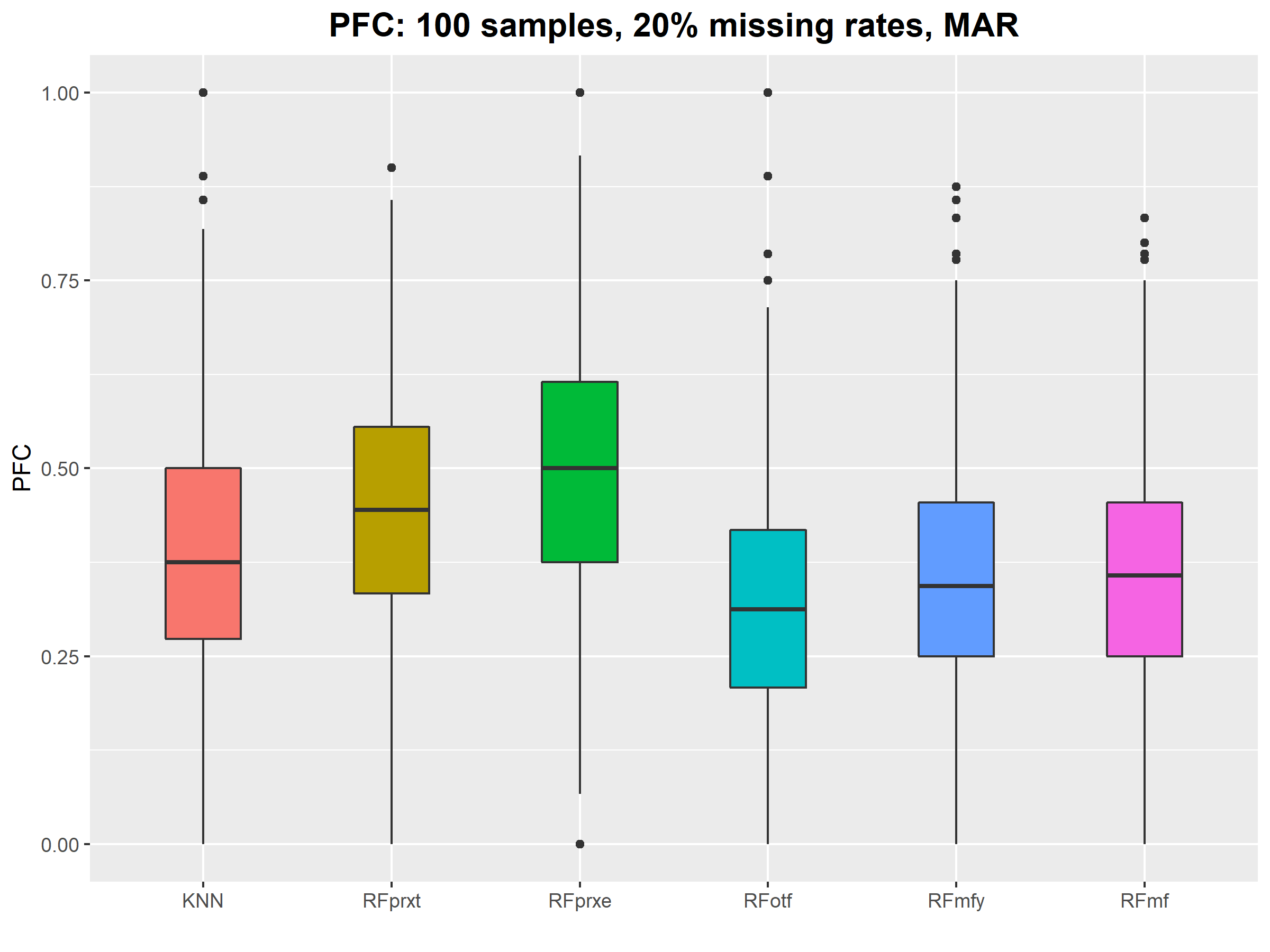

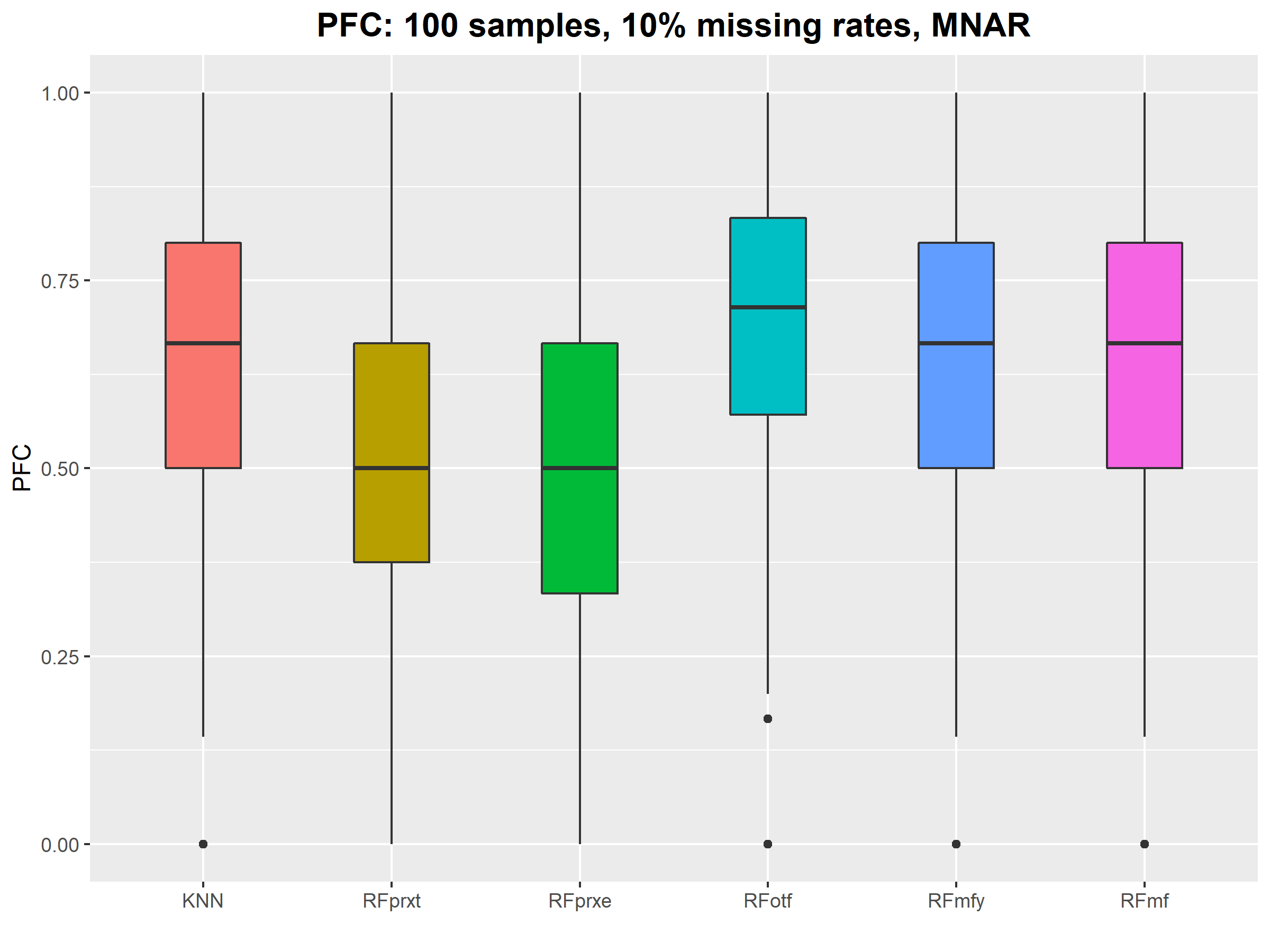

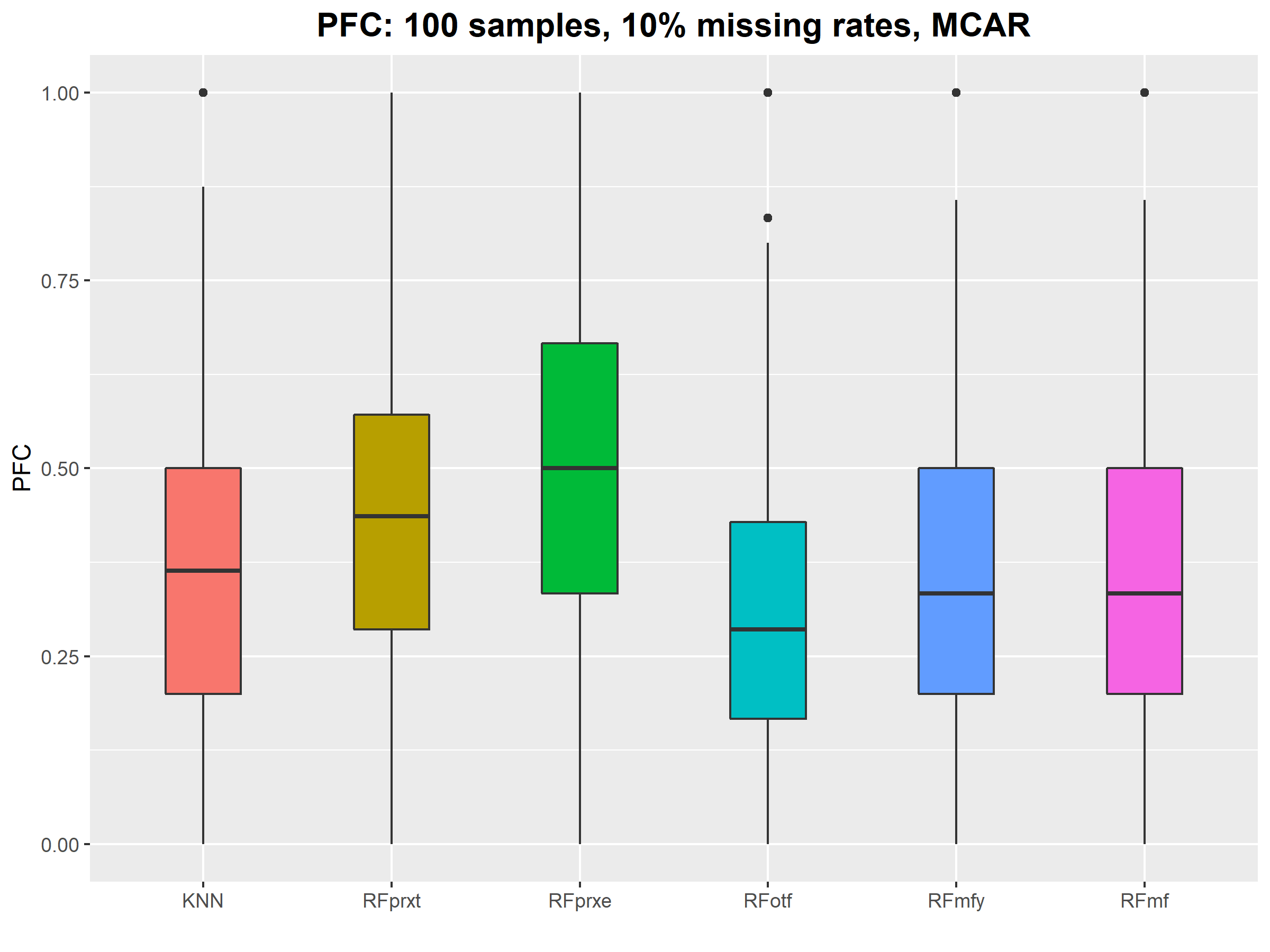

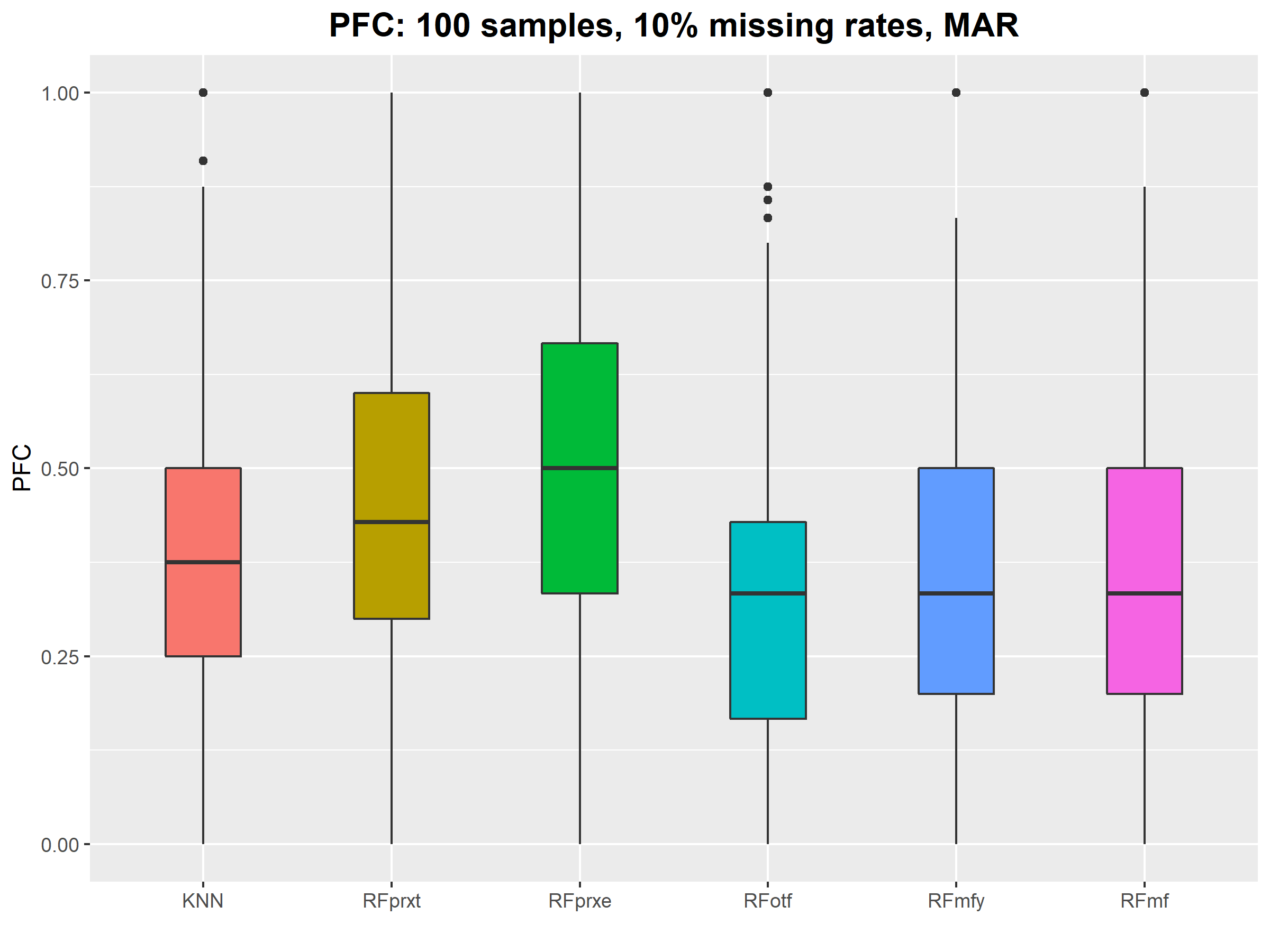

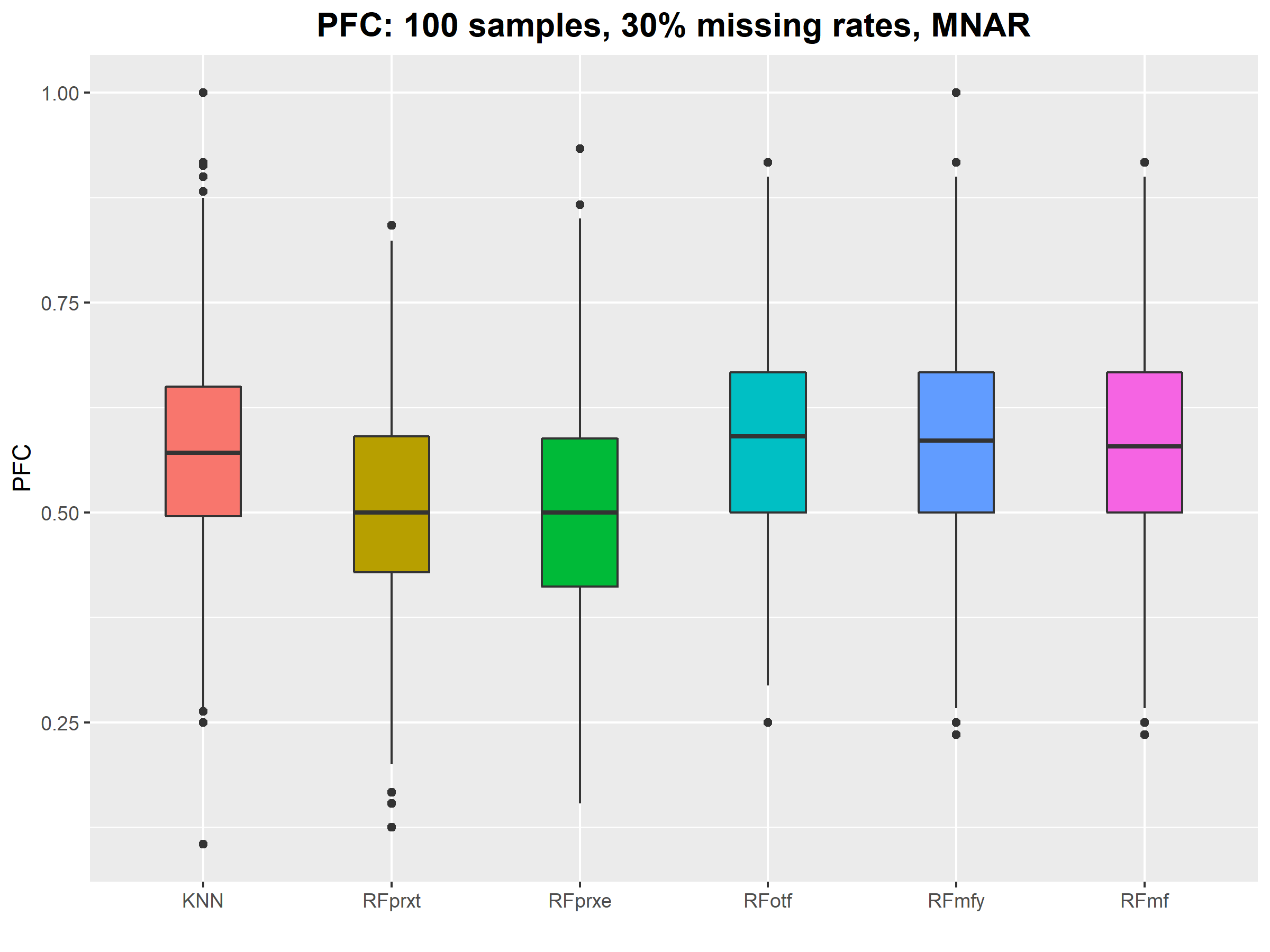


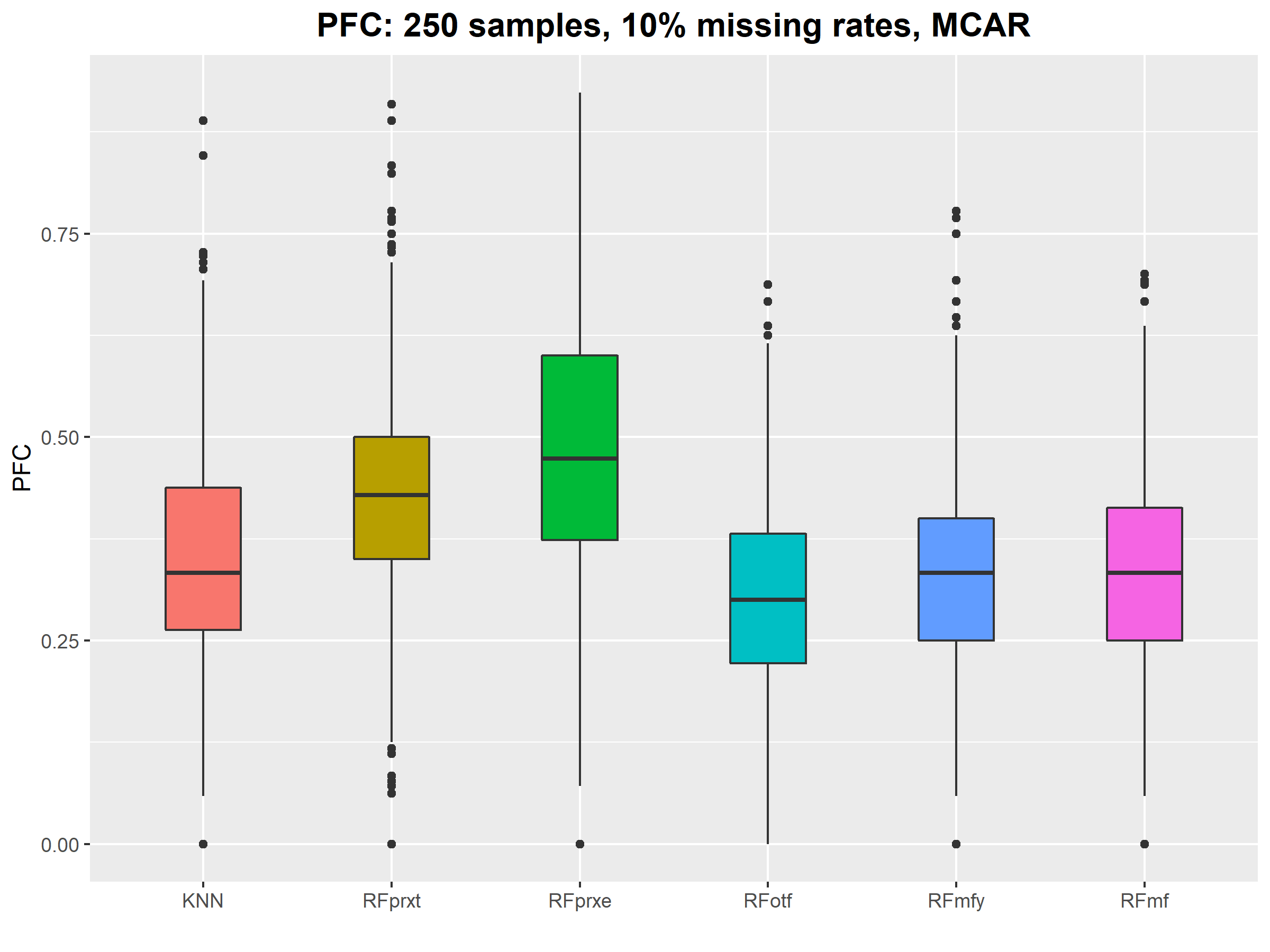

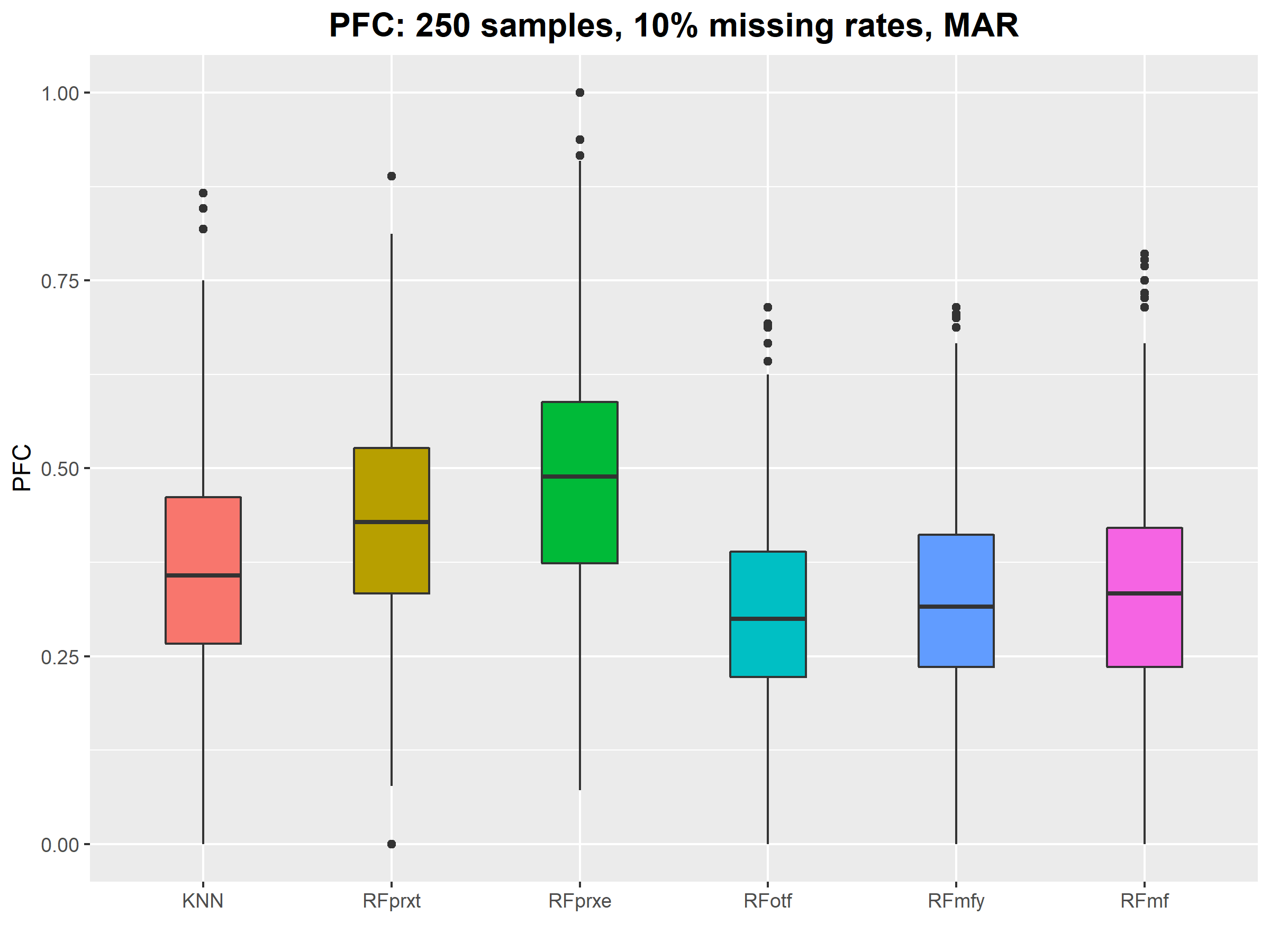

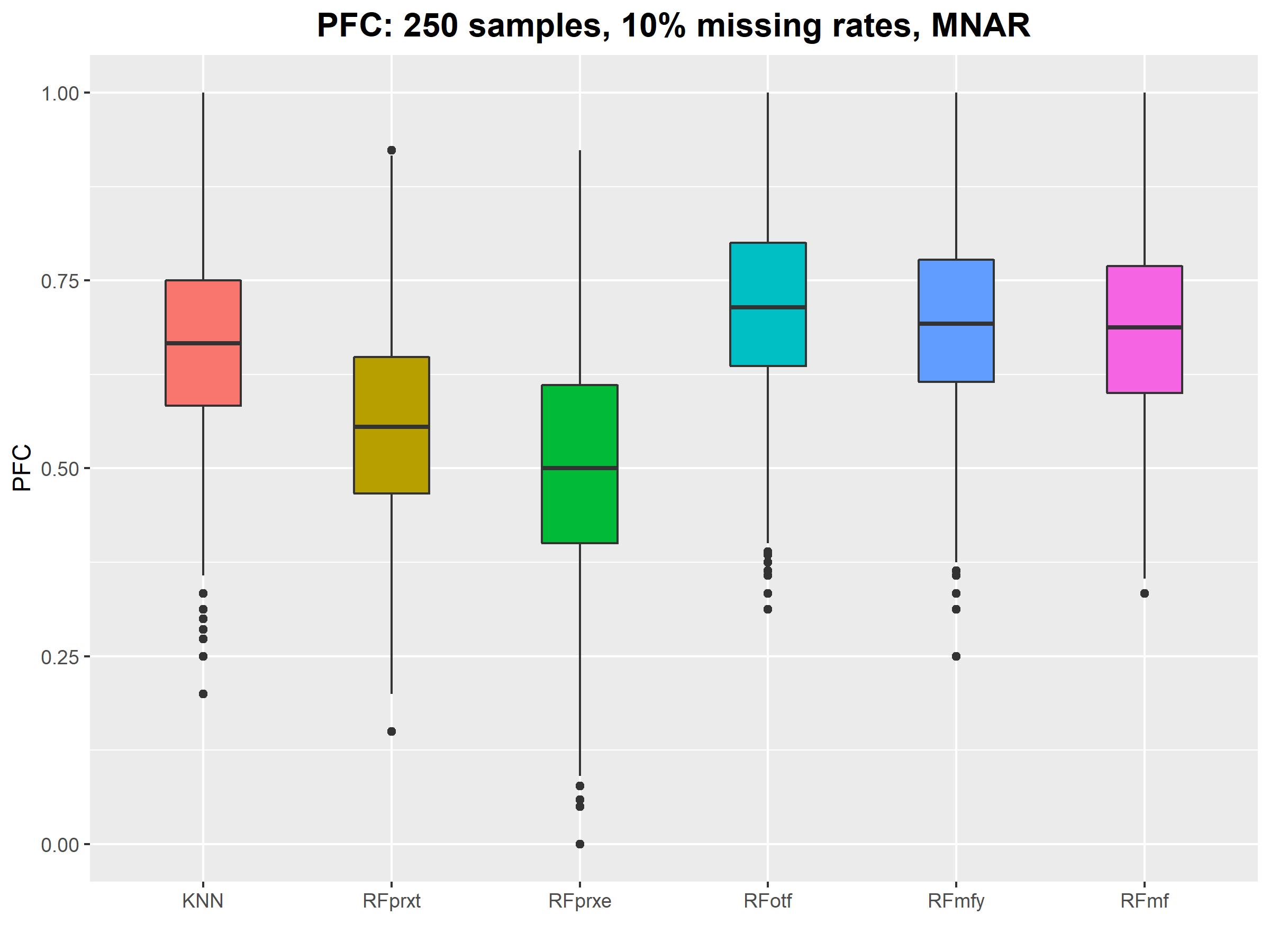

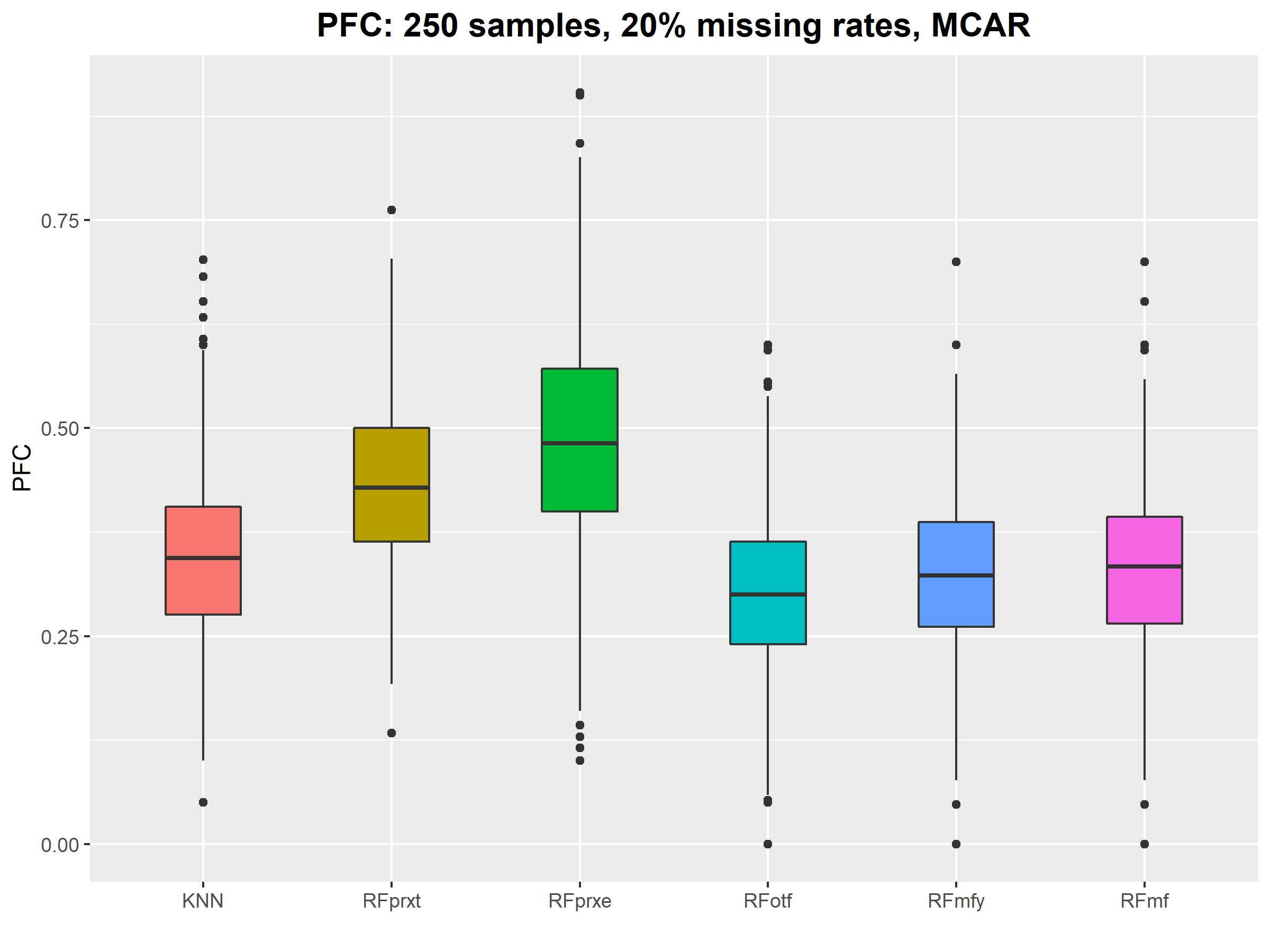

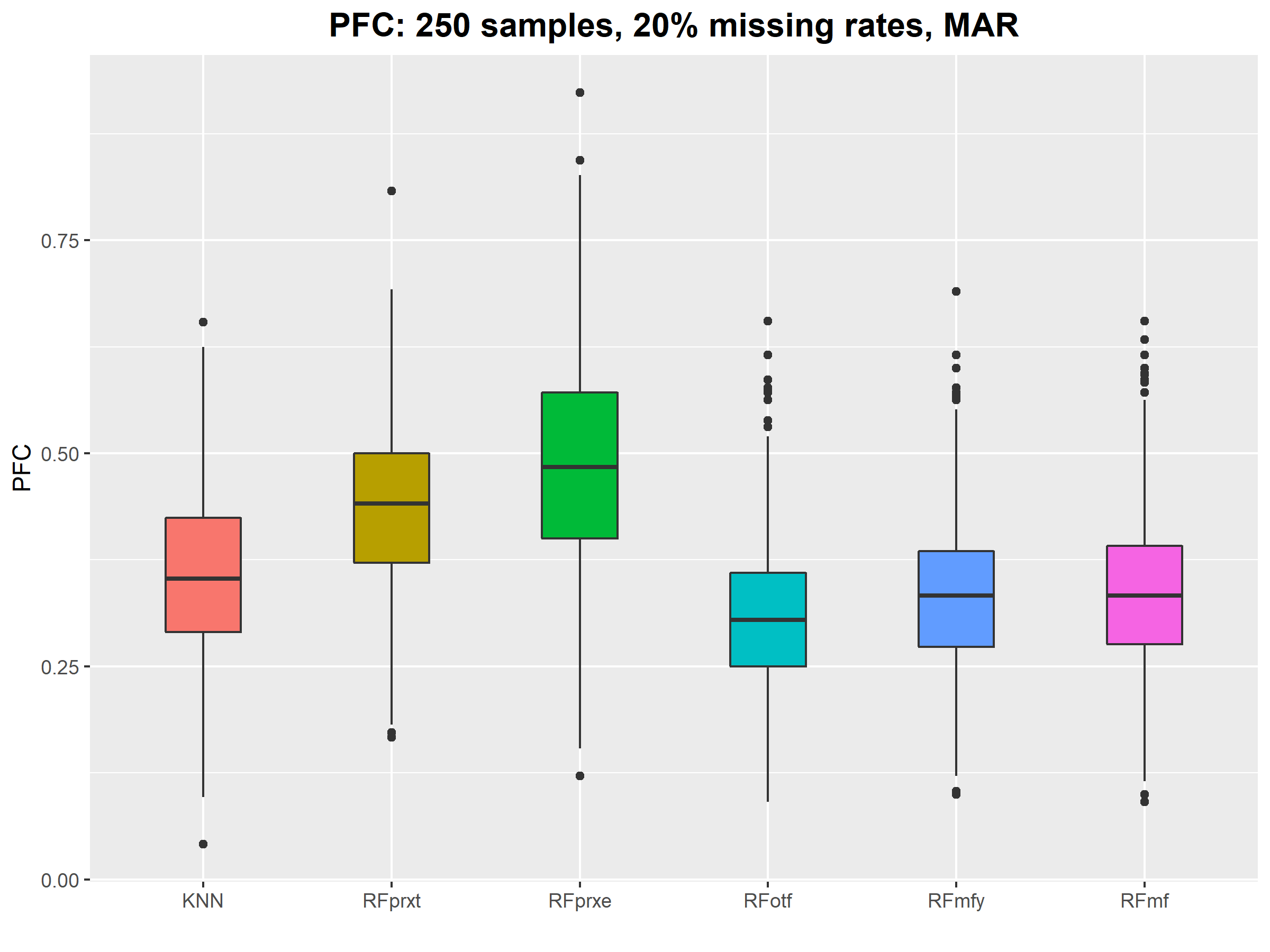

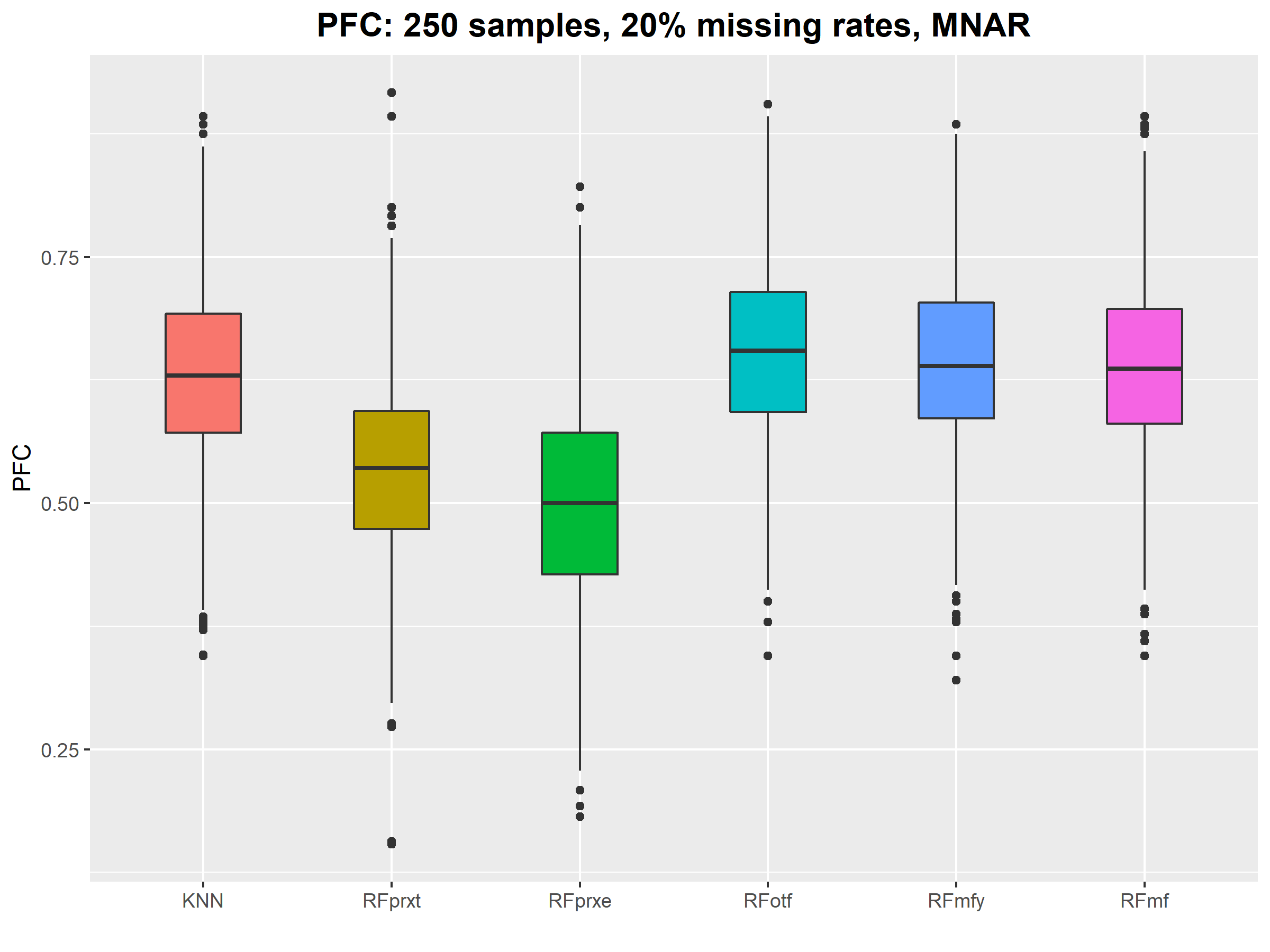

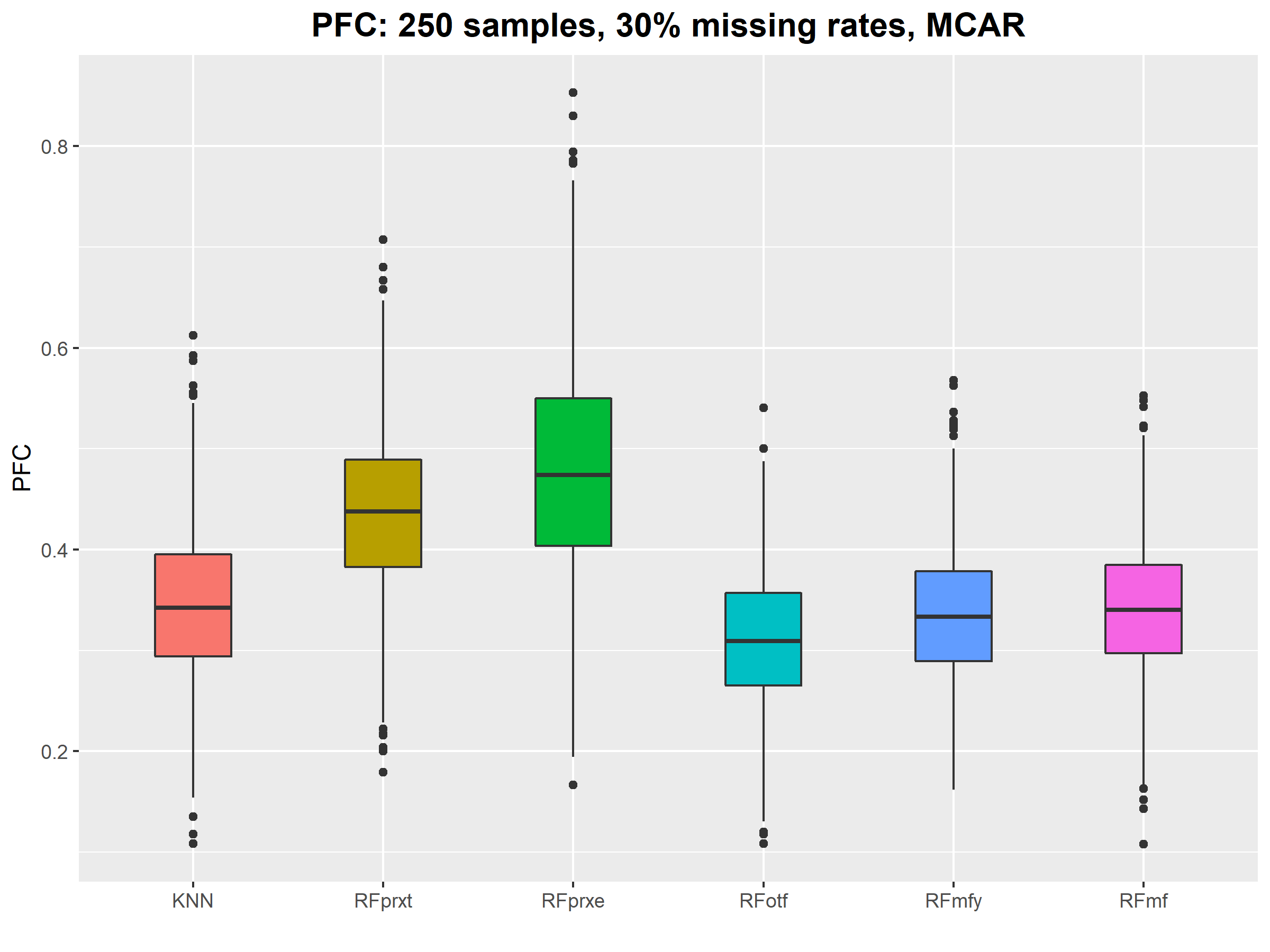

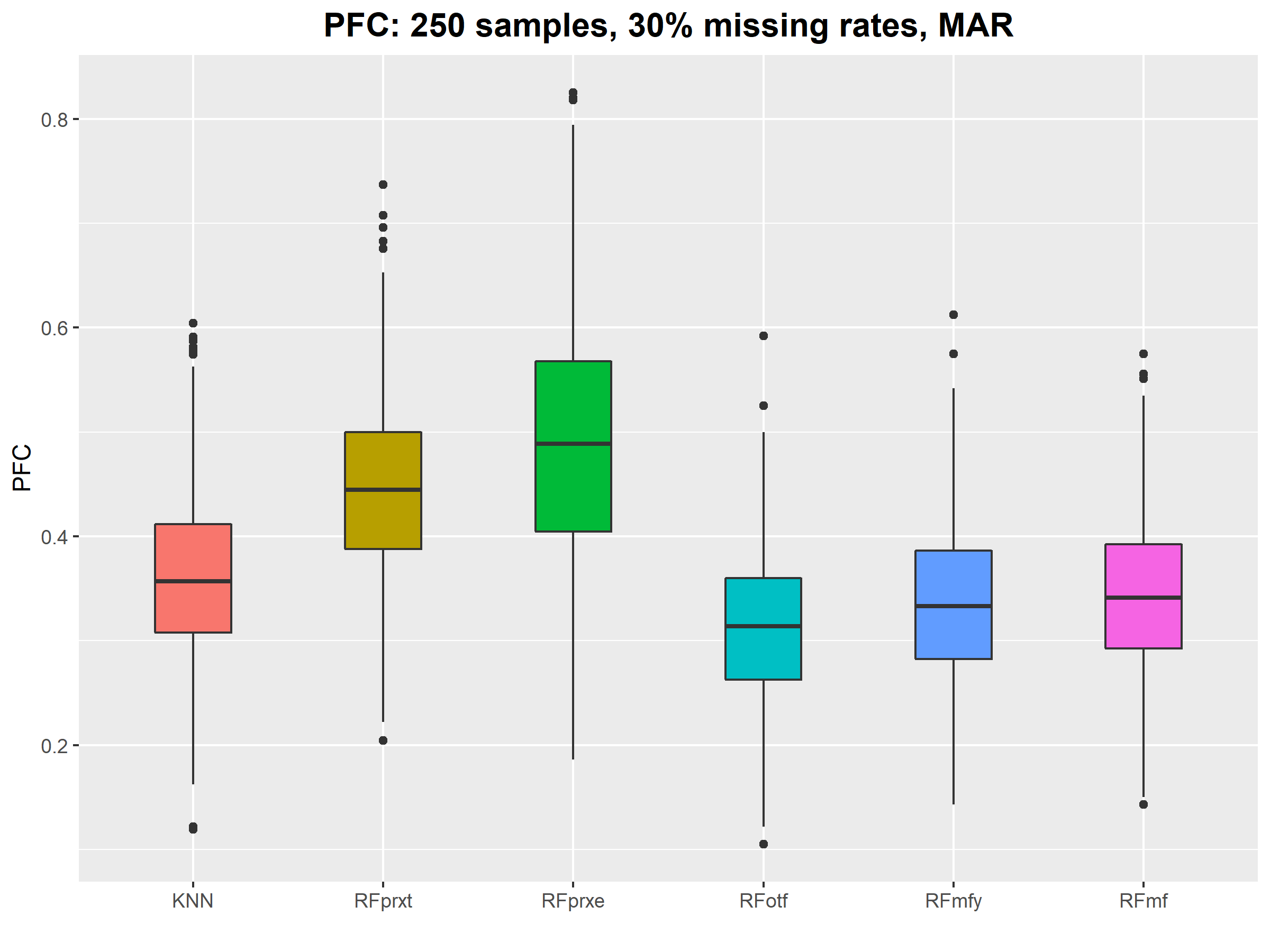

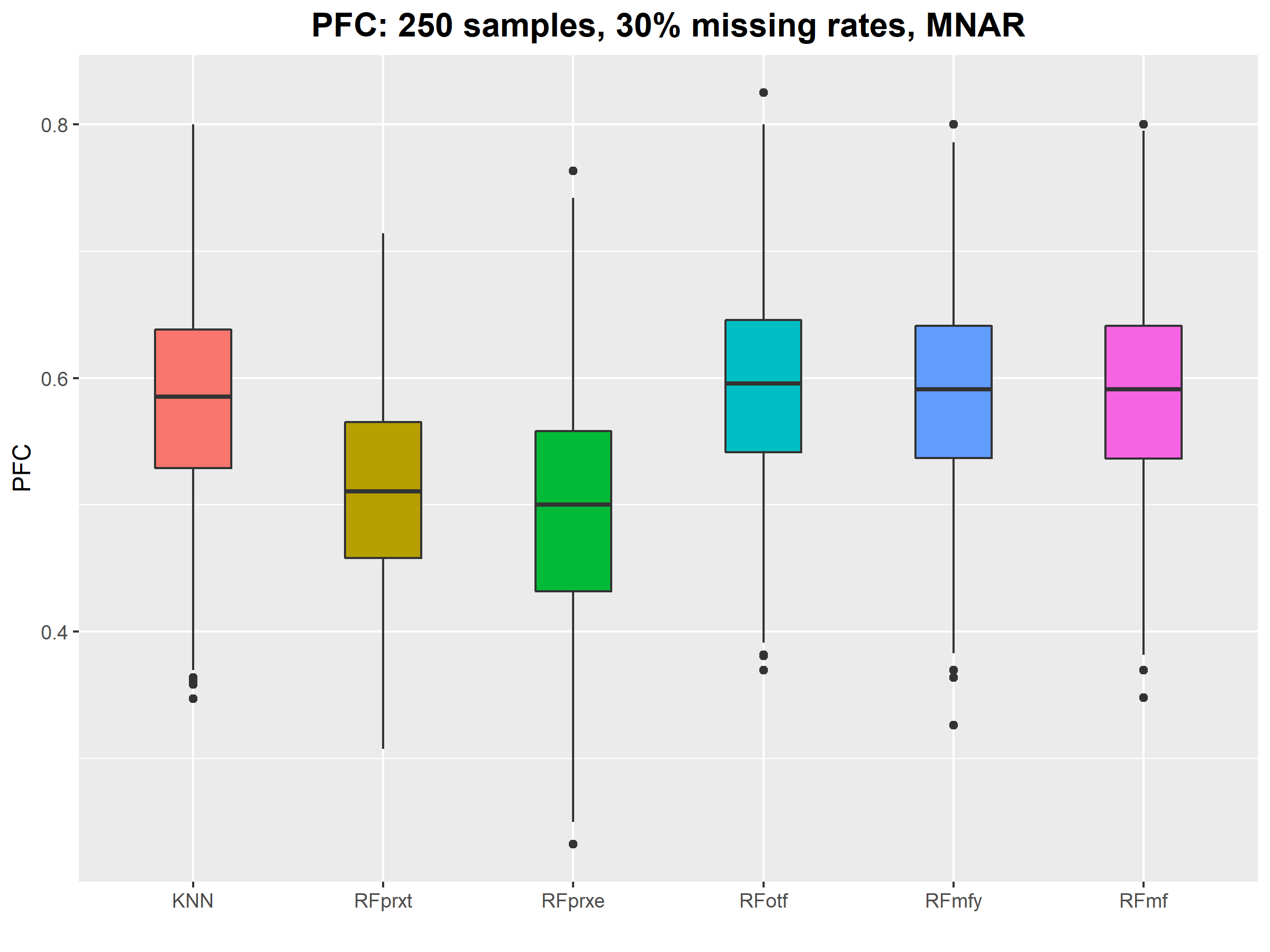


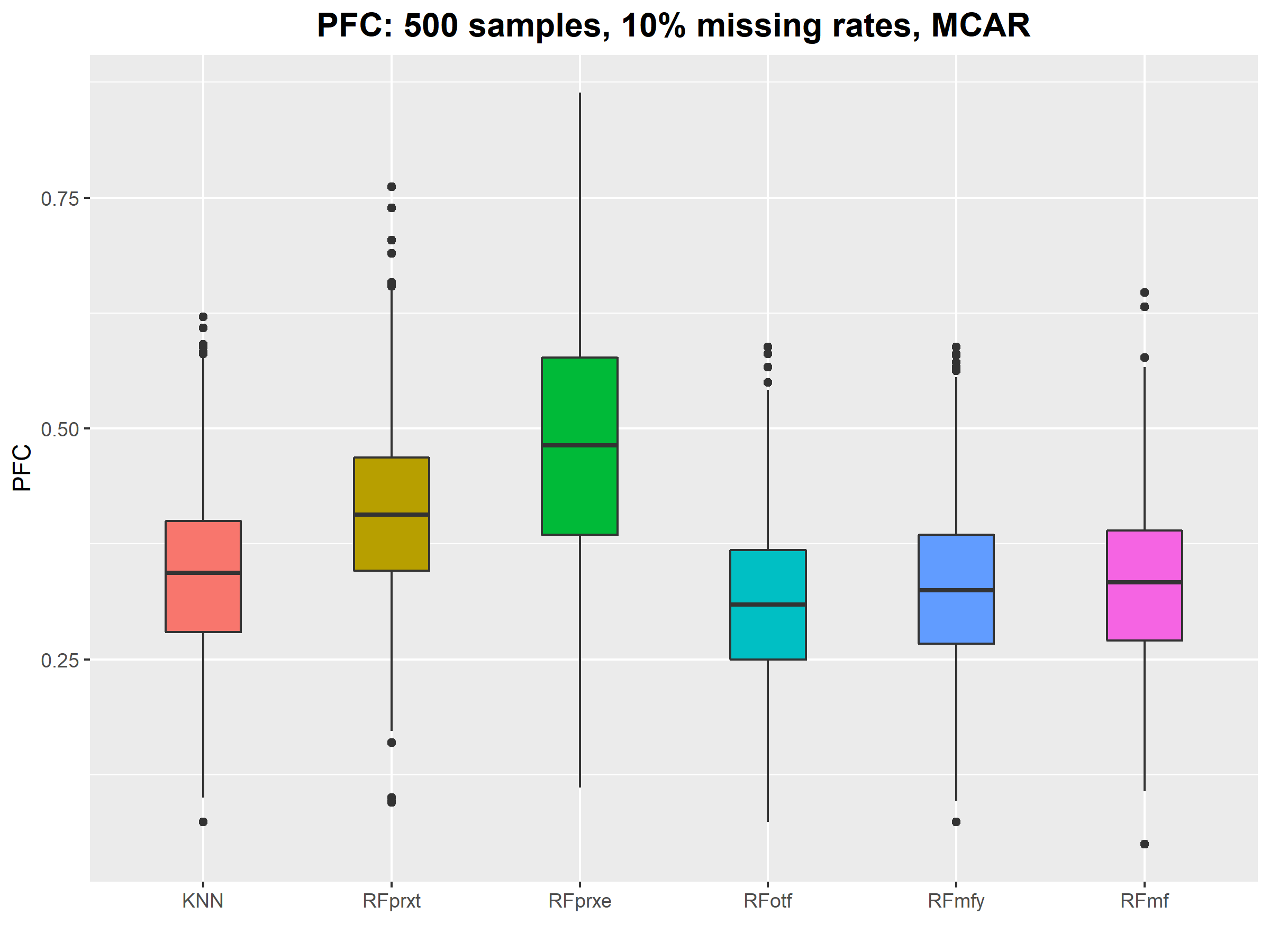

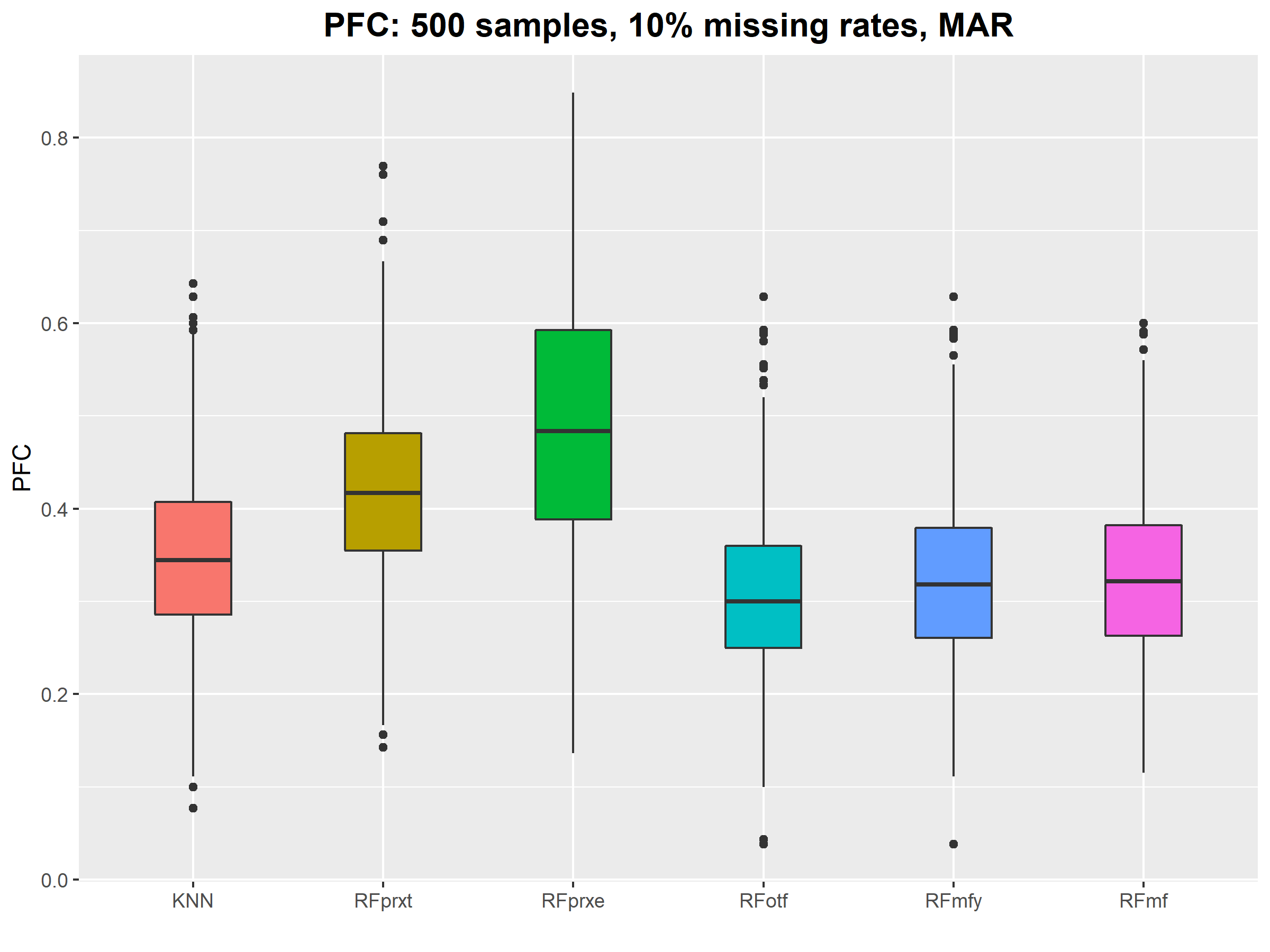

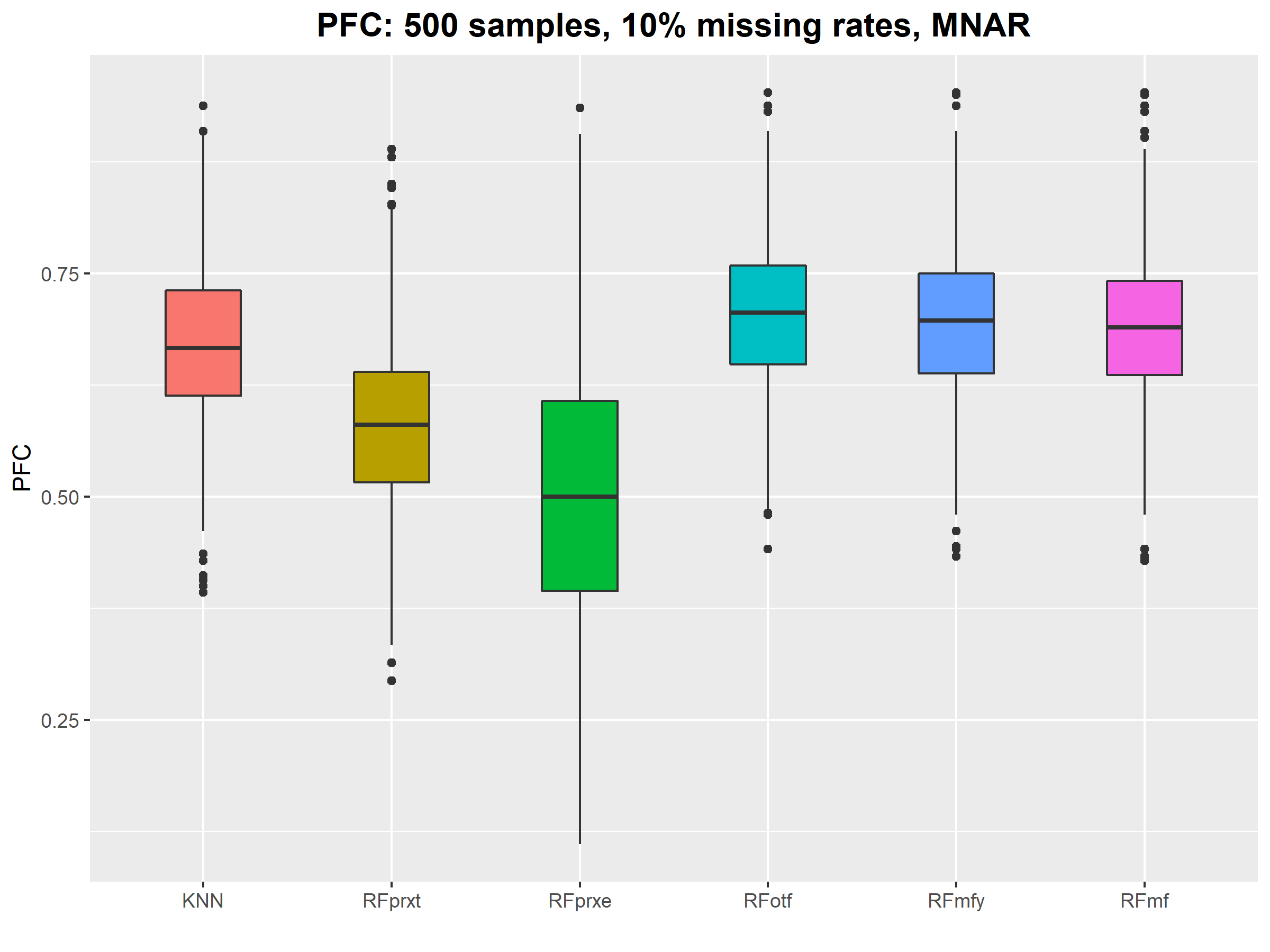

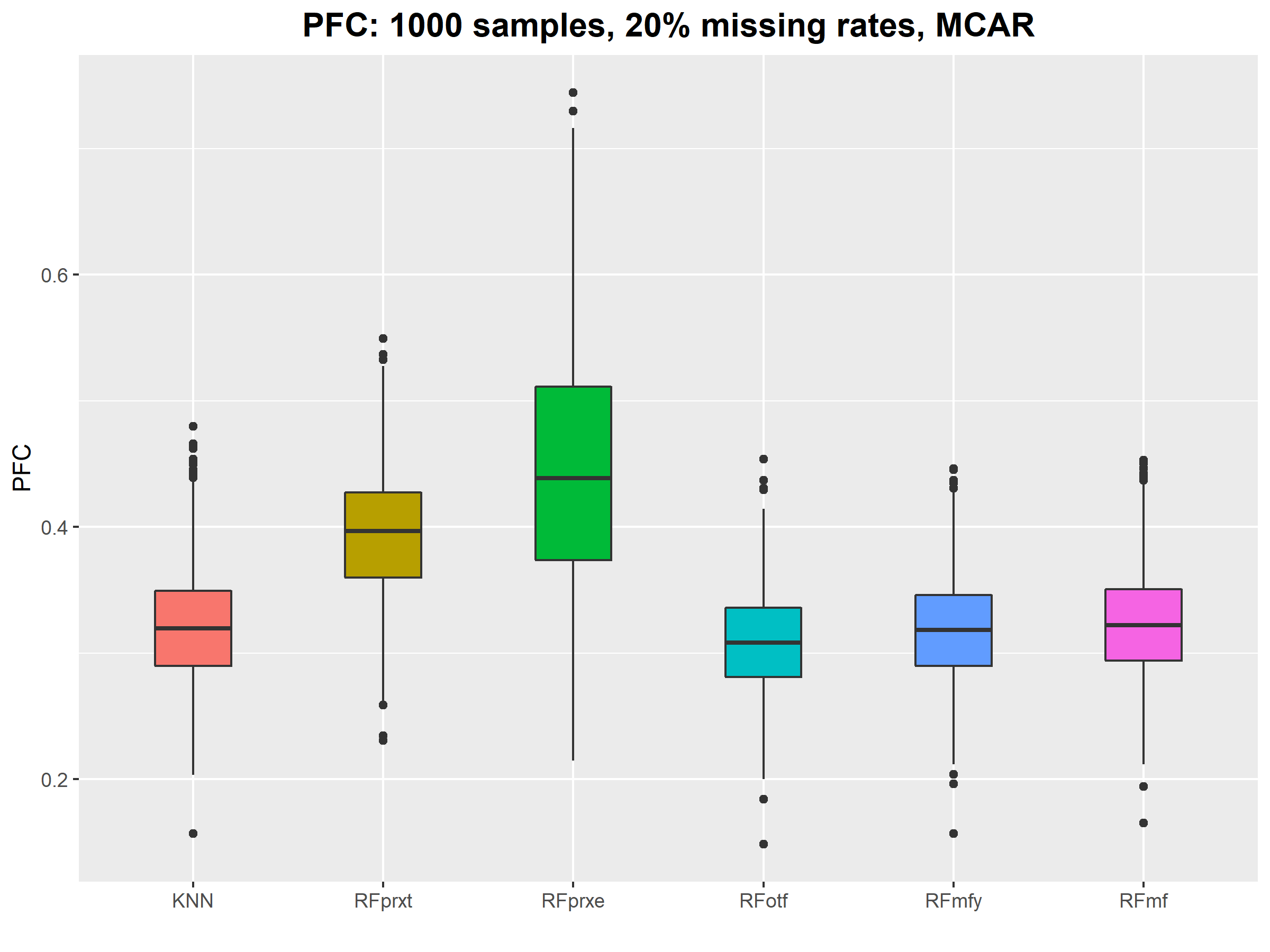

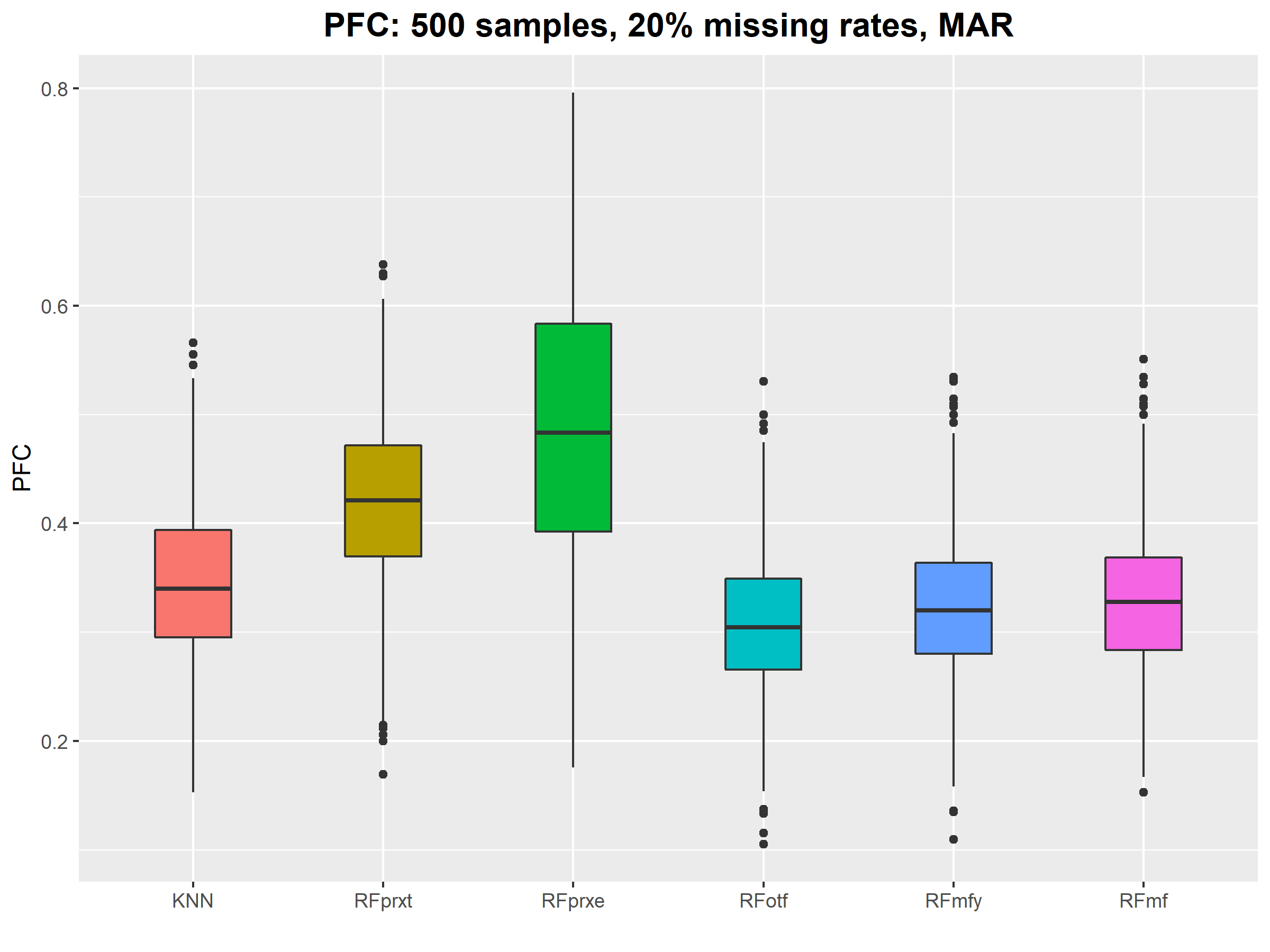

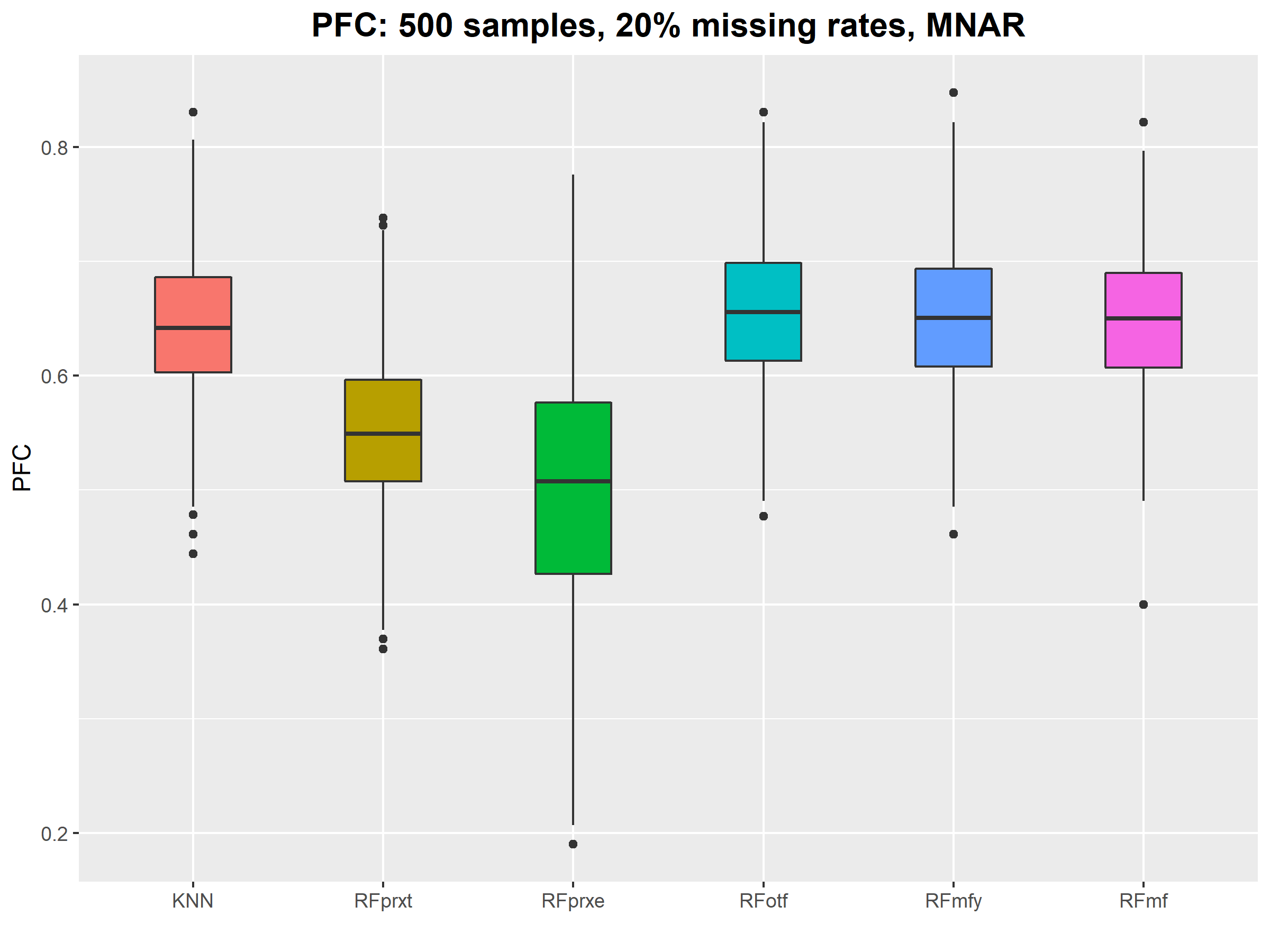

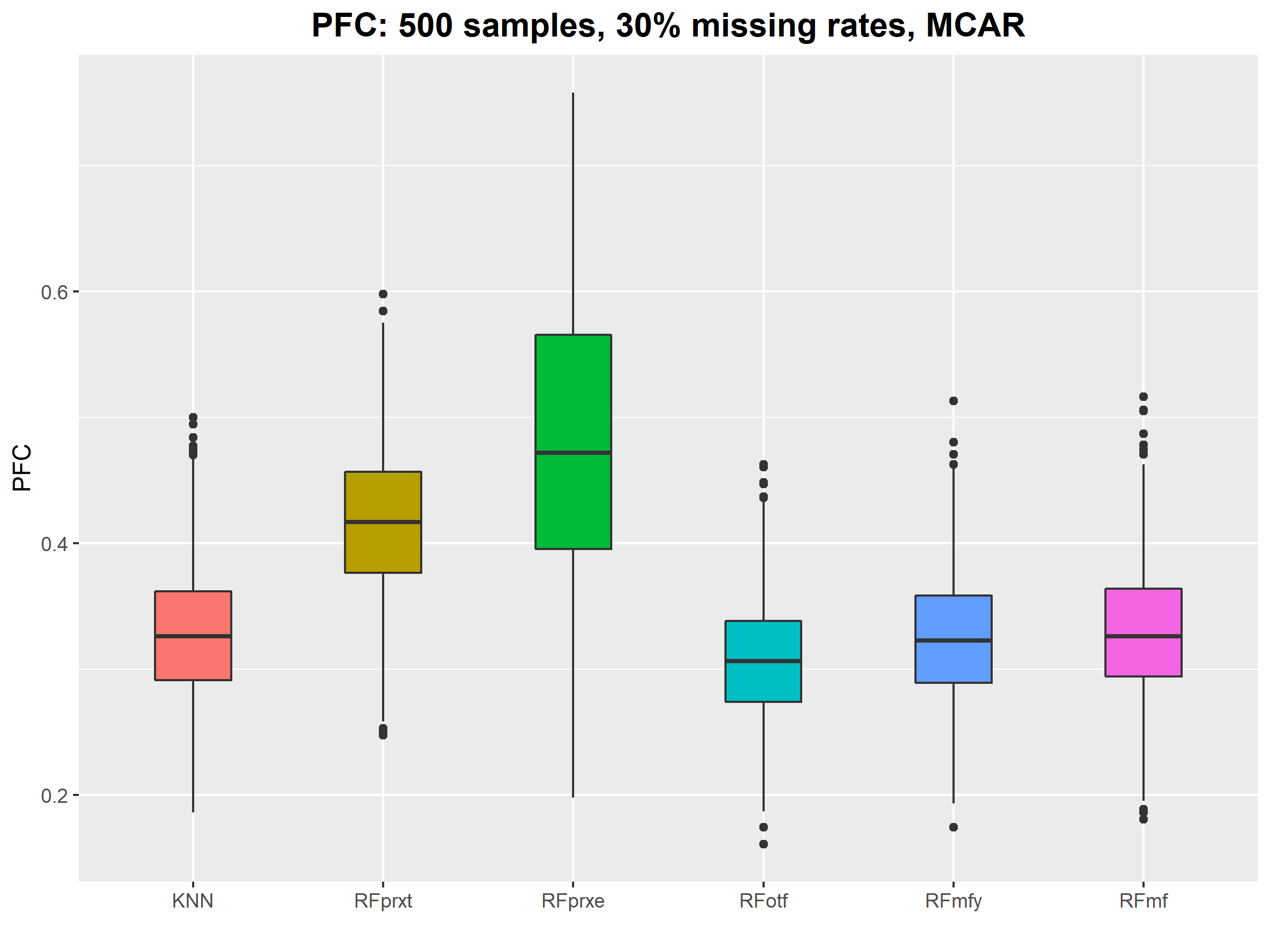

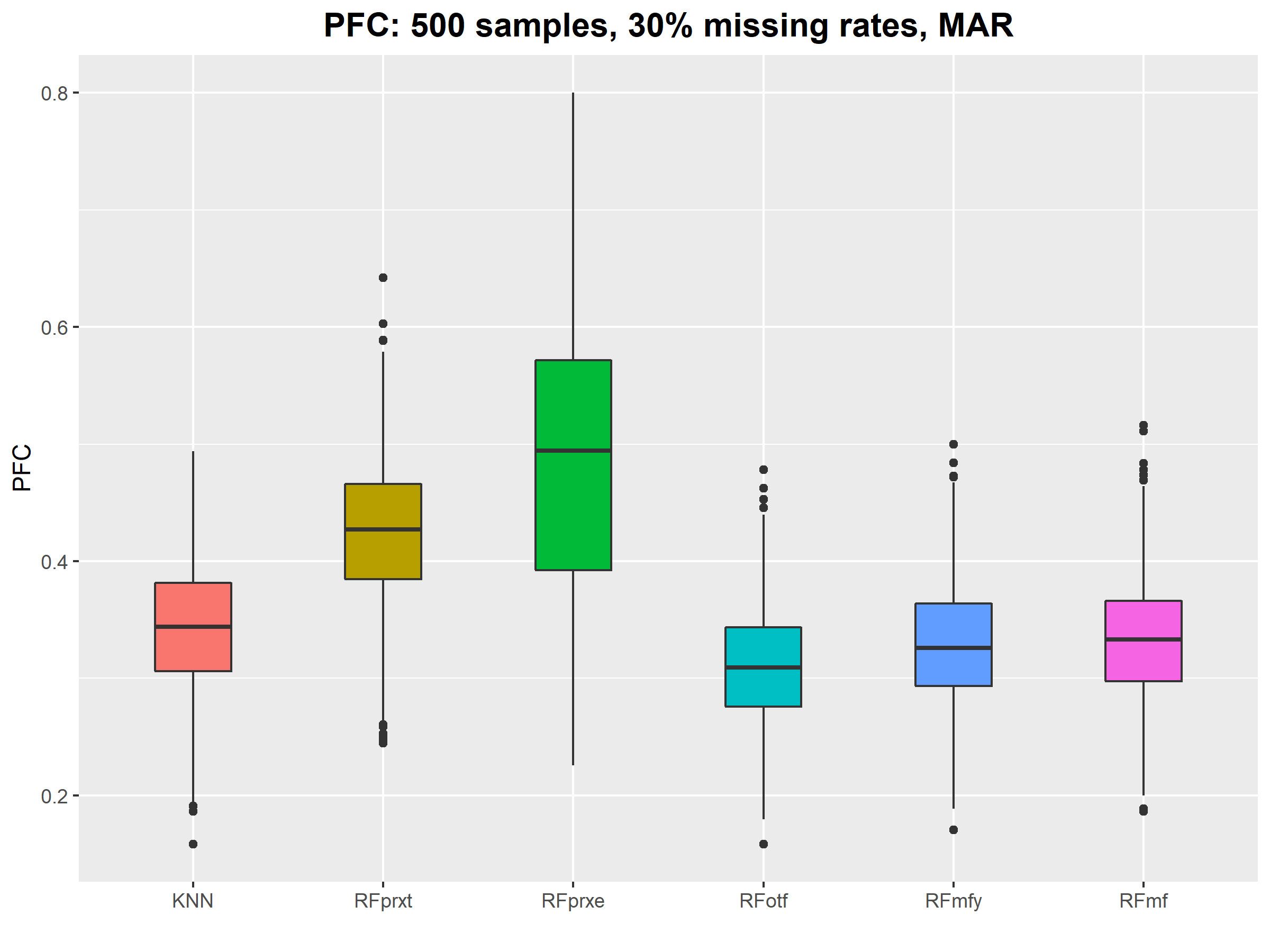

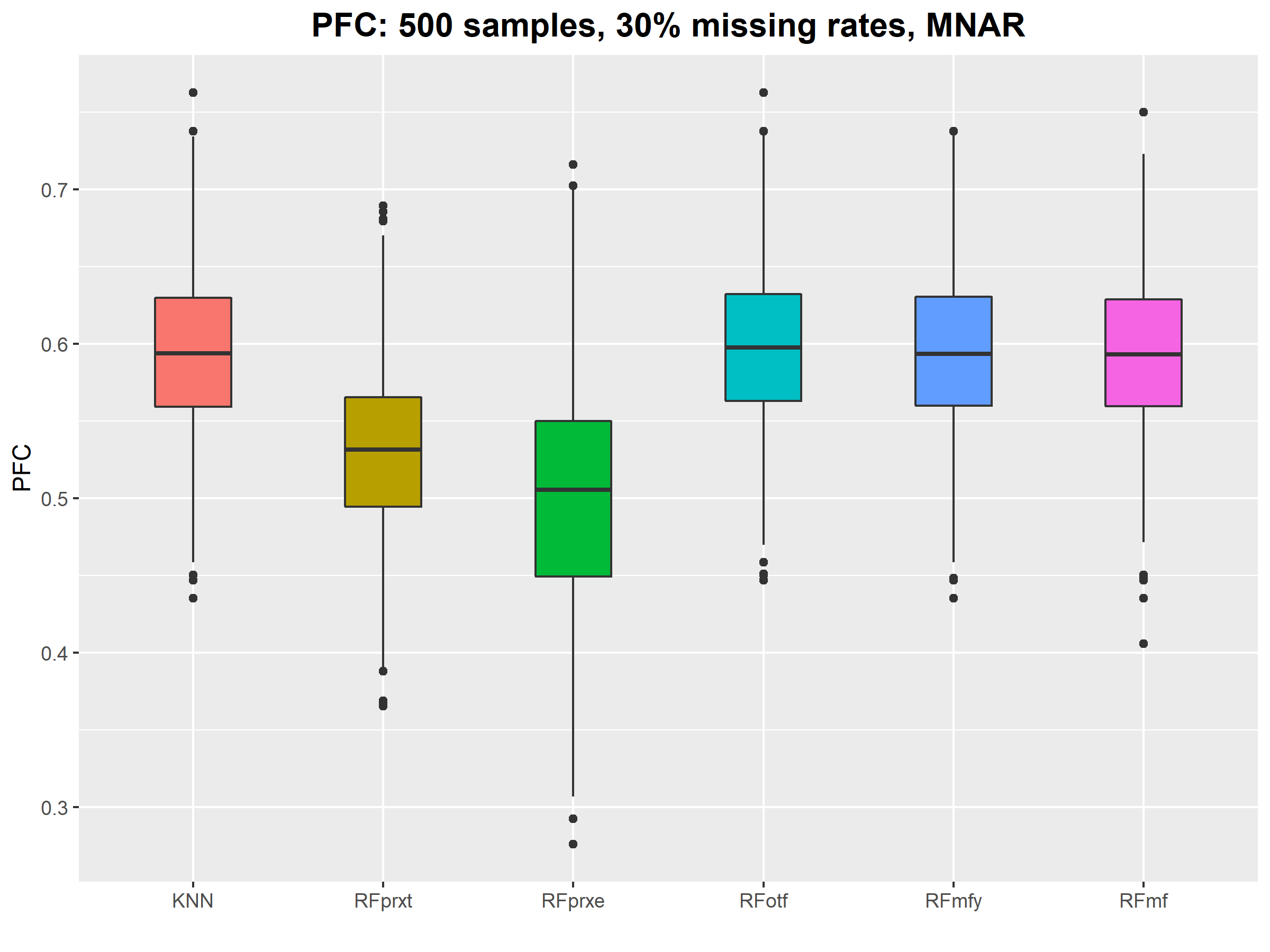


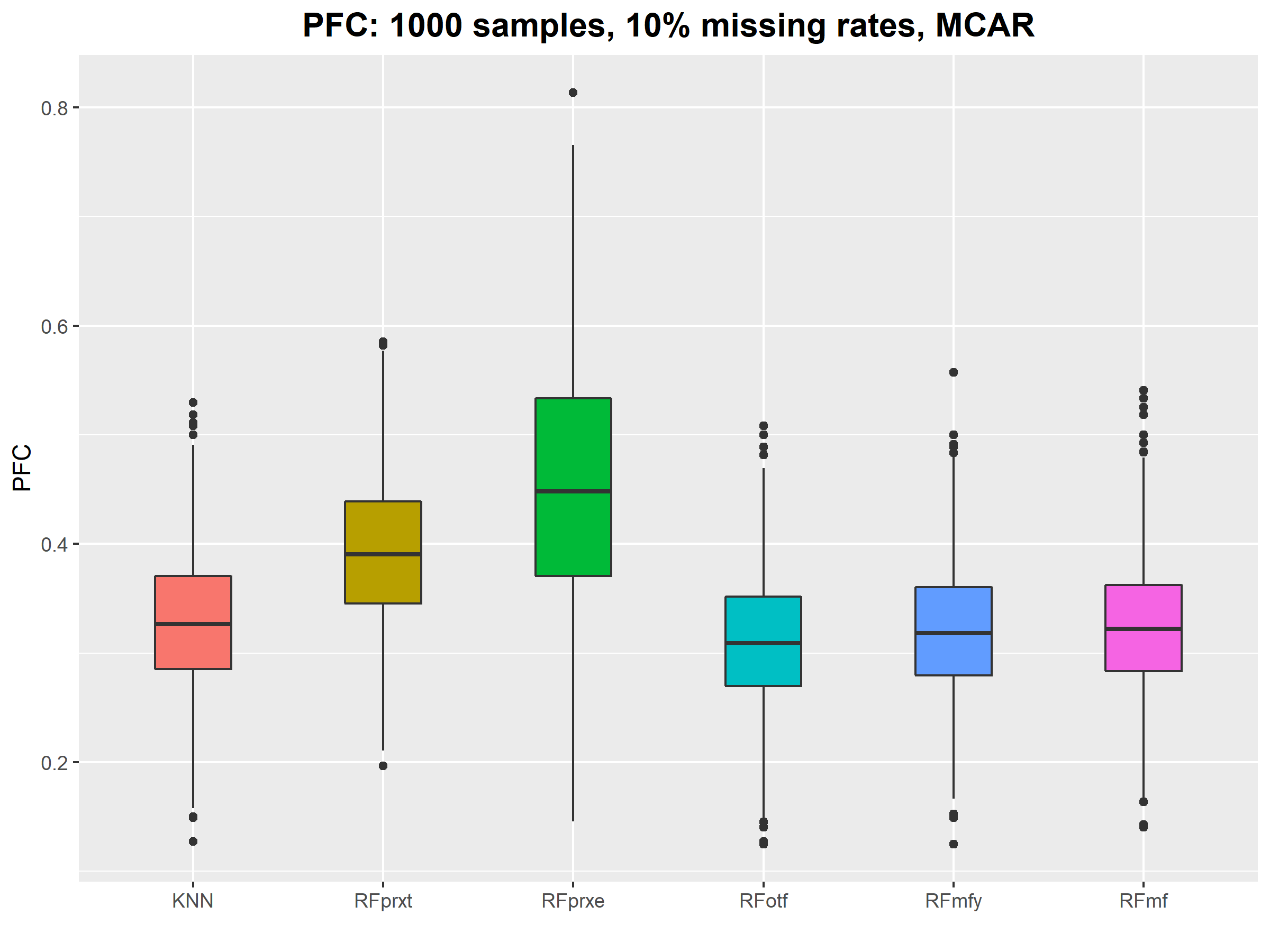


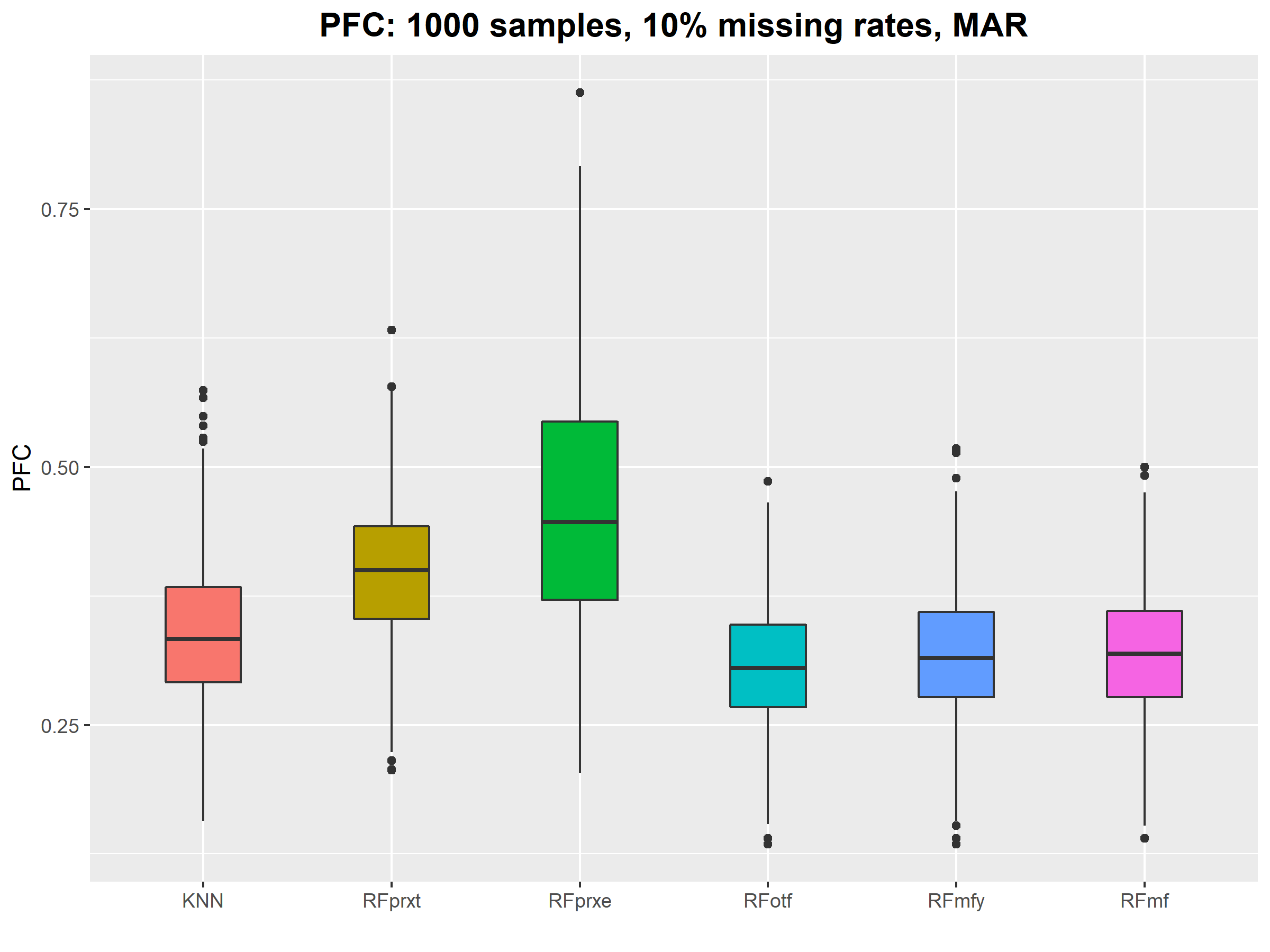

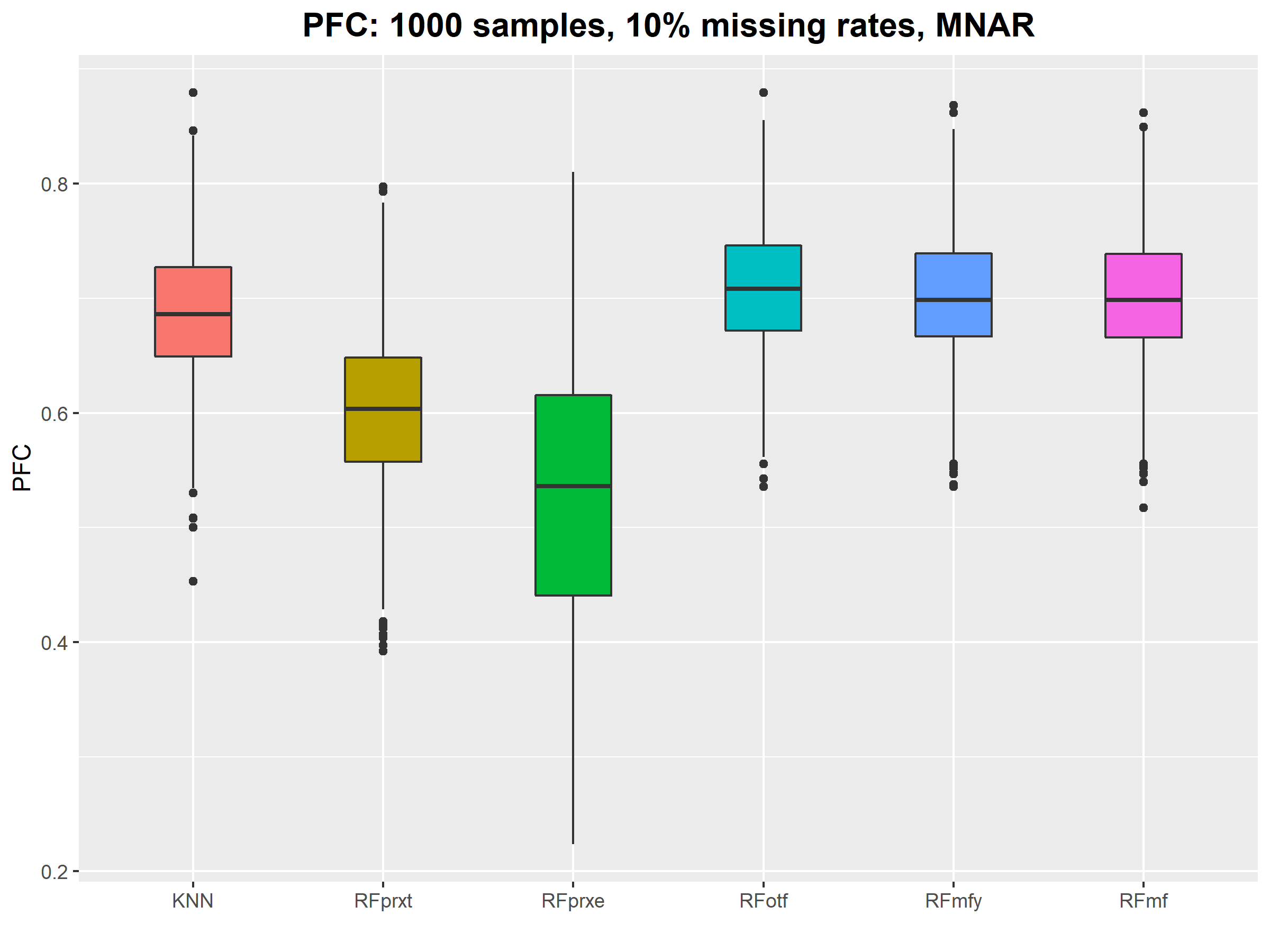

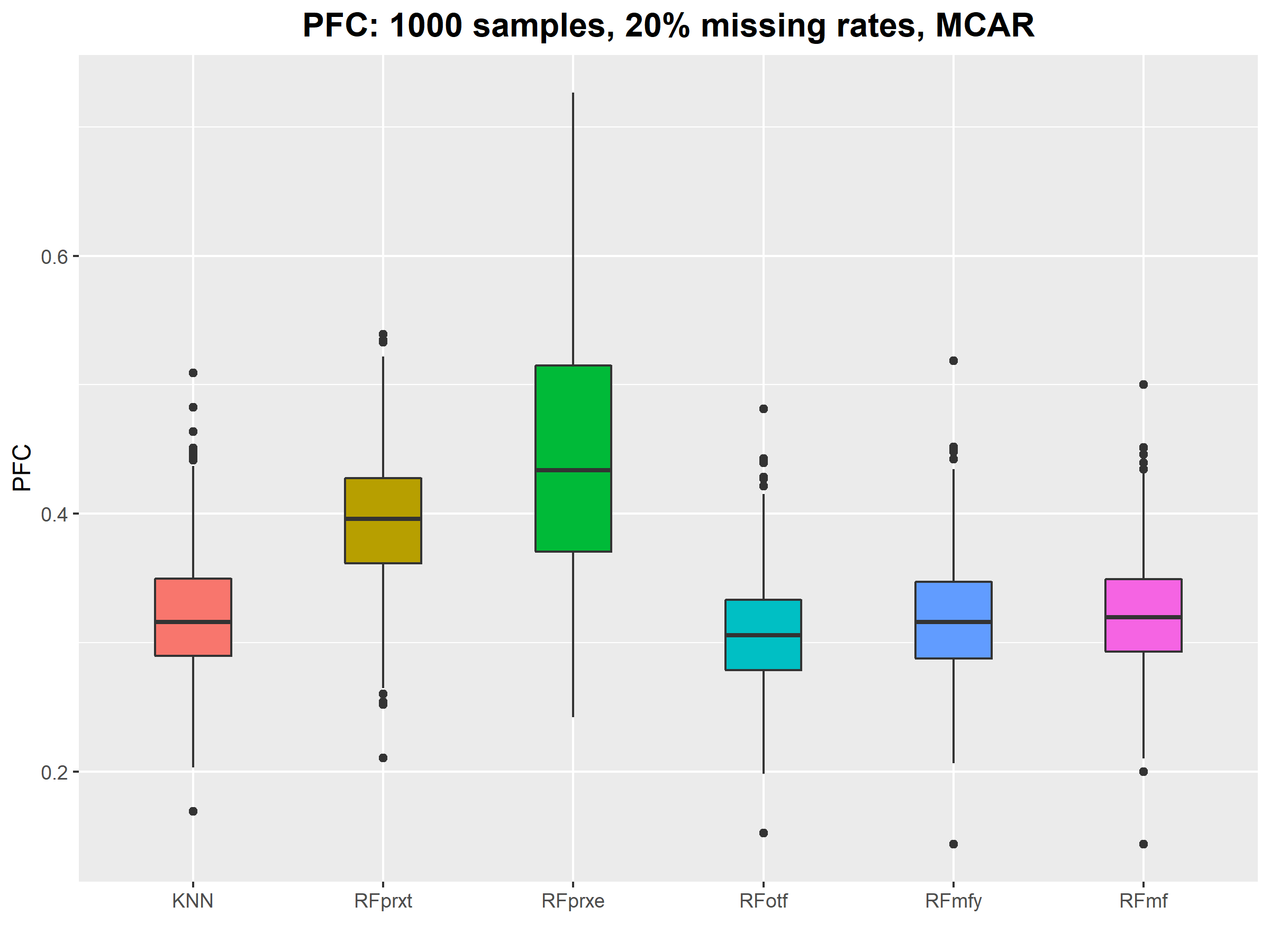

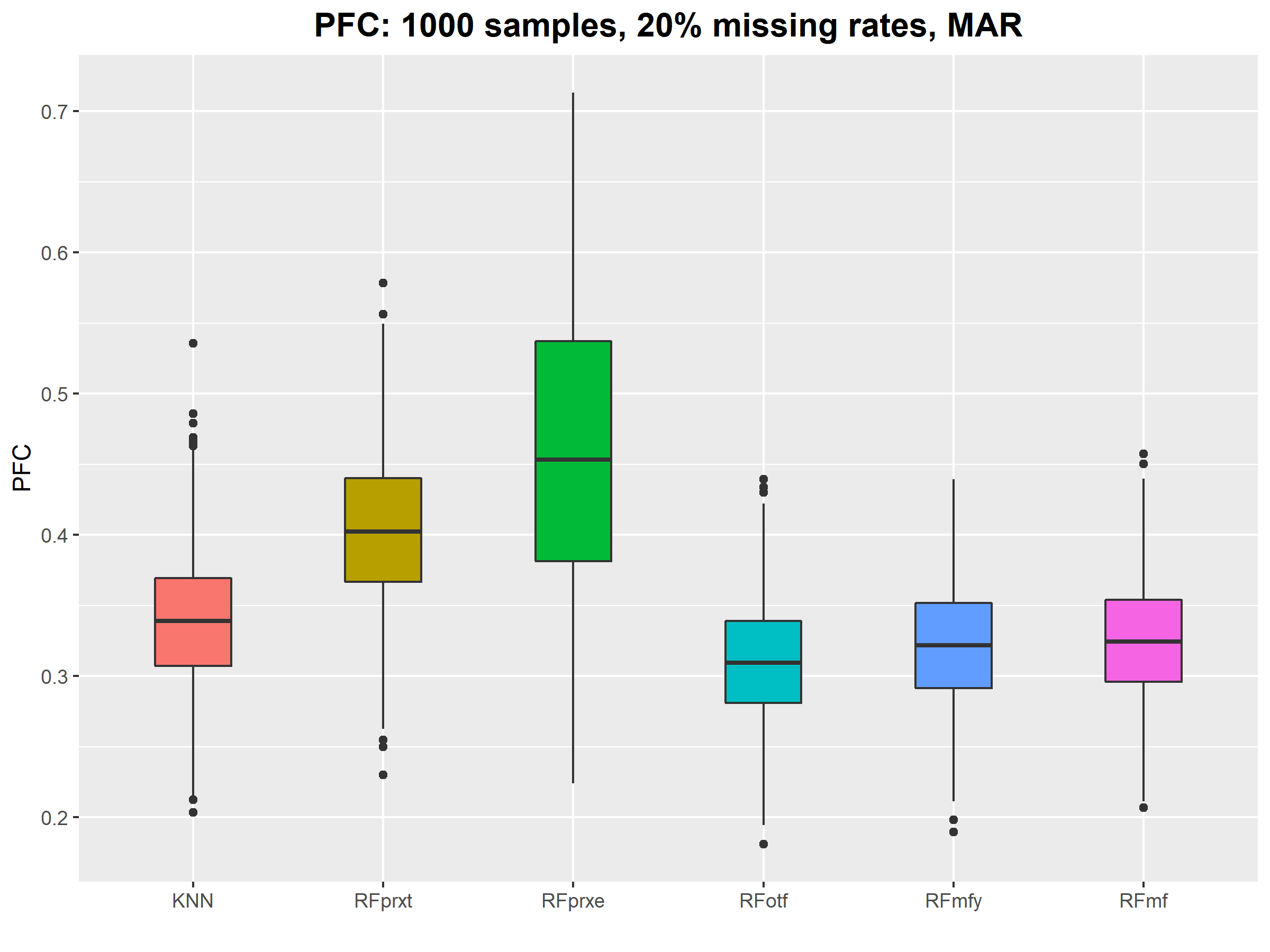

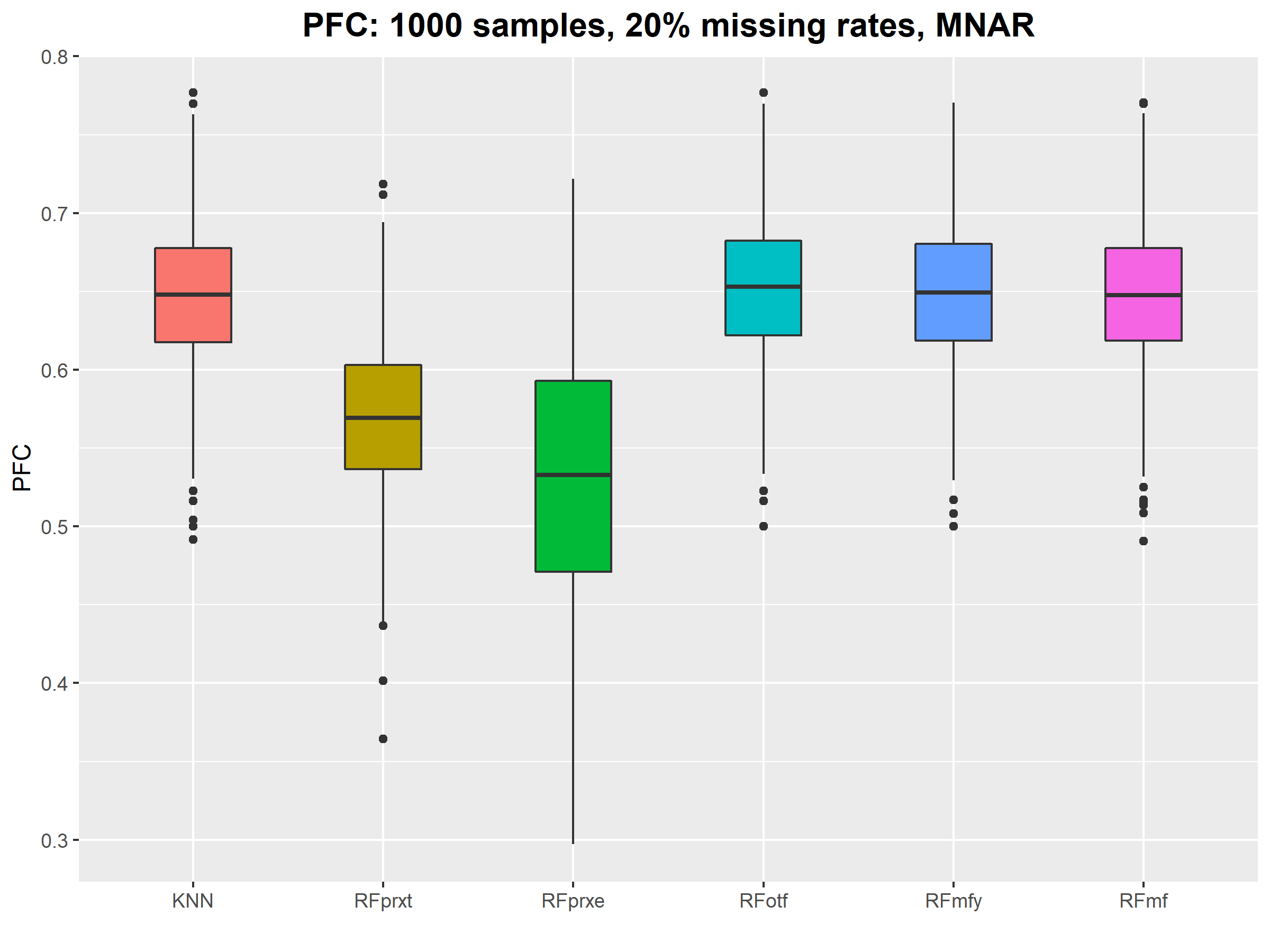

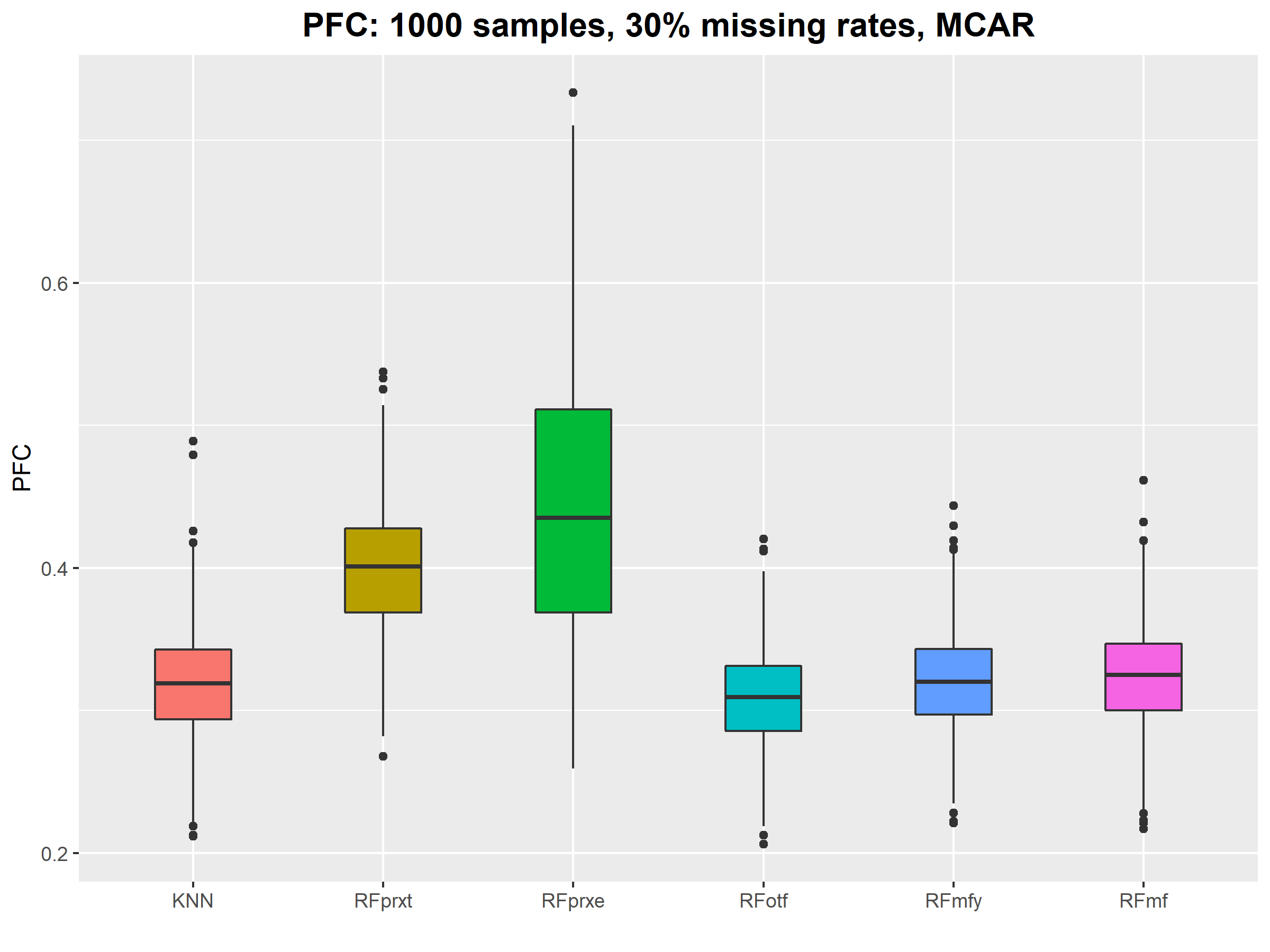

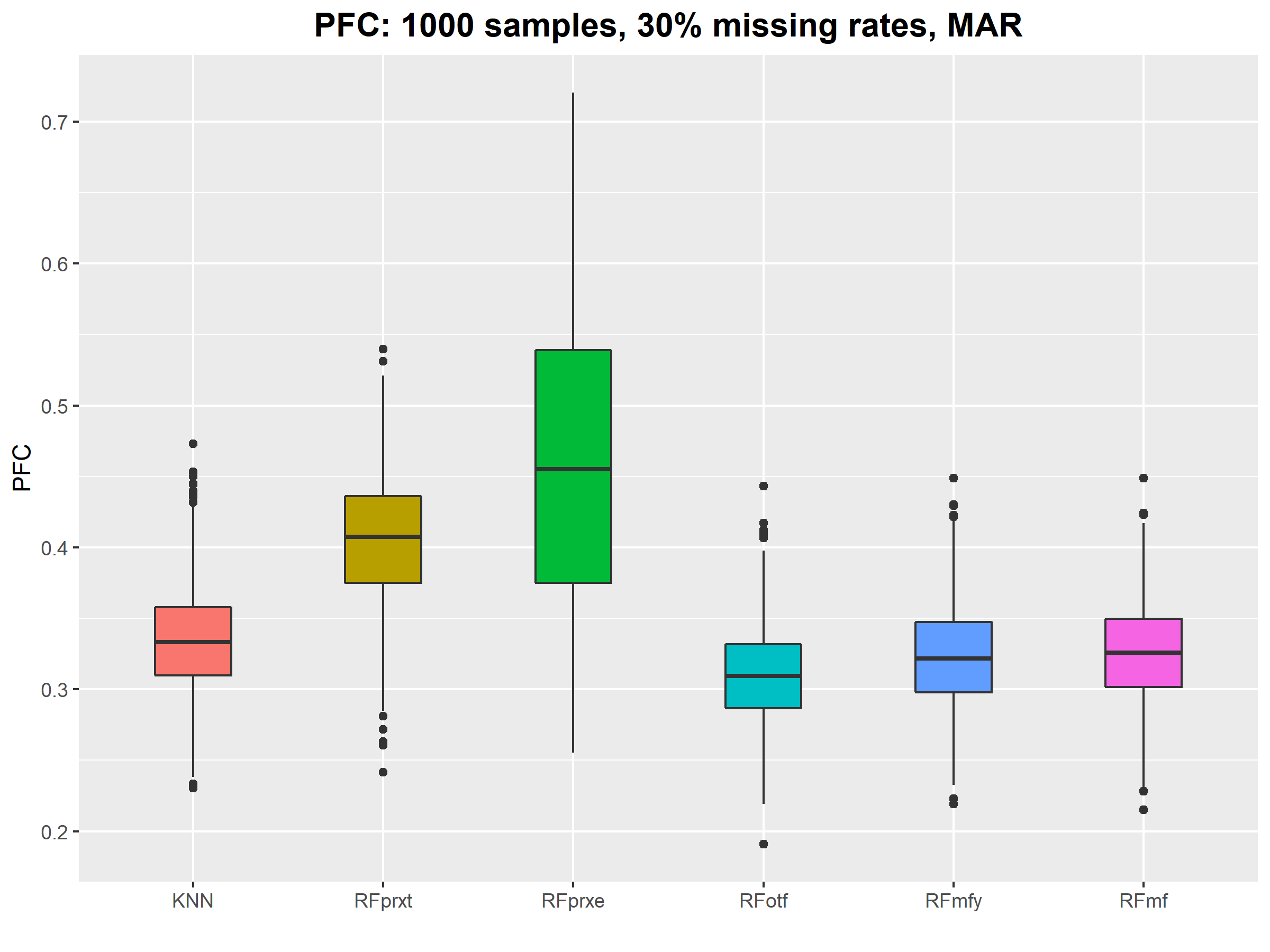

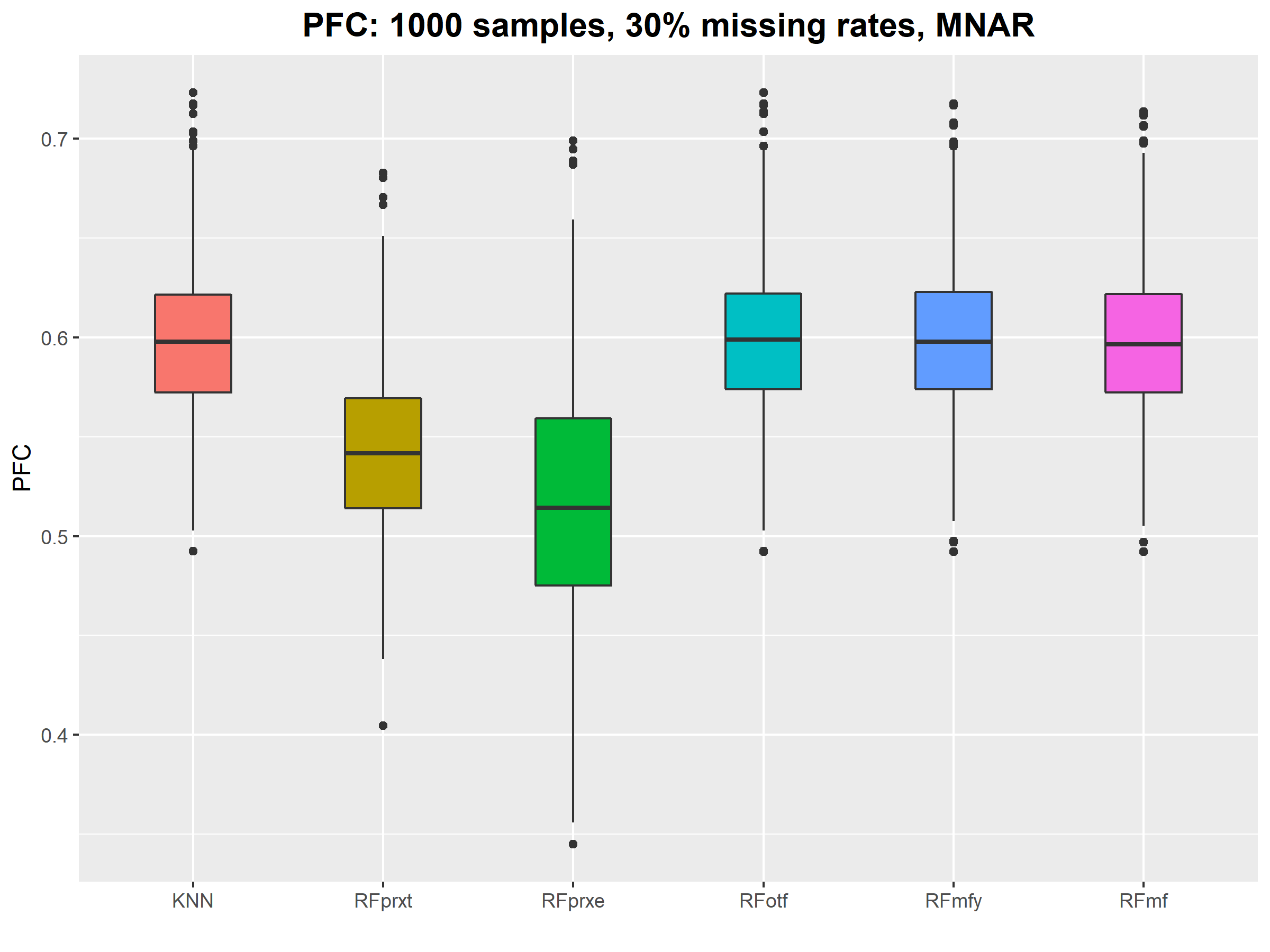


Figure S2: RMSE with different sample sizes and missing rates


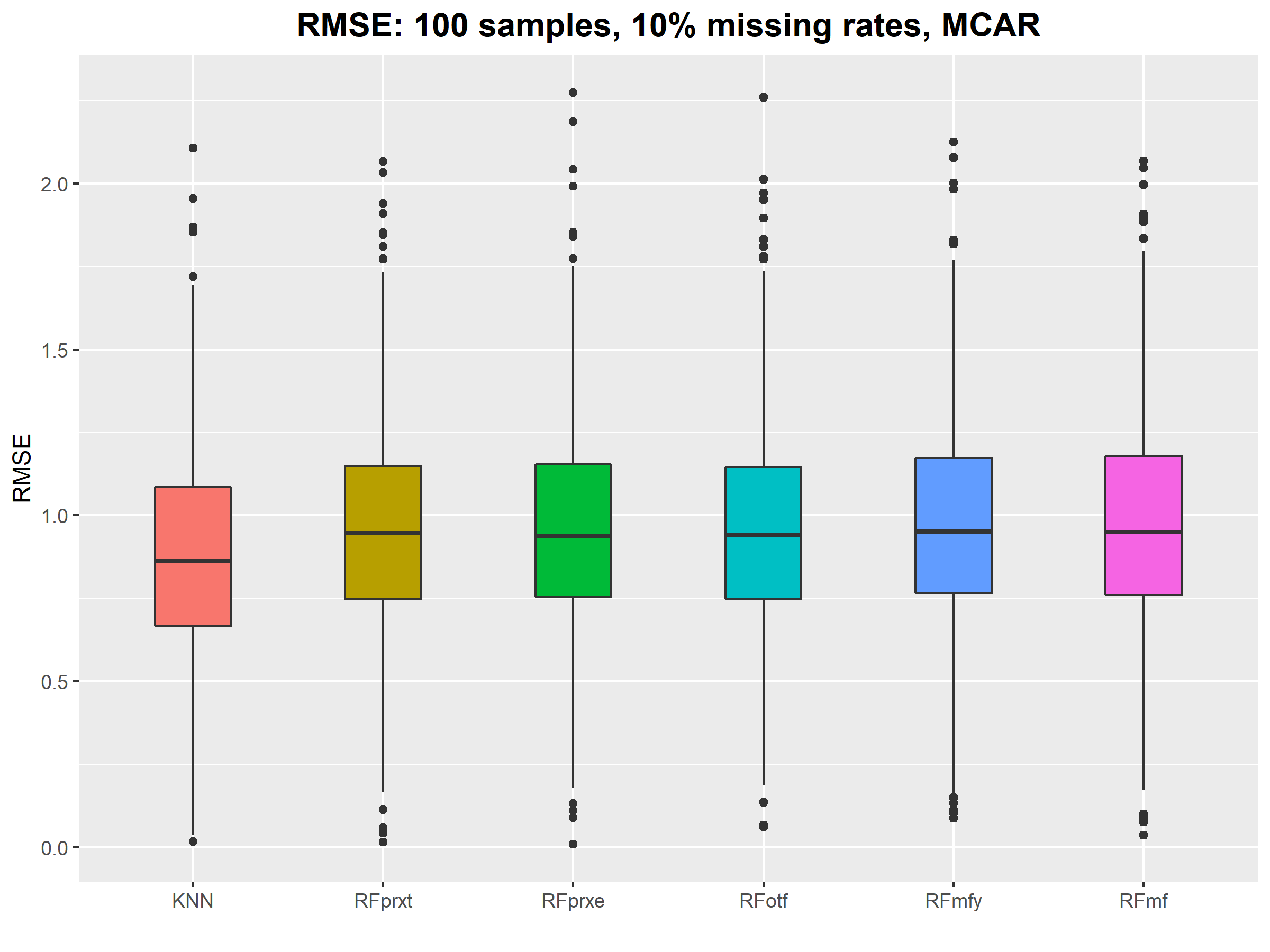

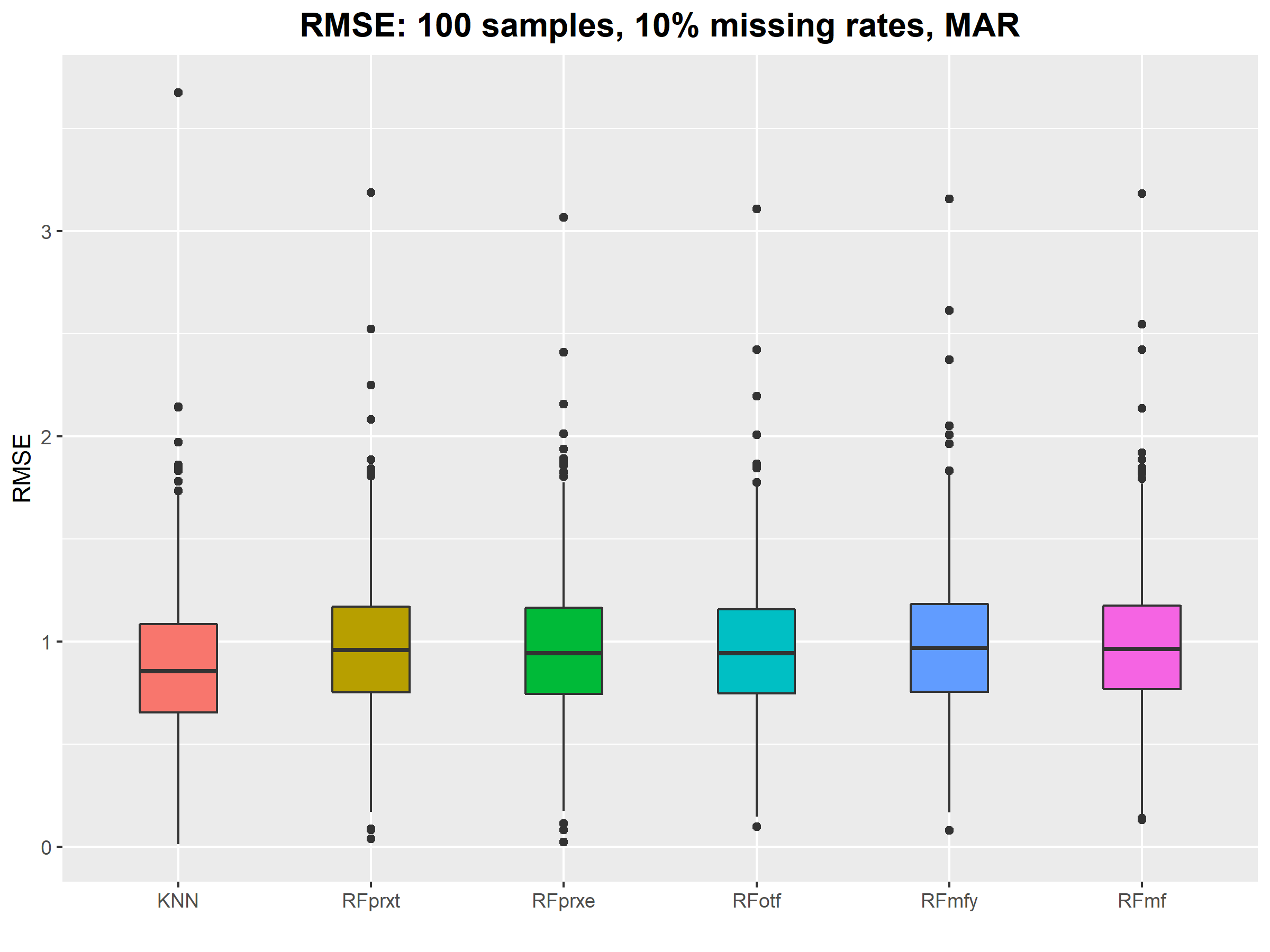

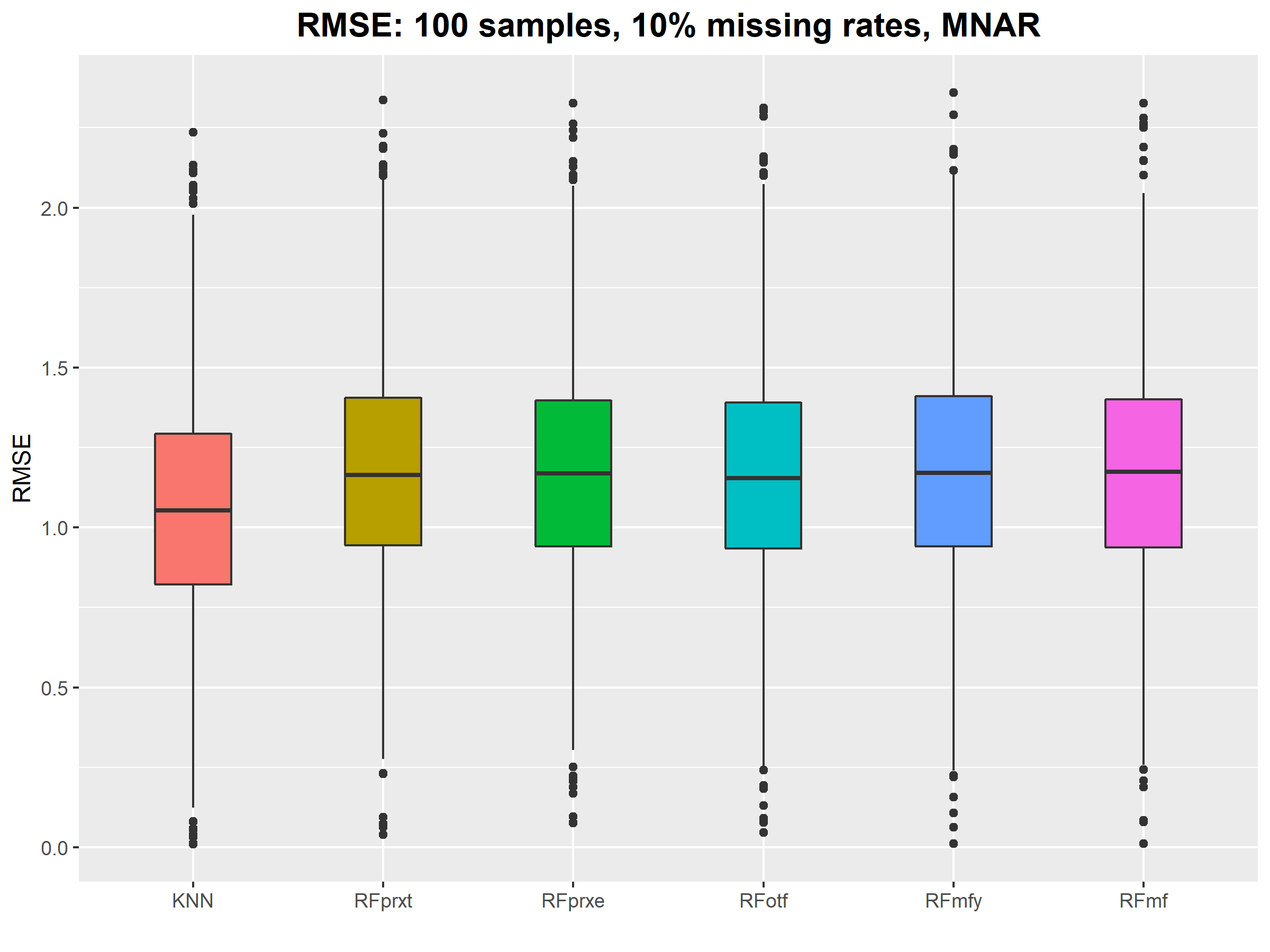

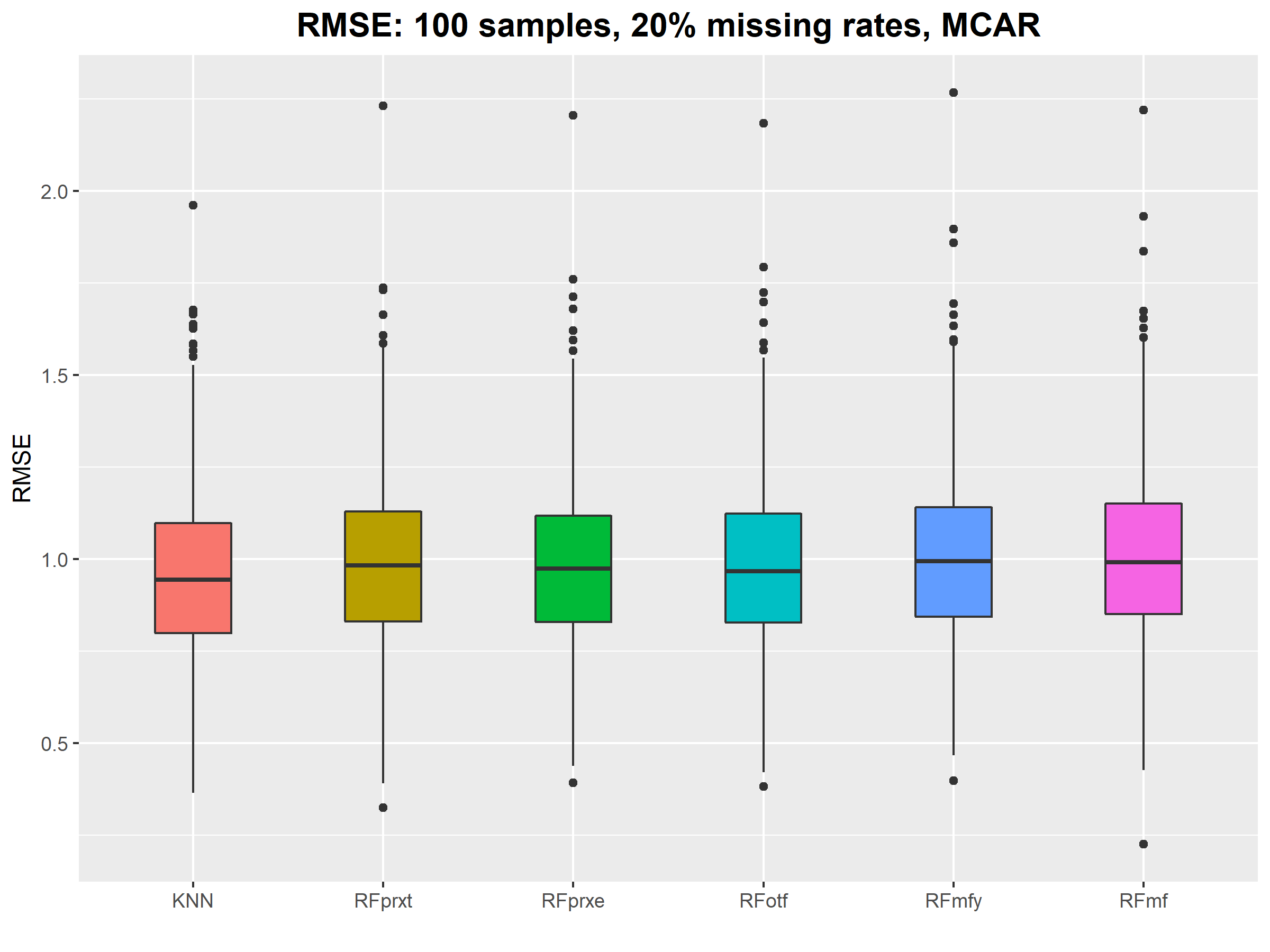

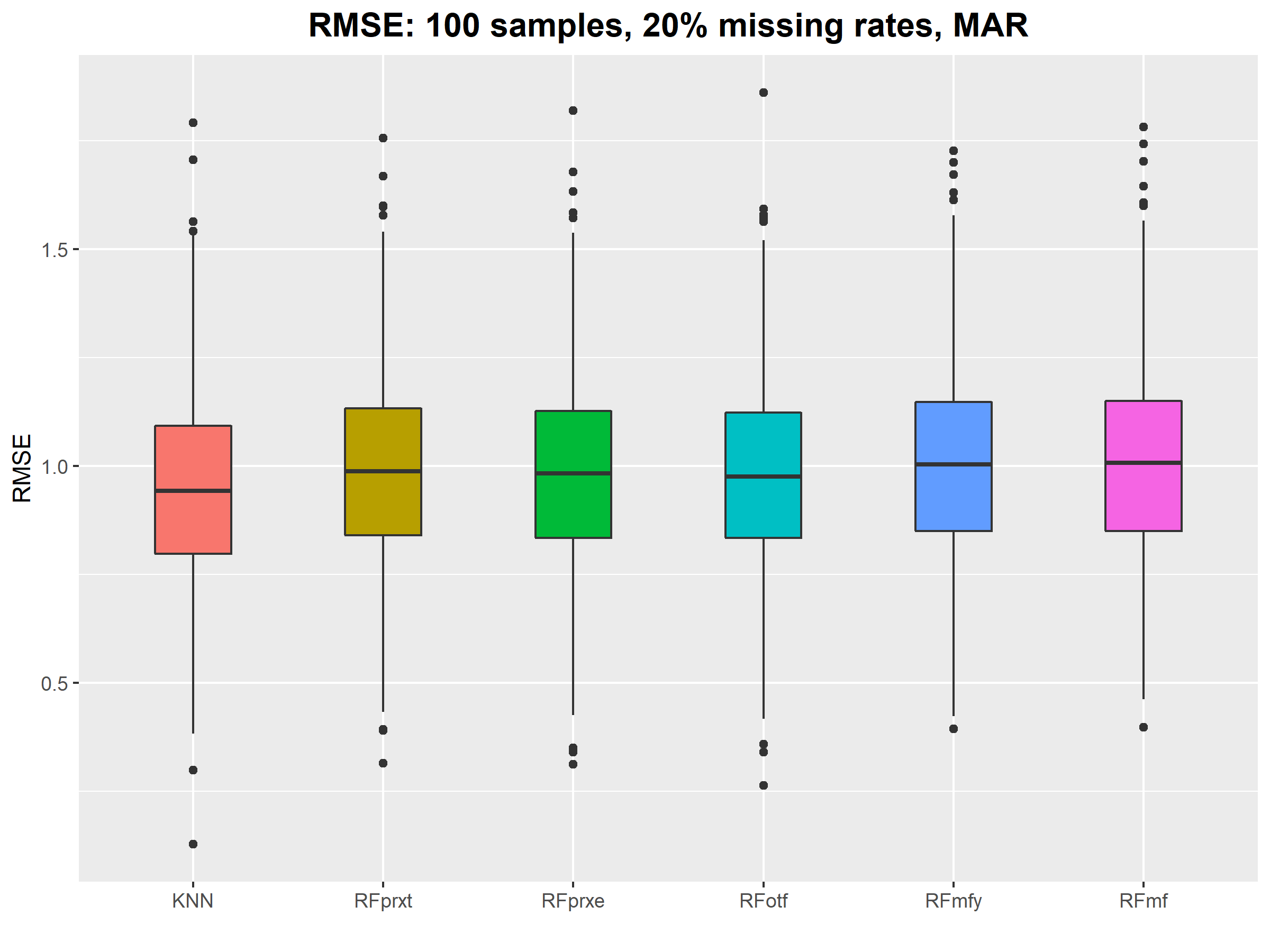

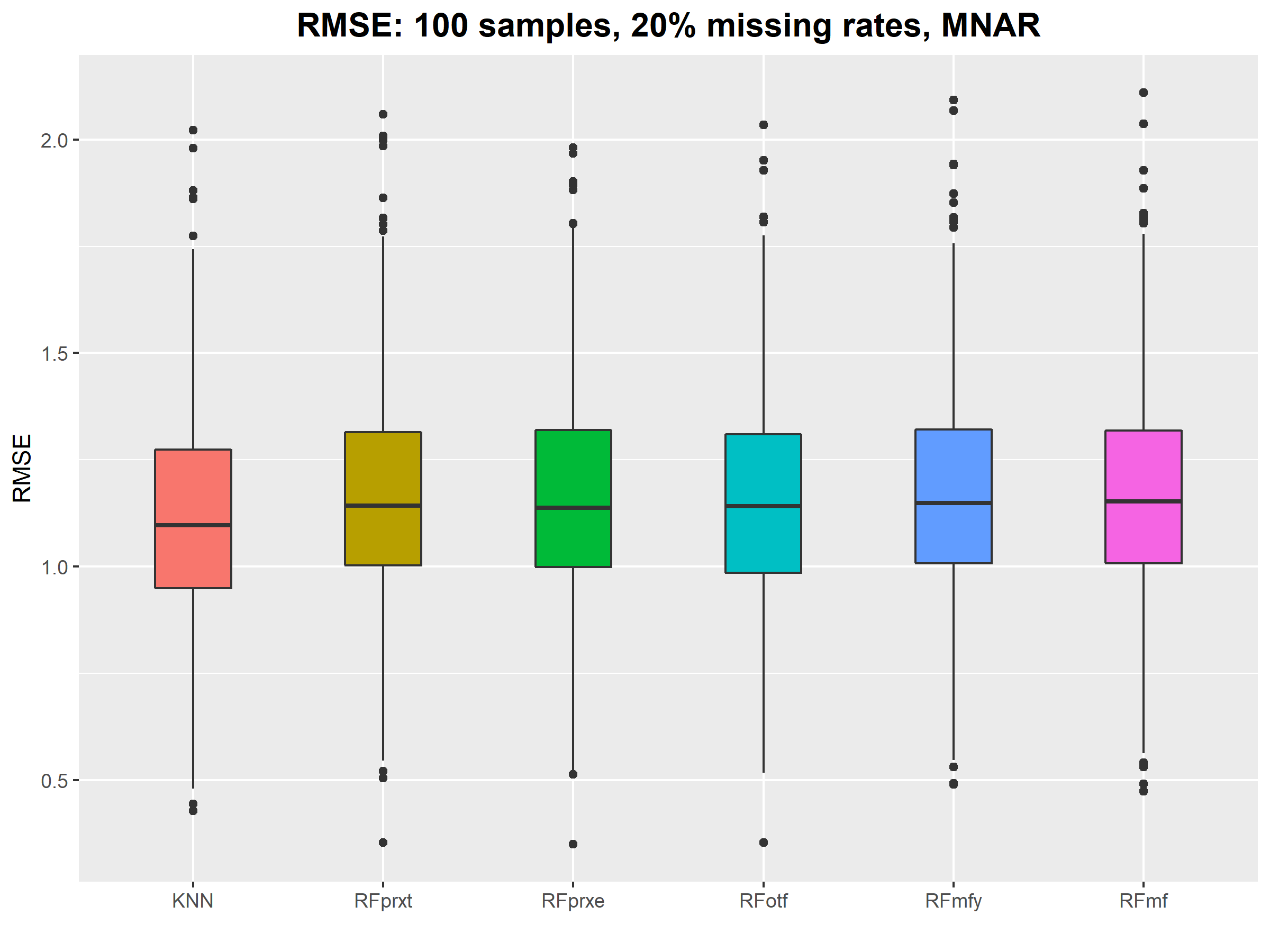

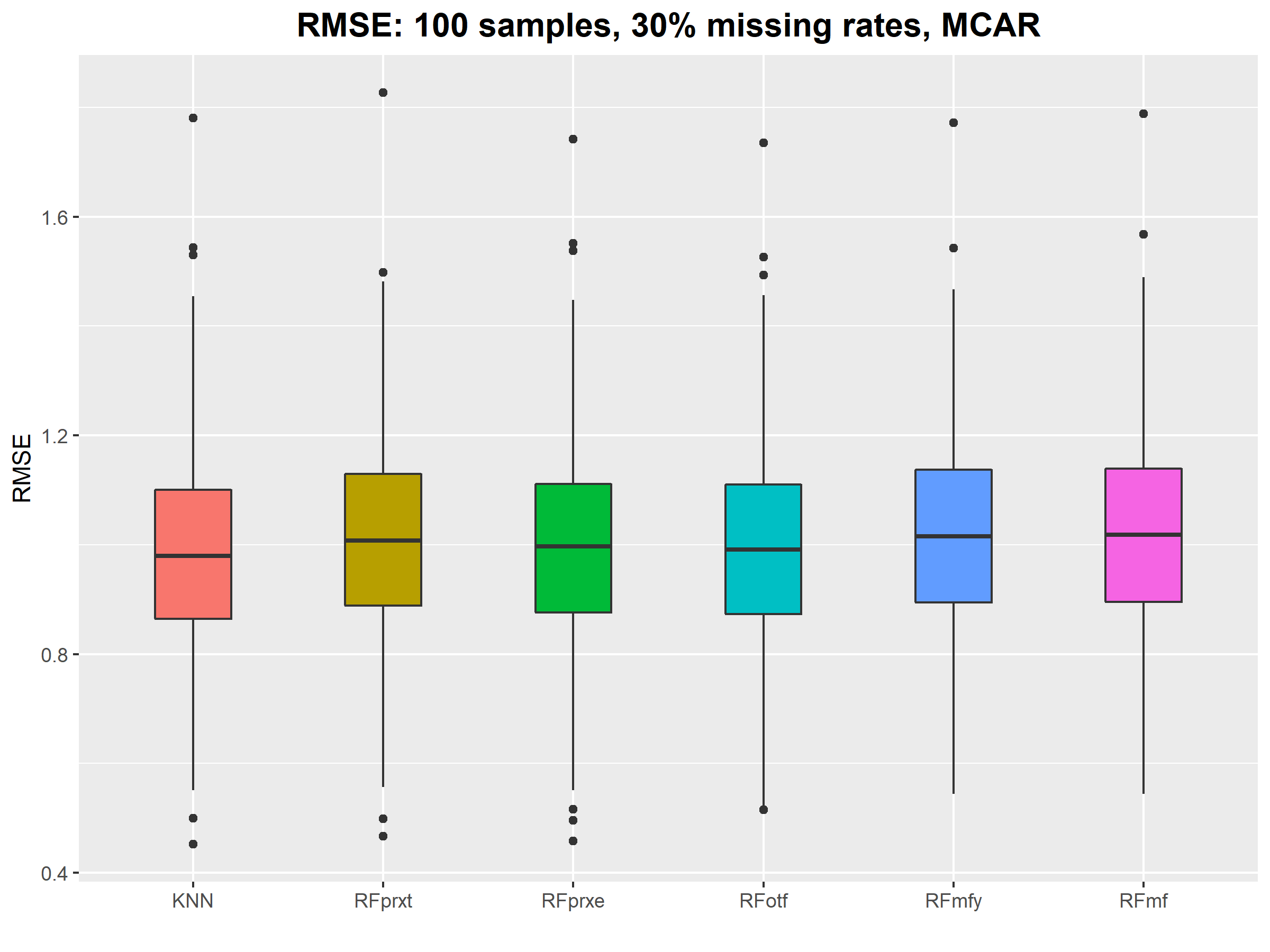

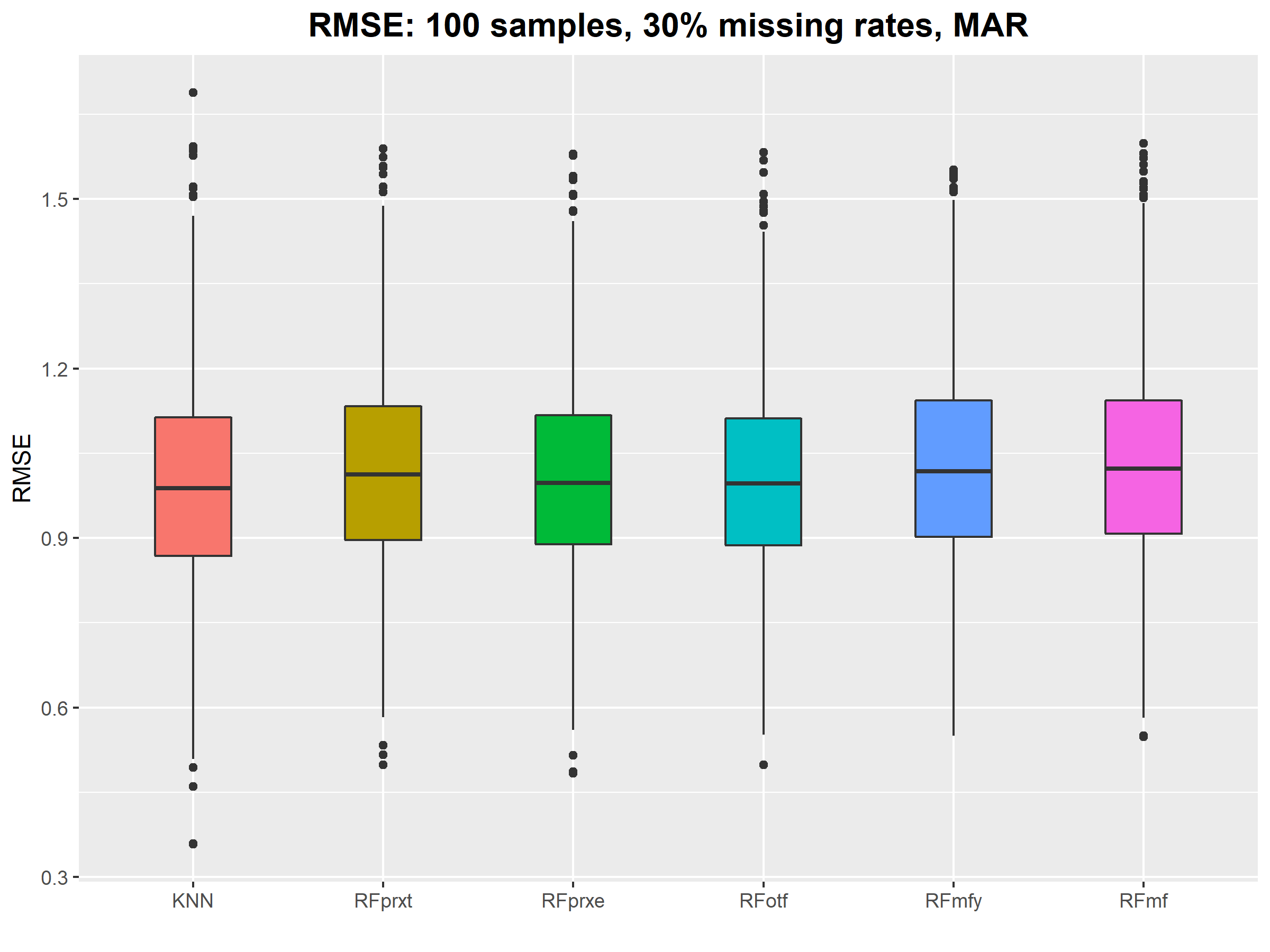

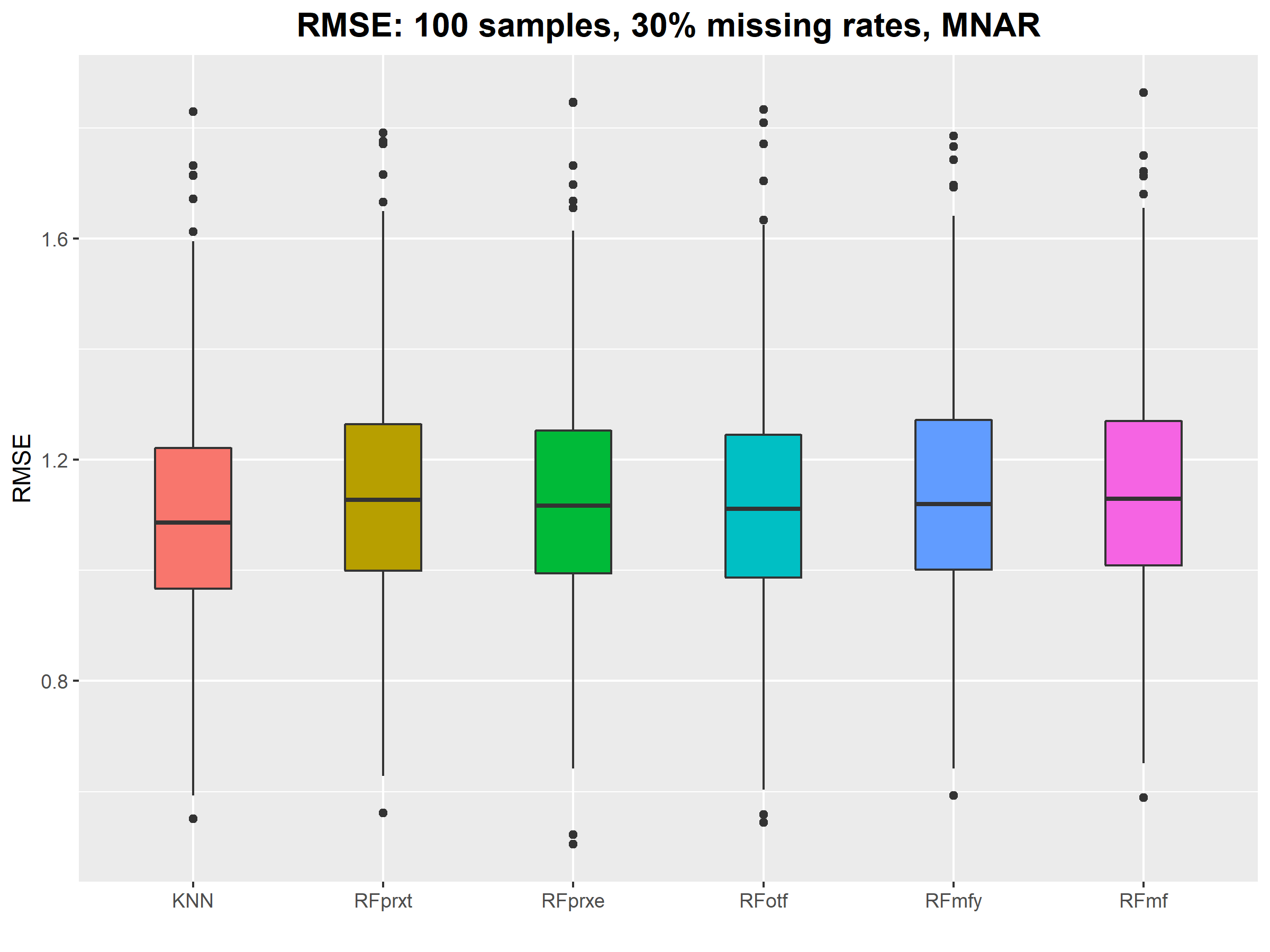


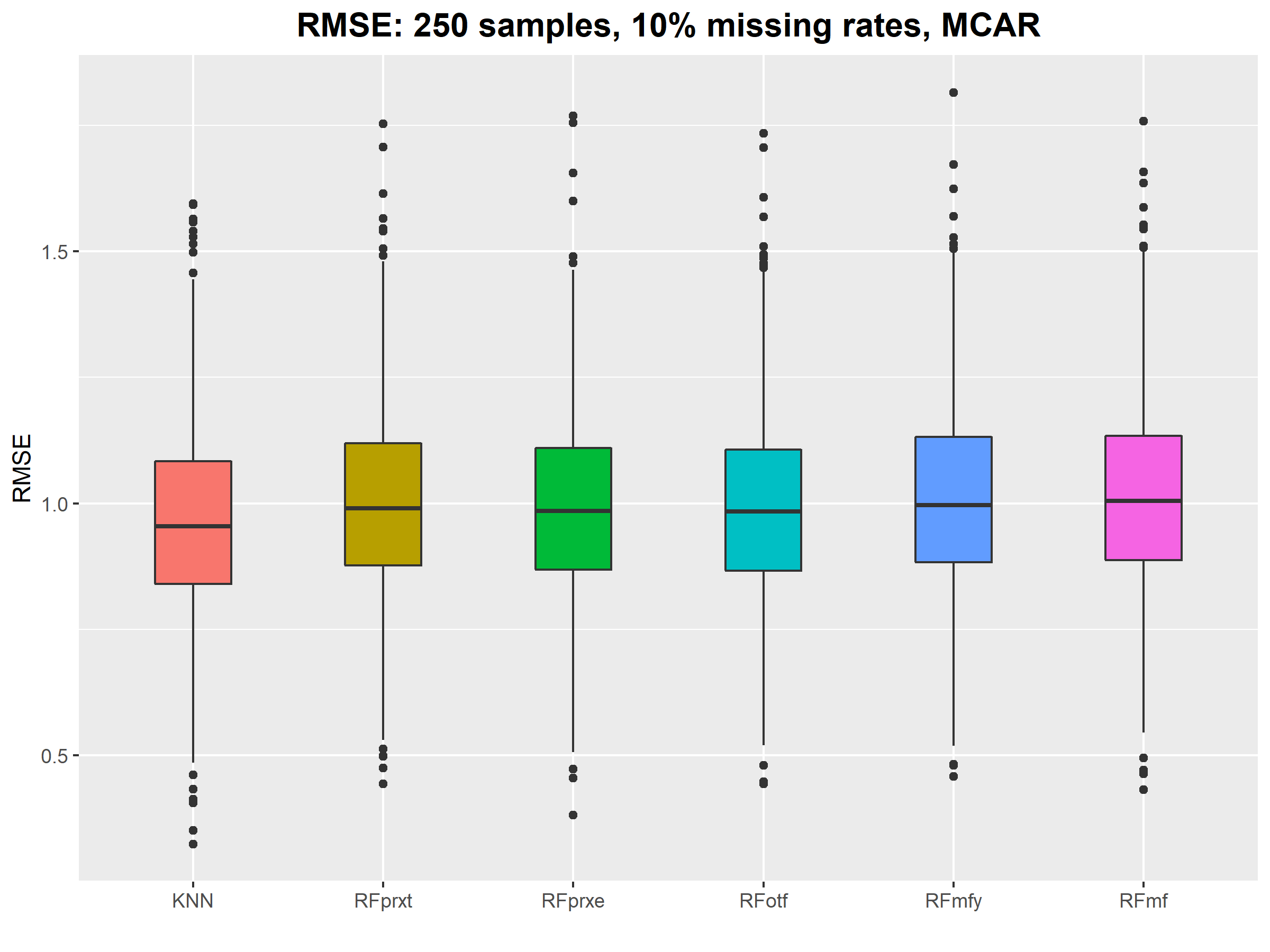

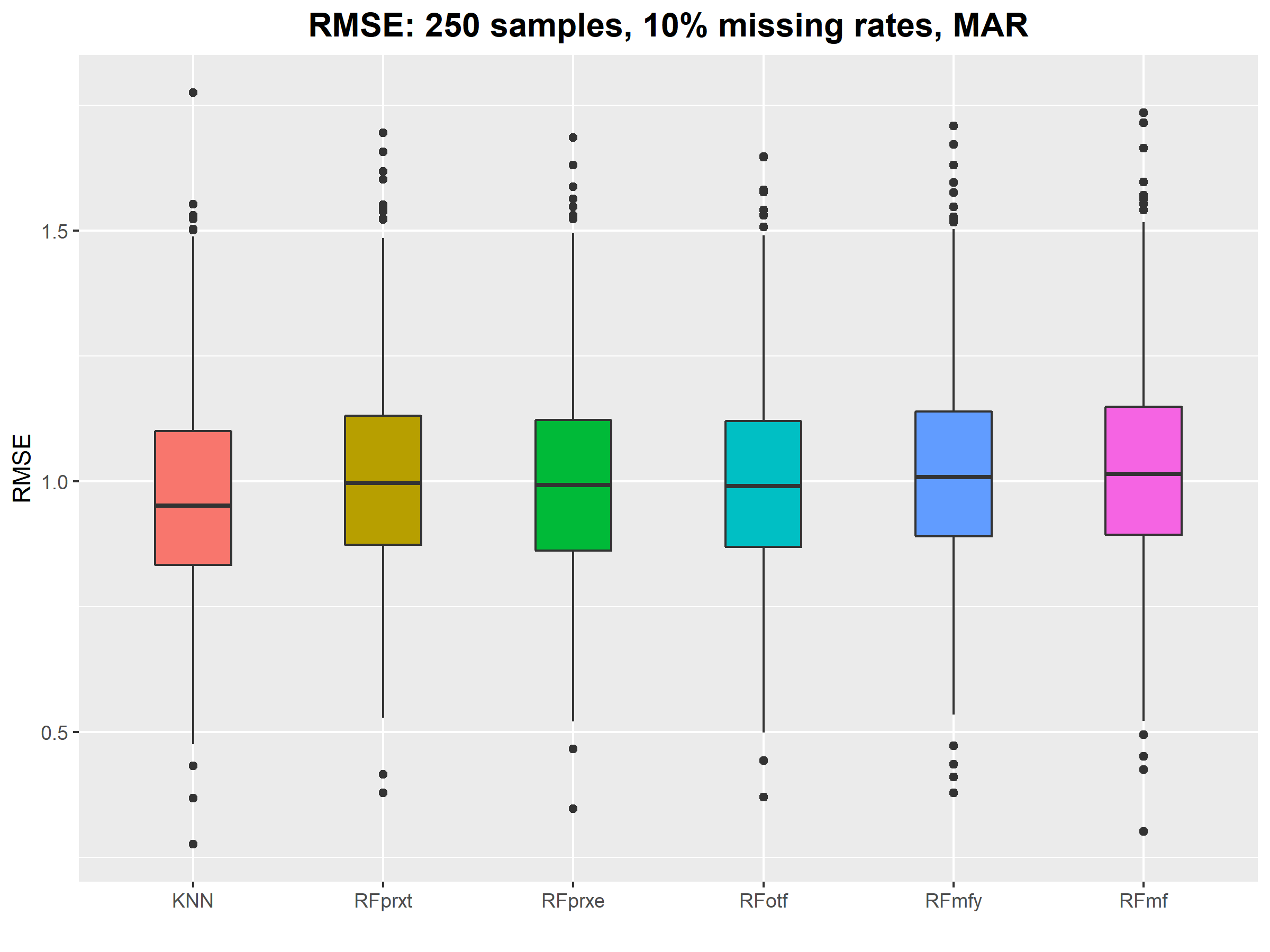

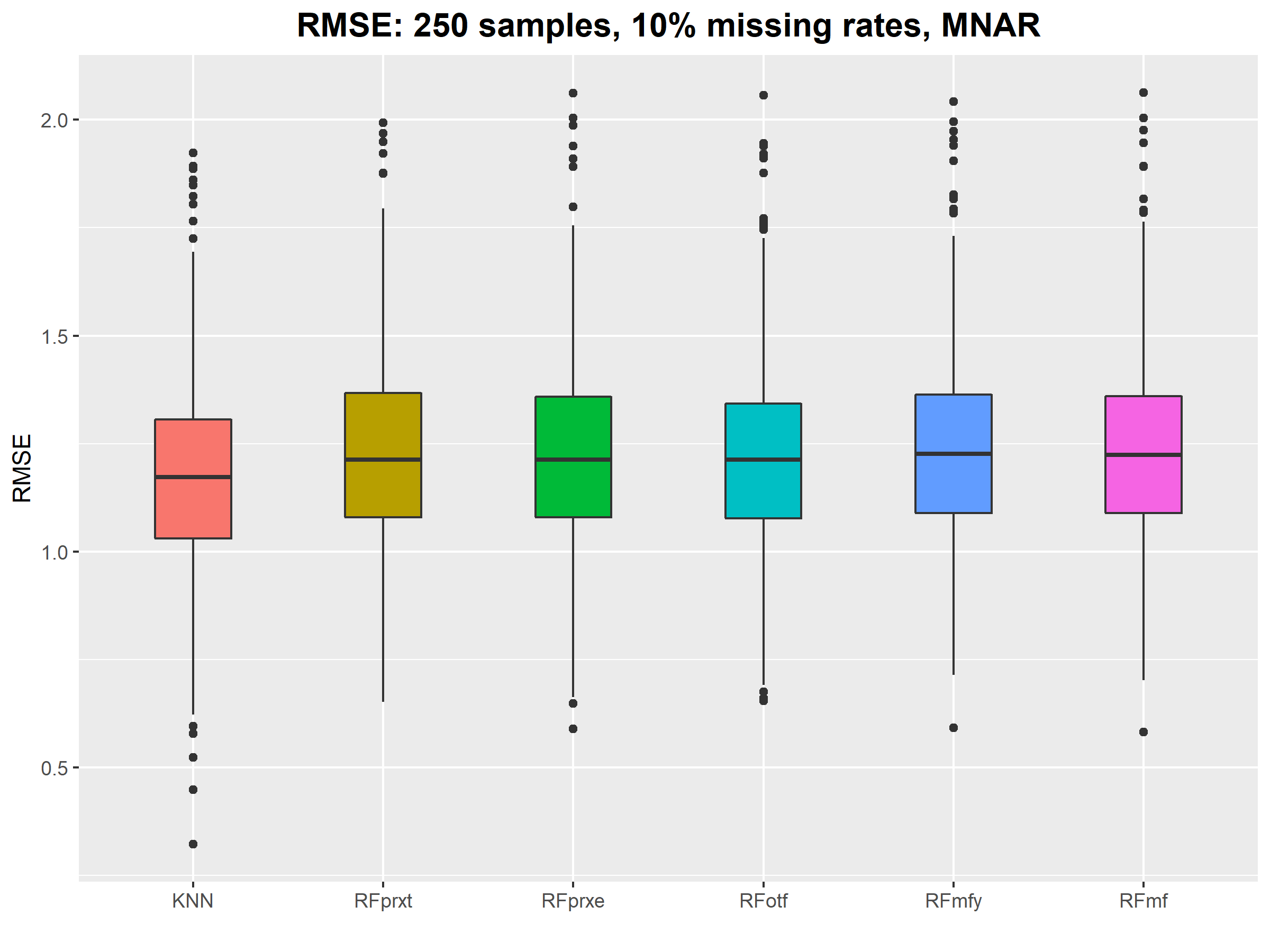

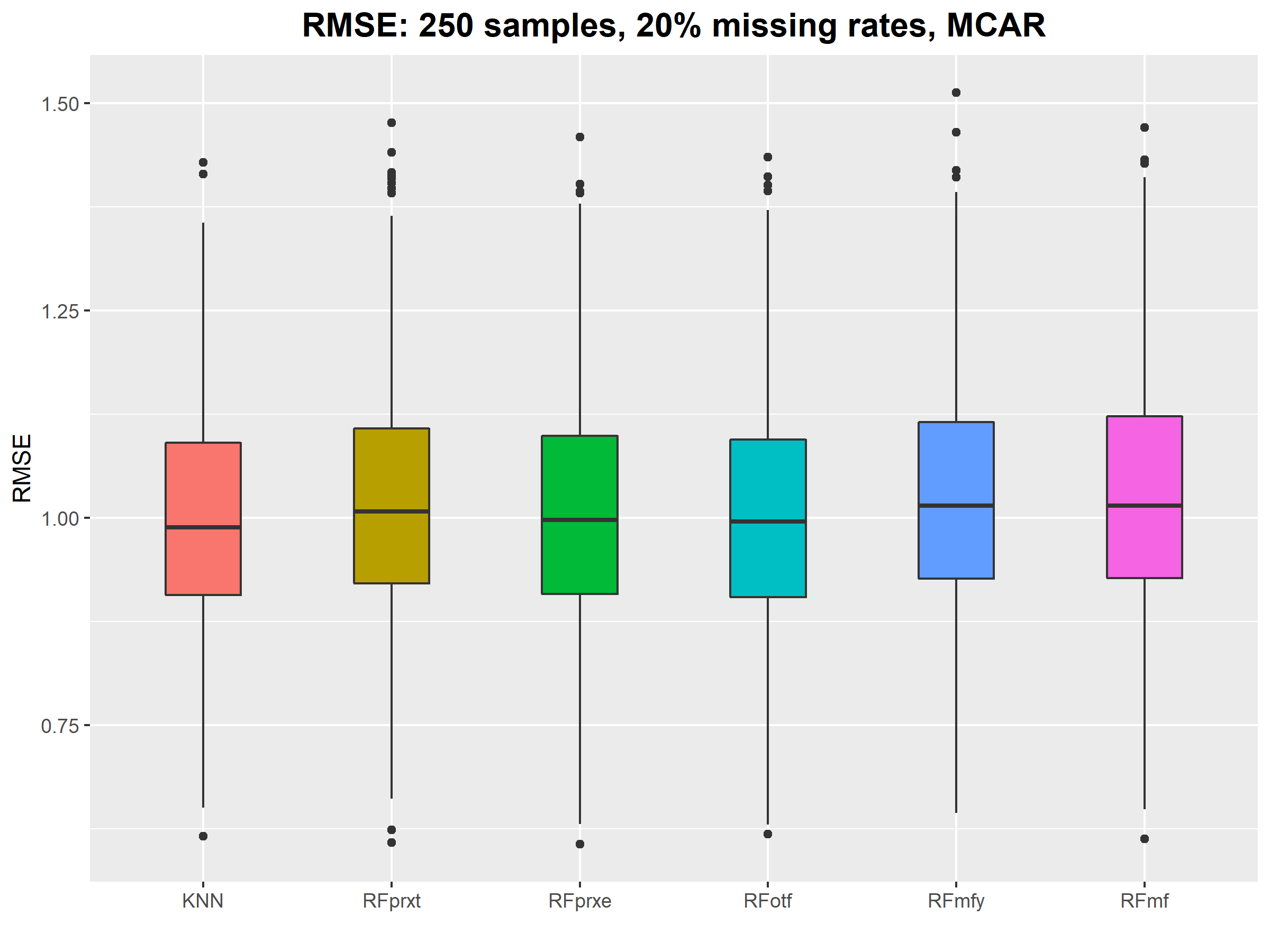

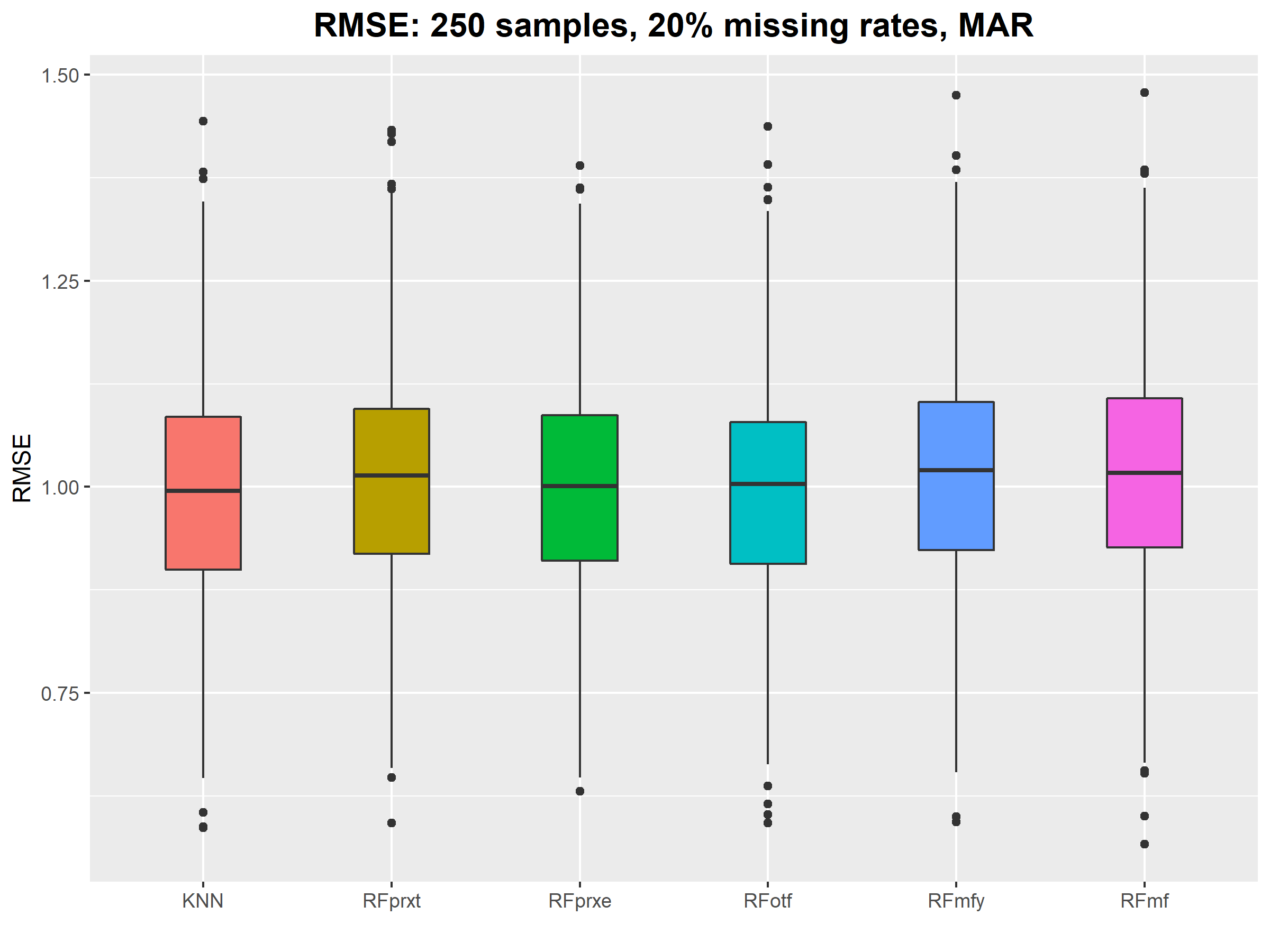

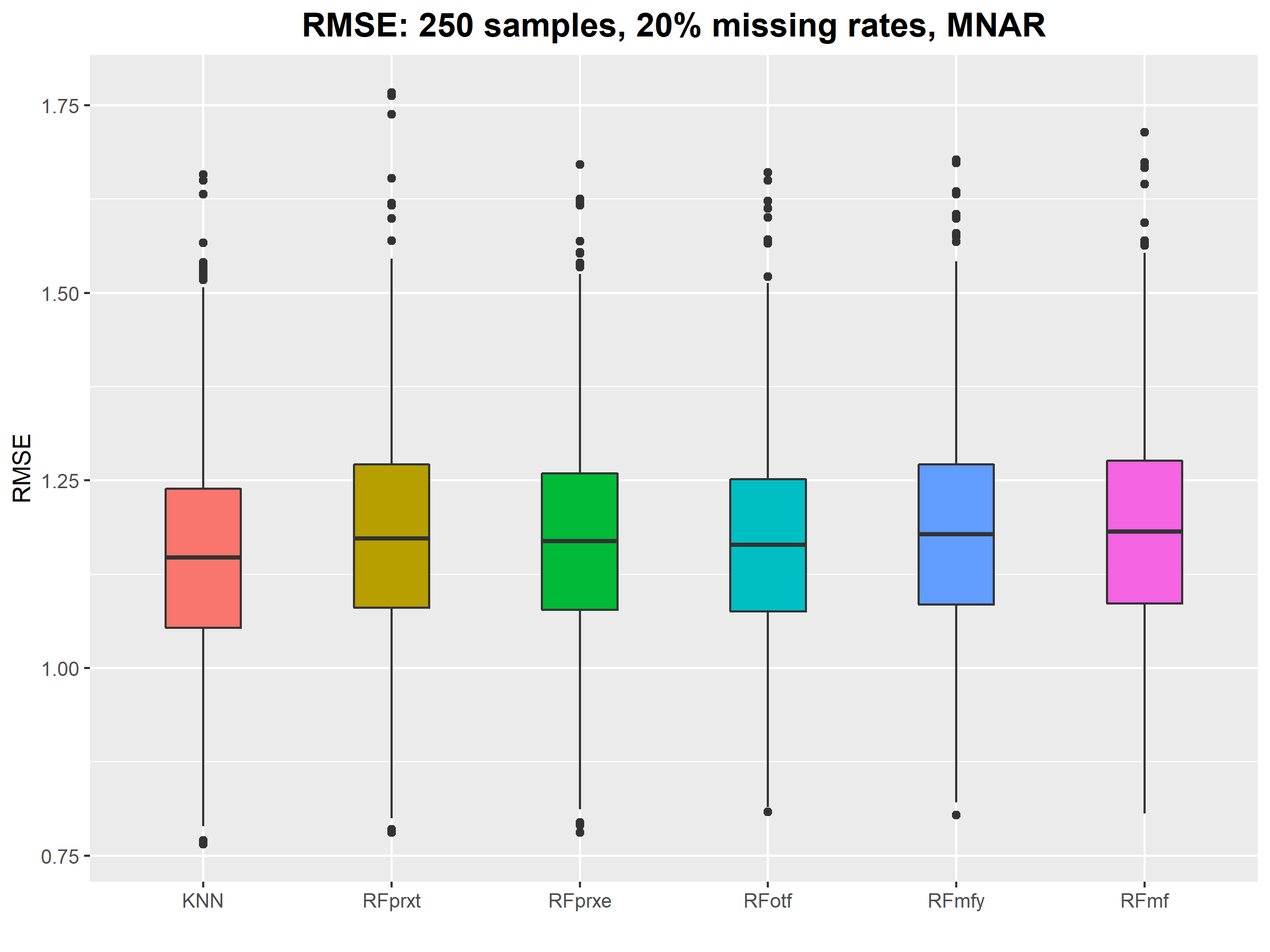

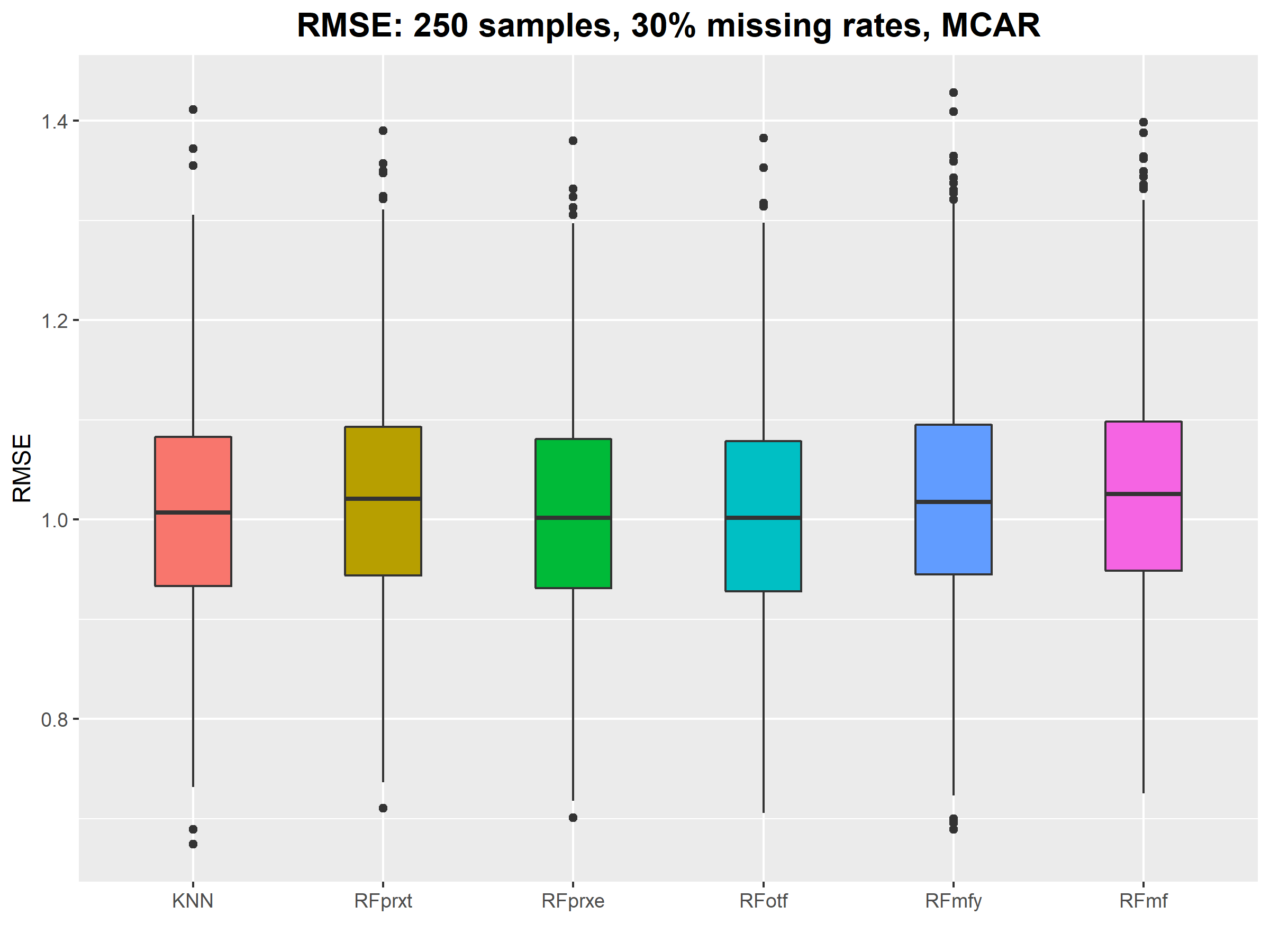

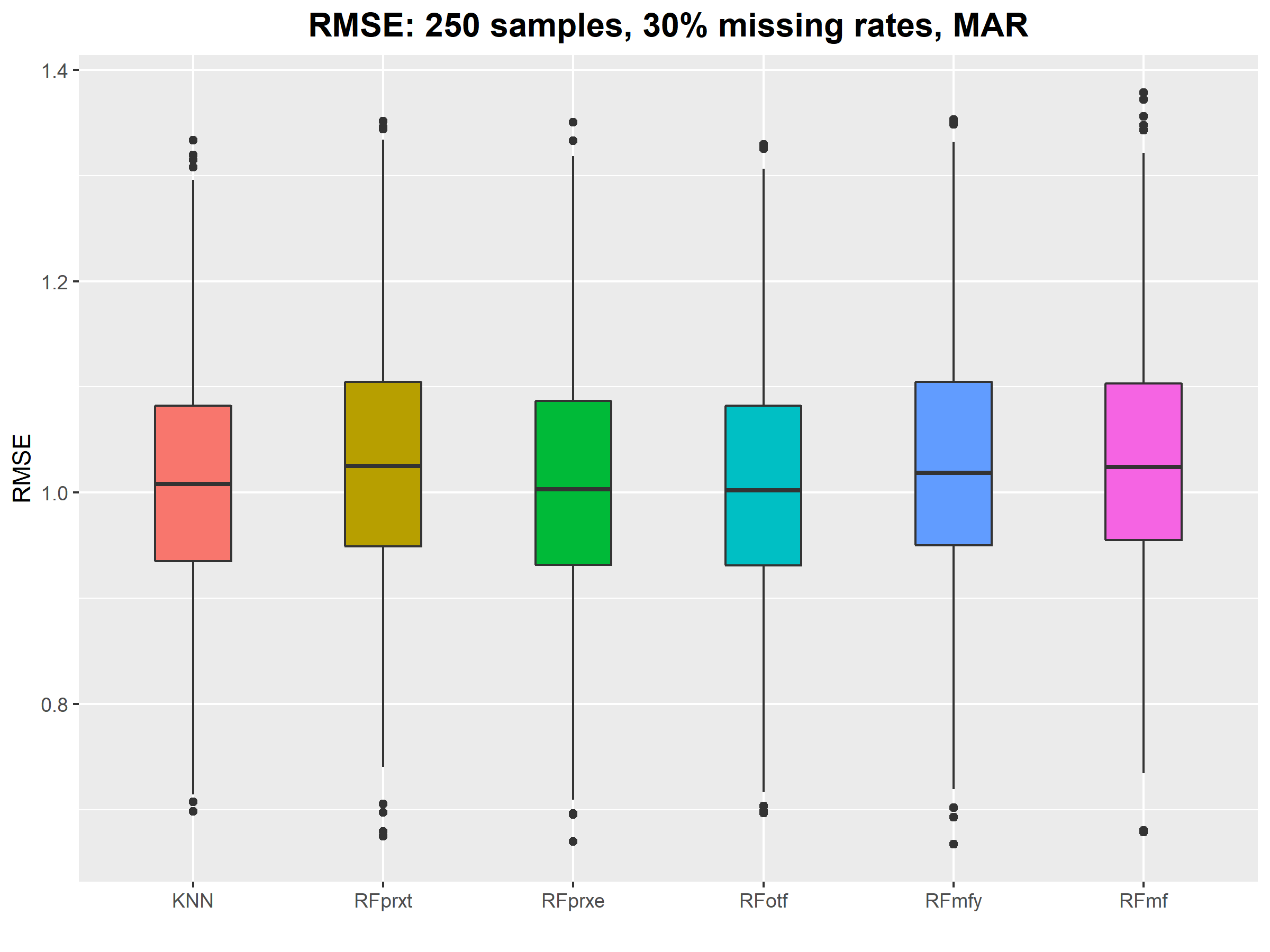

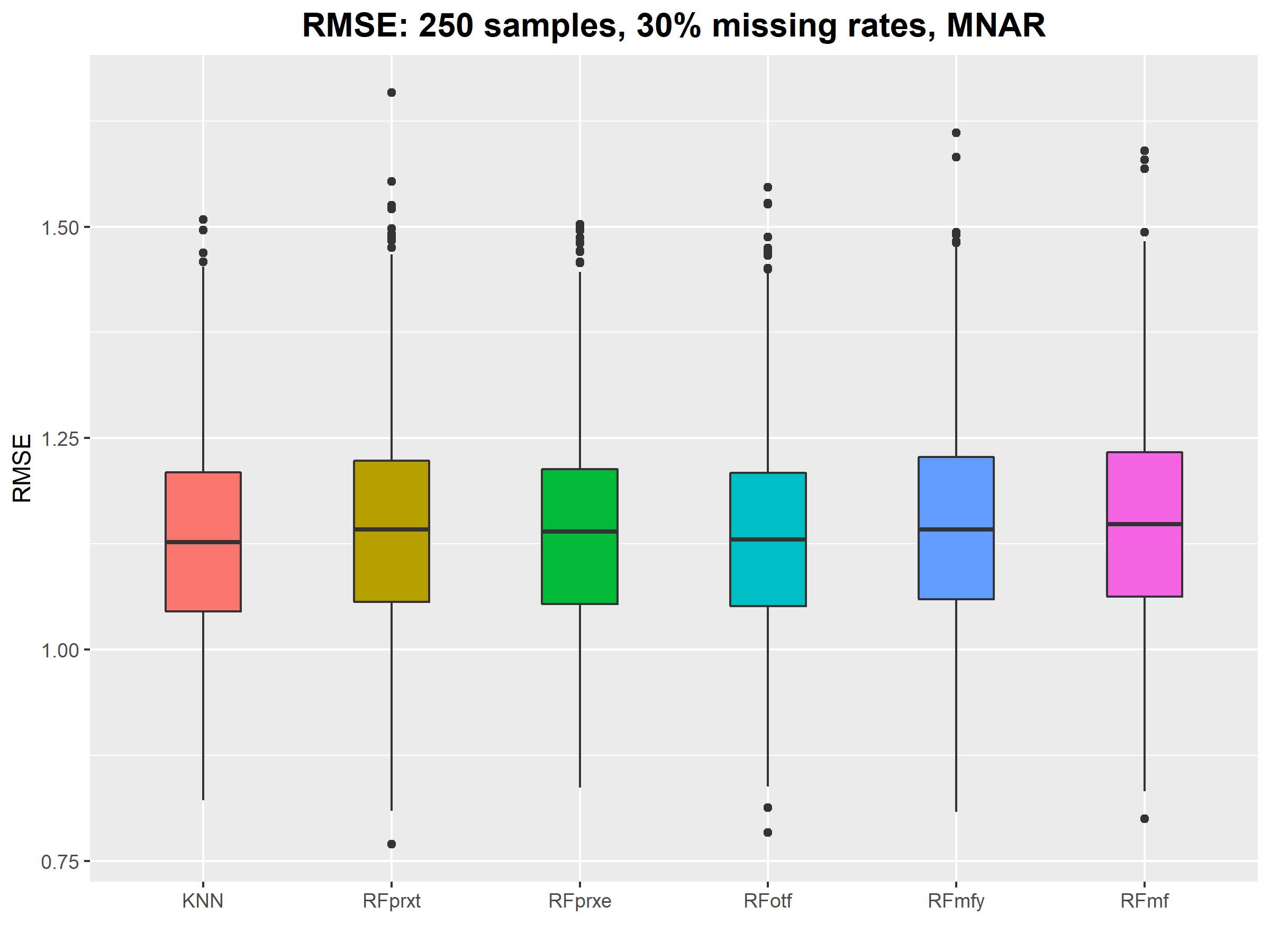


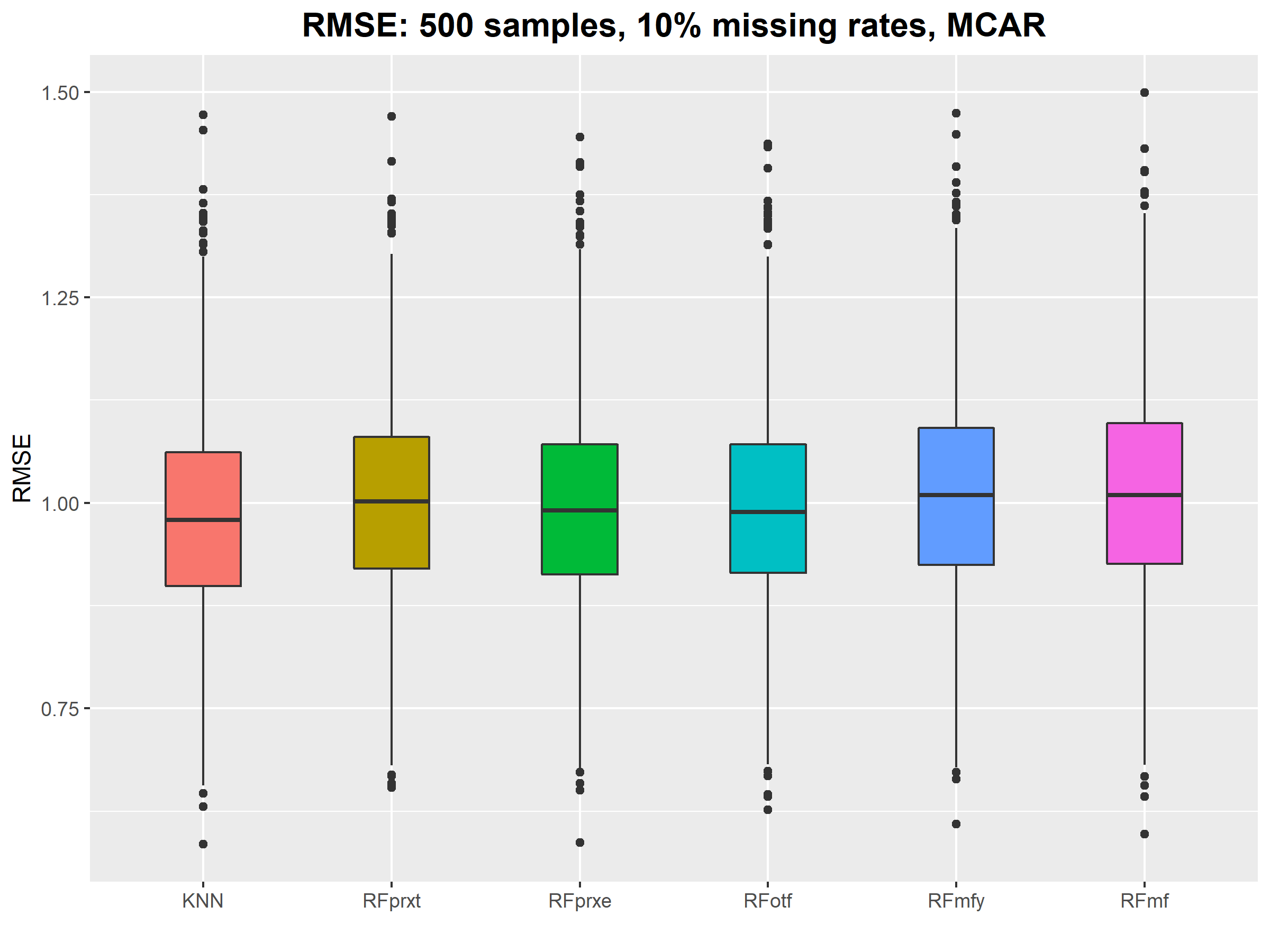

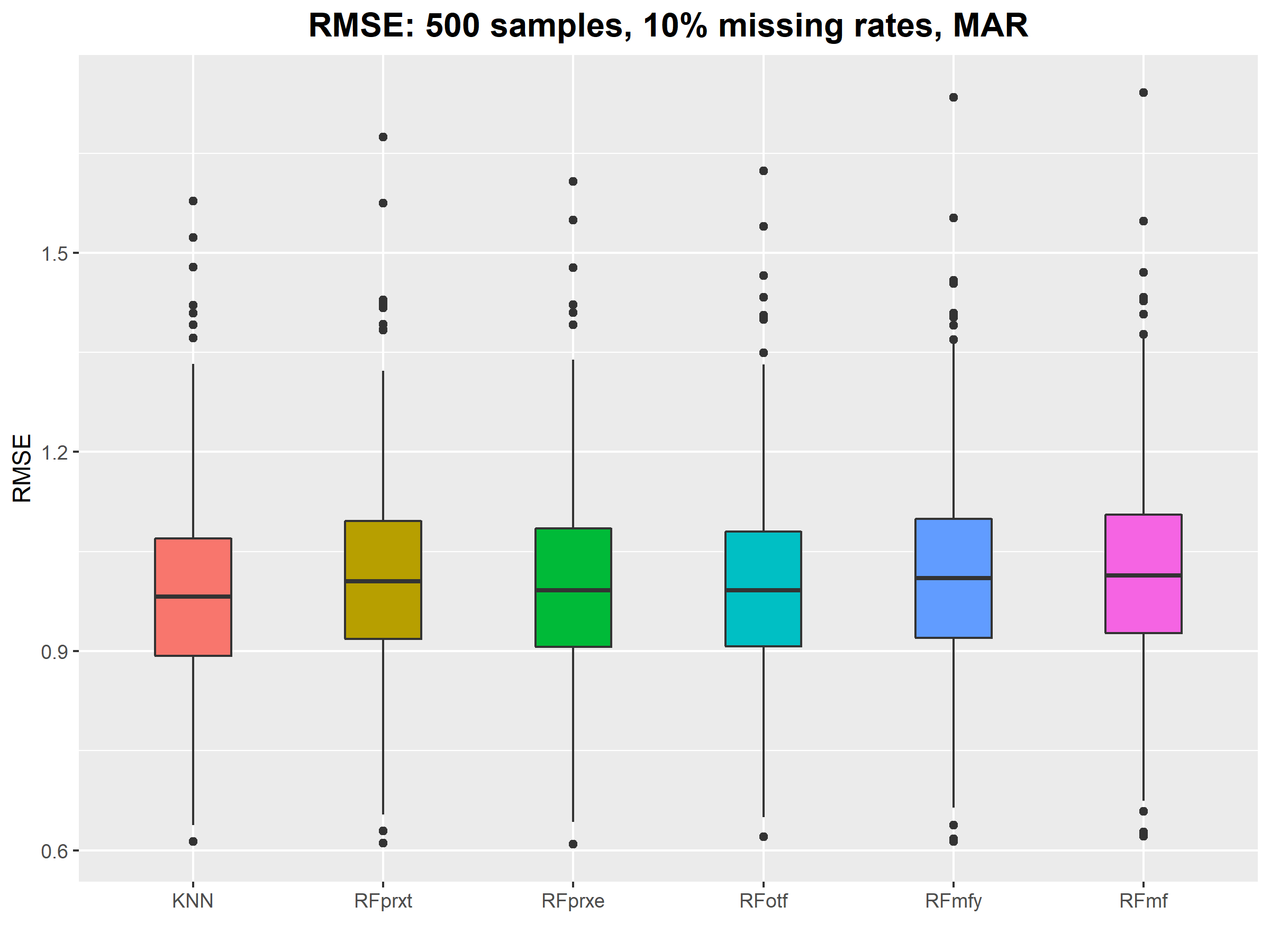

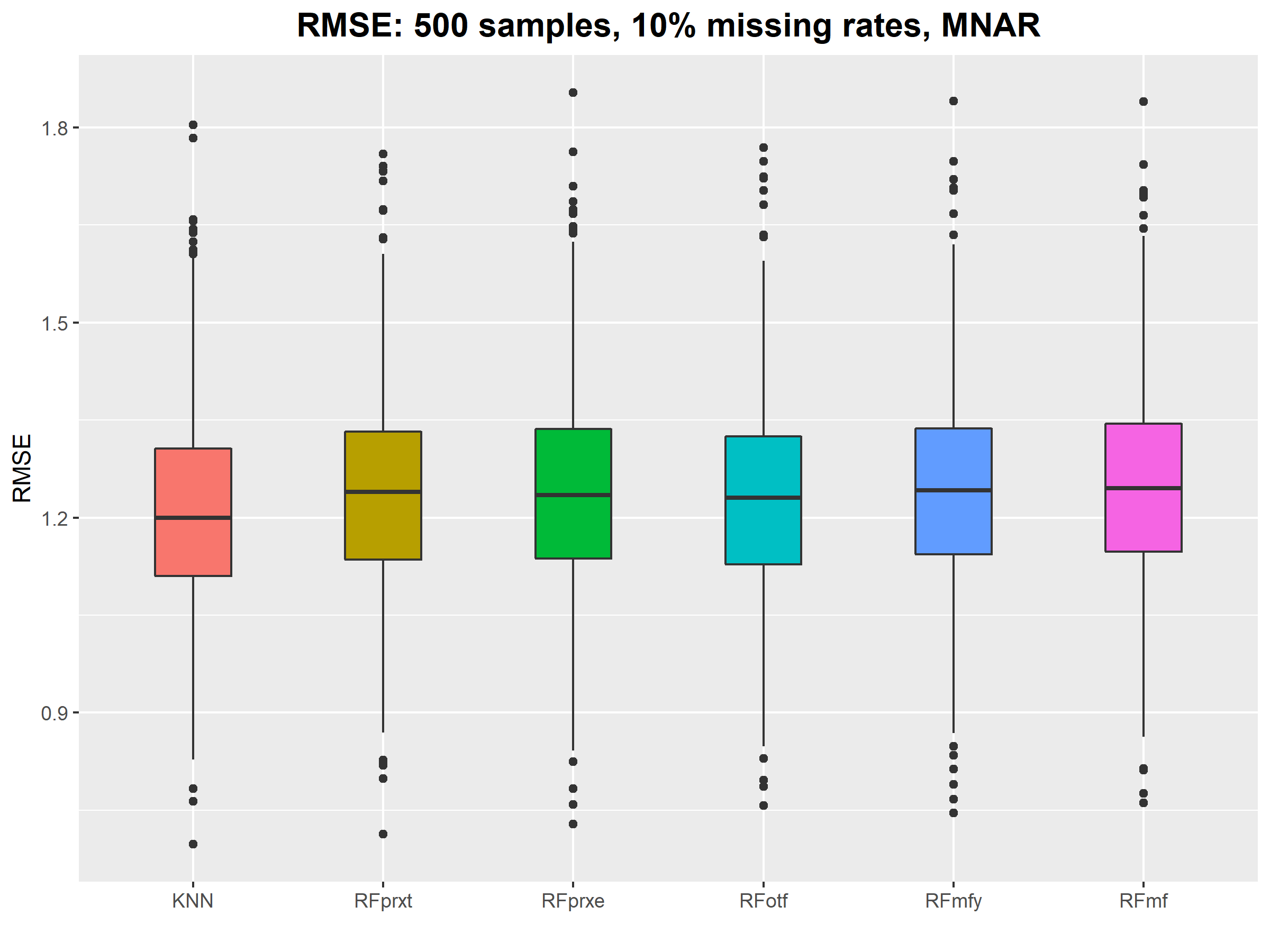

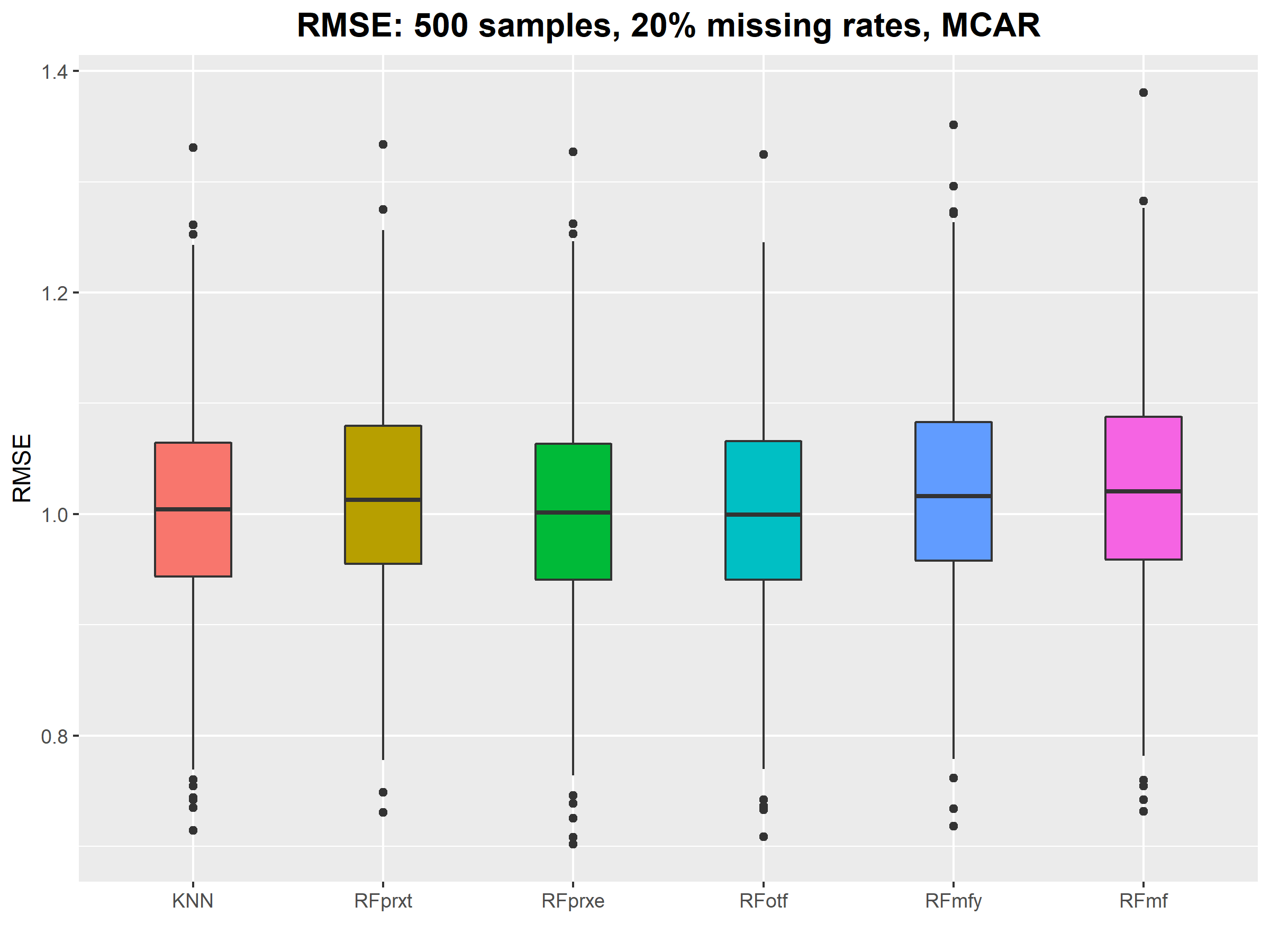

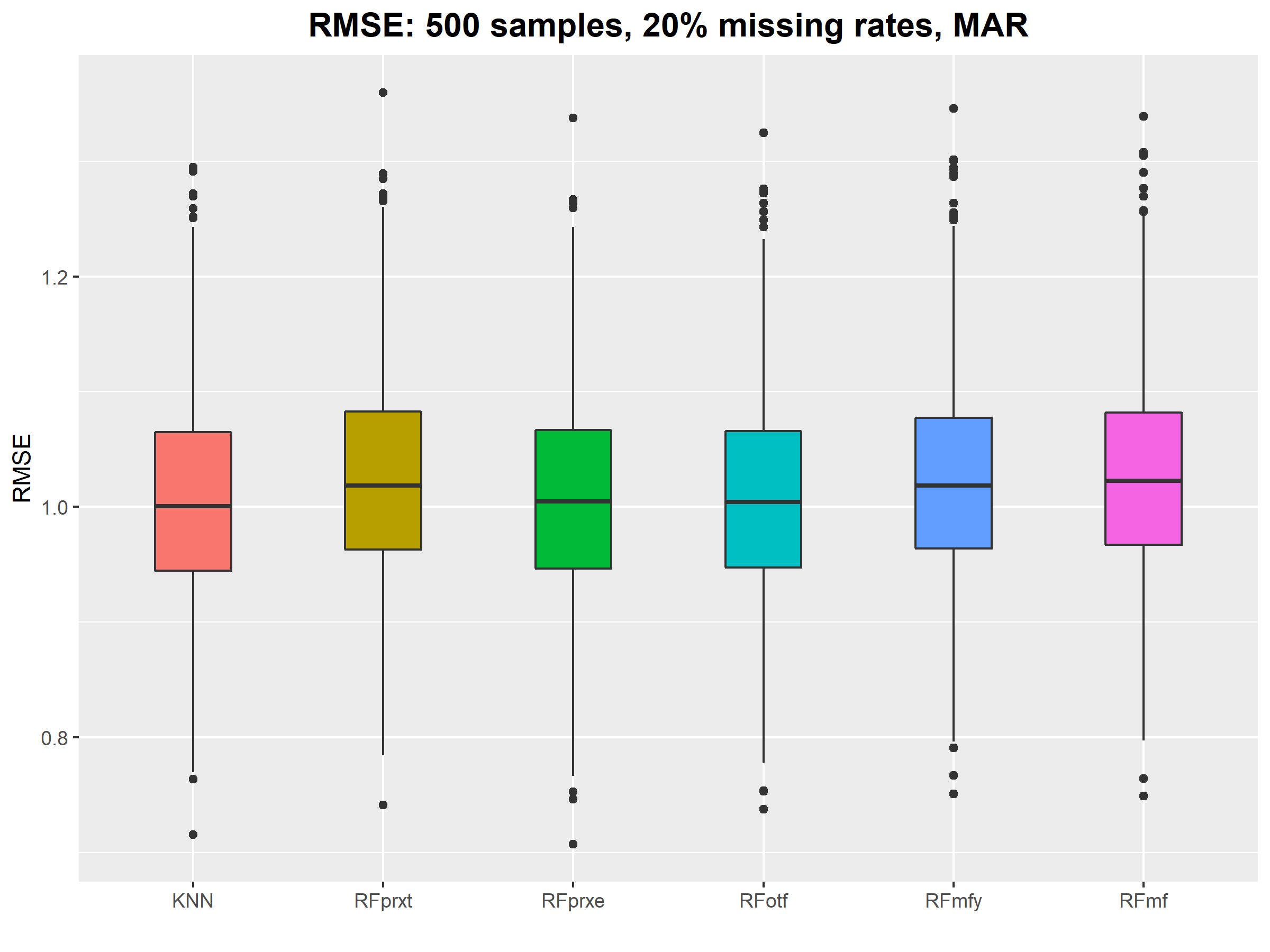

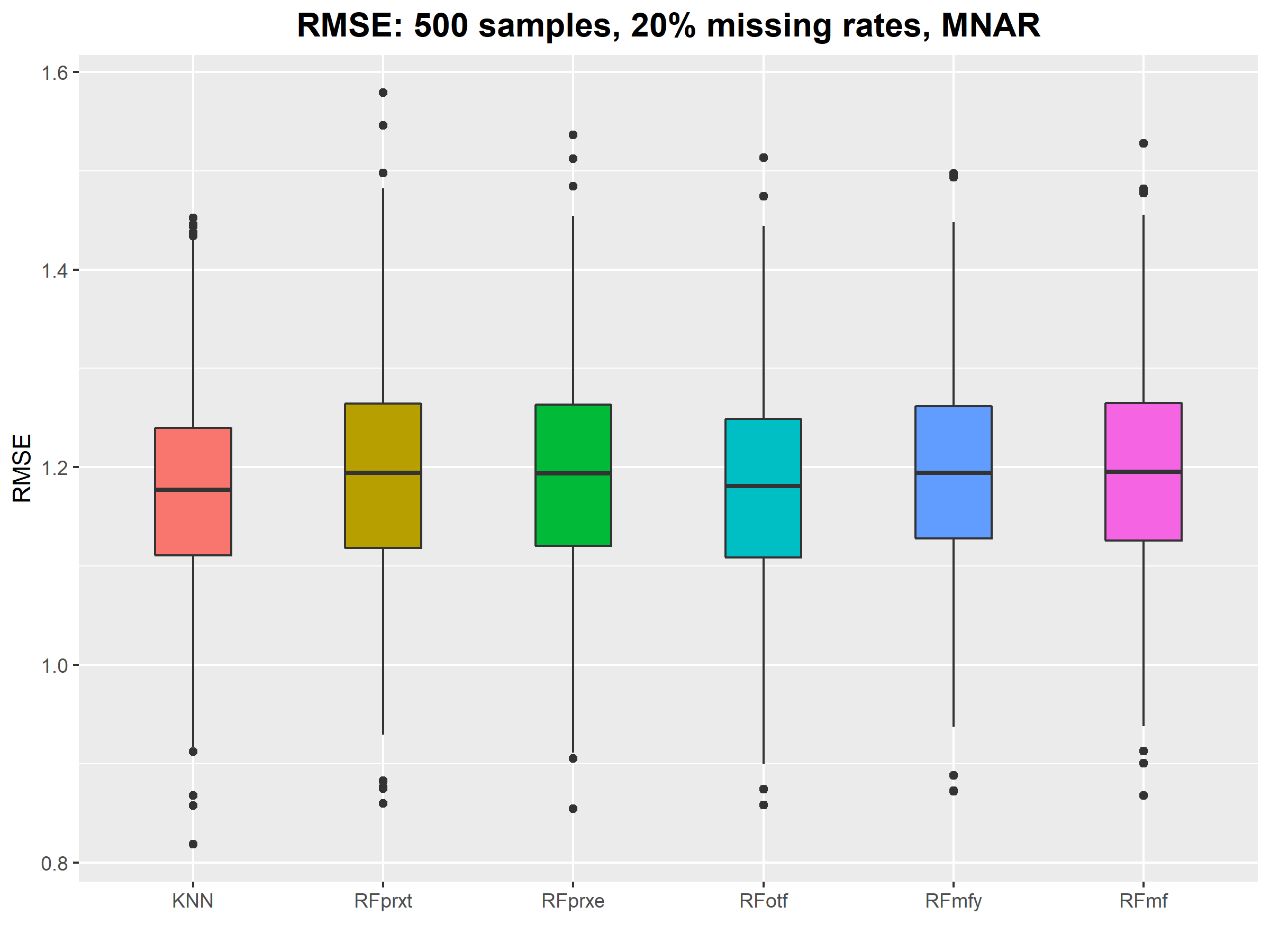

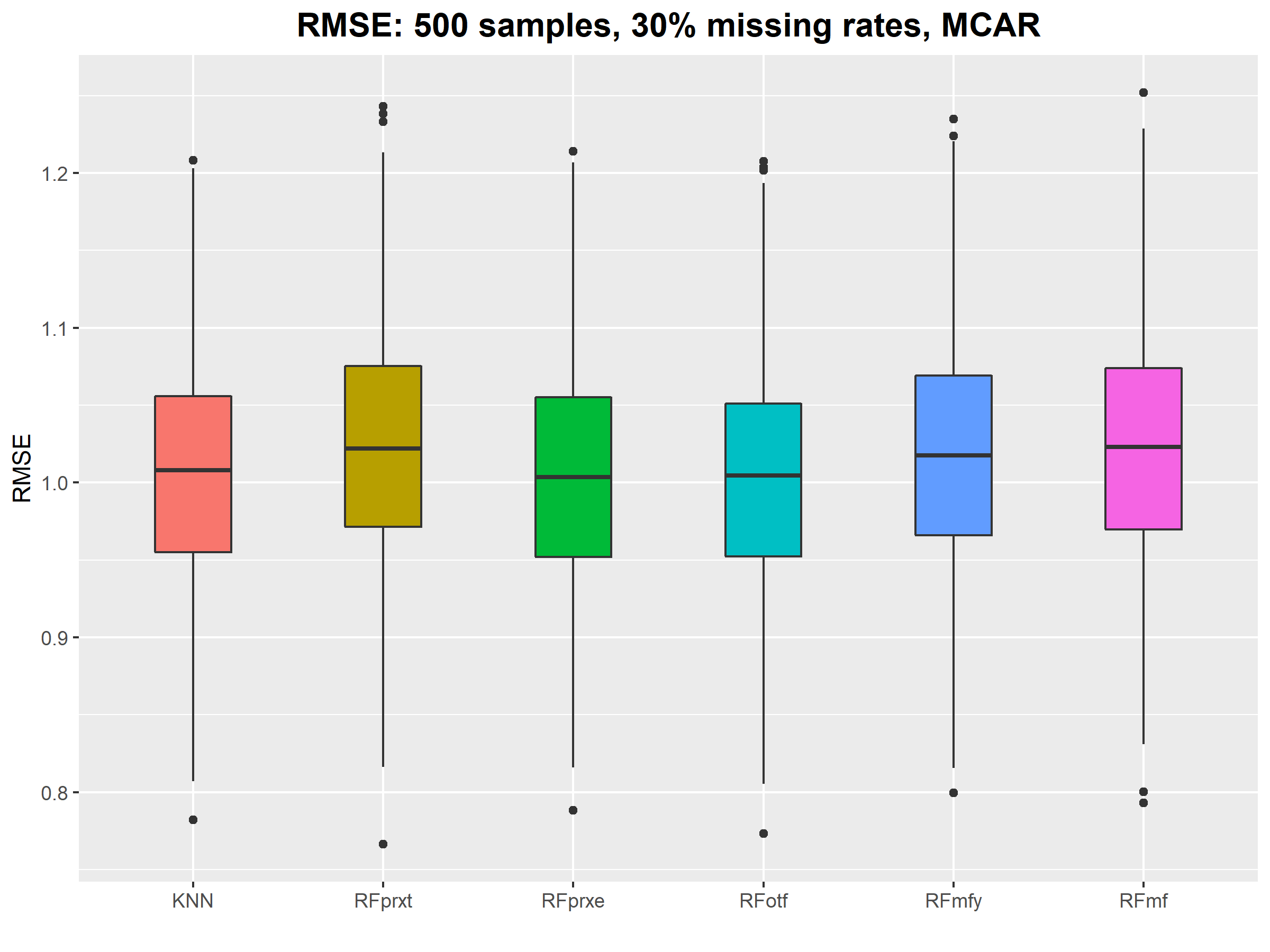

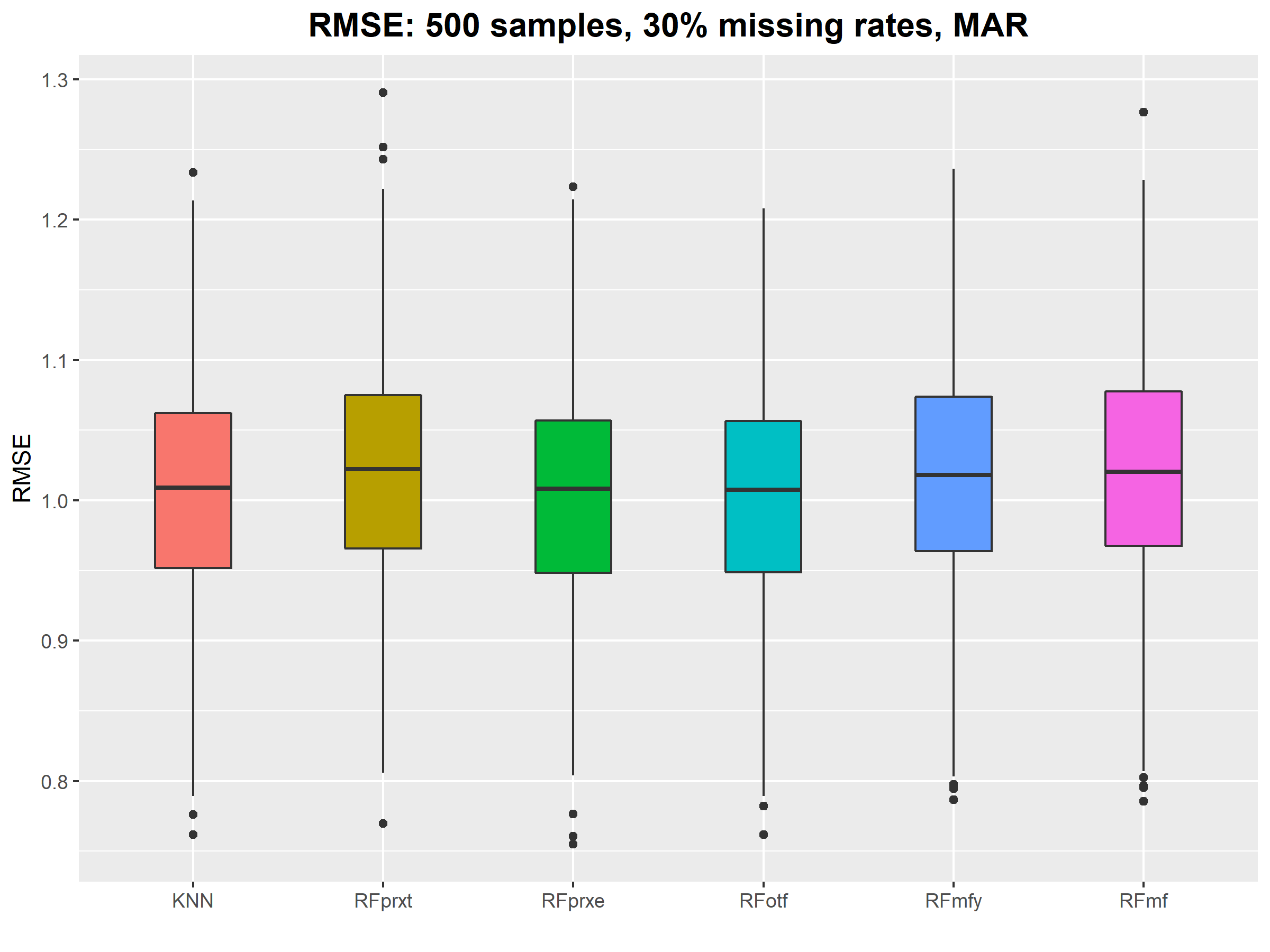

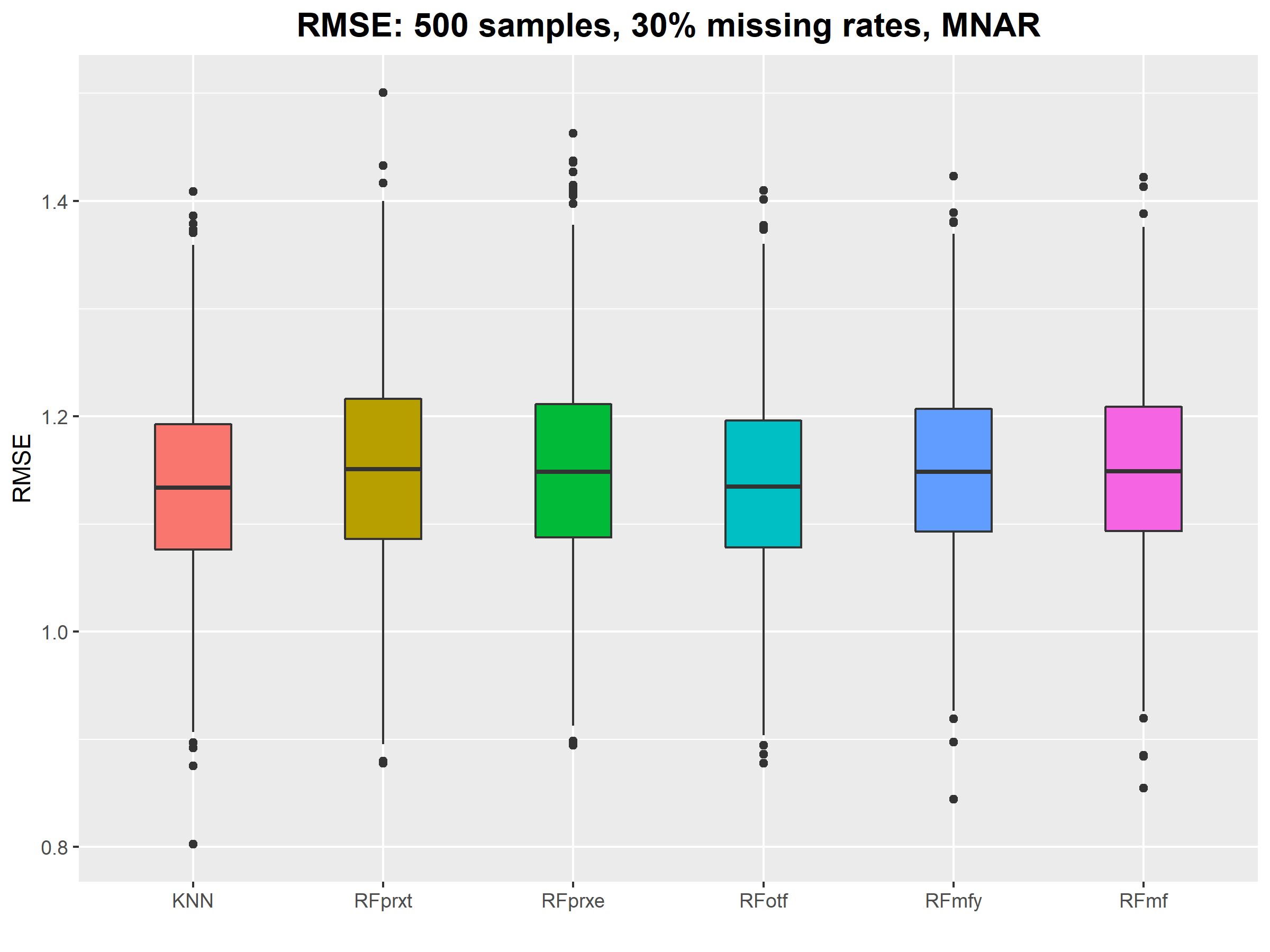


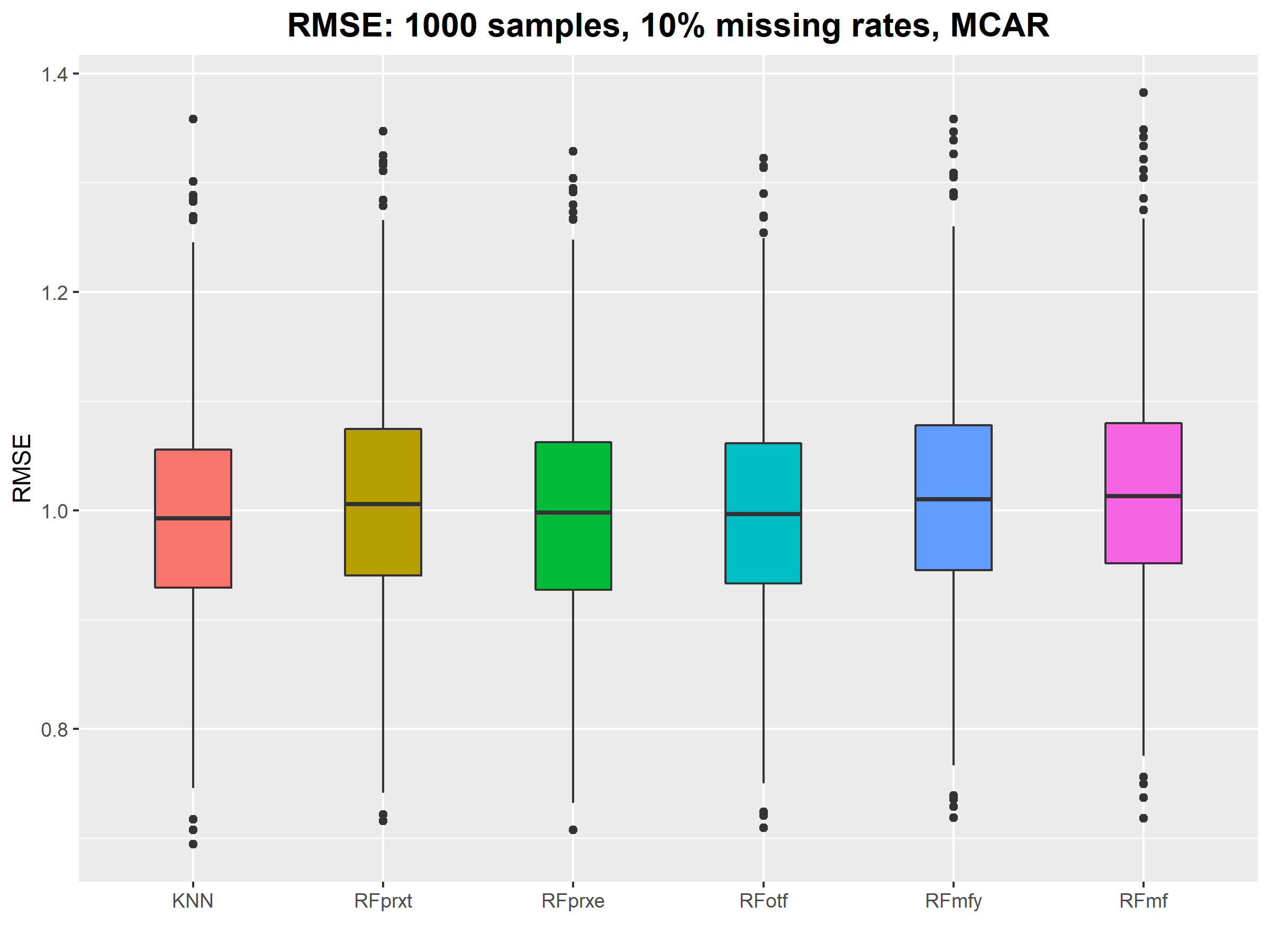

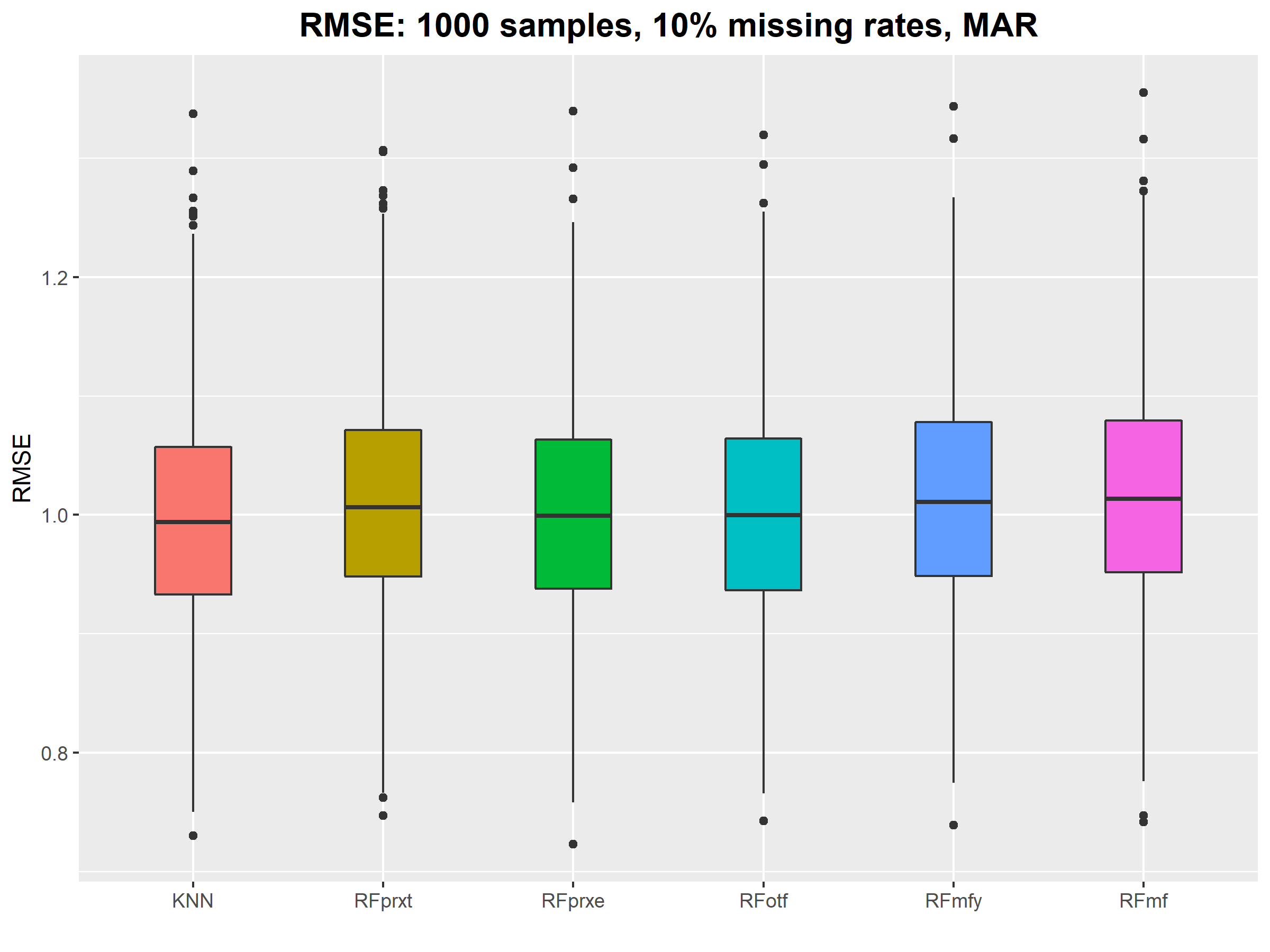

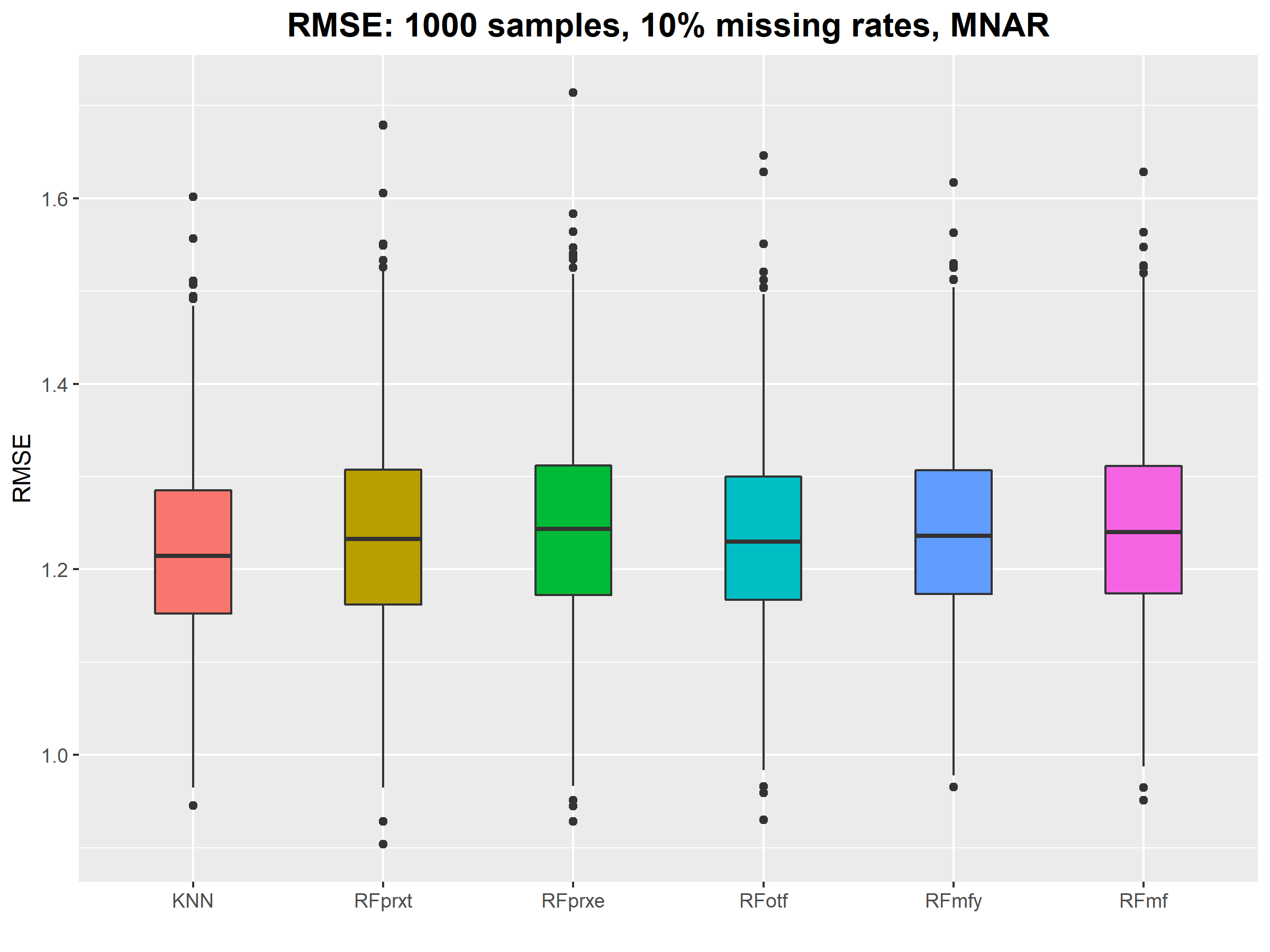

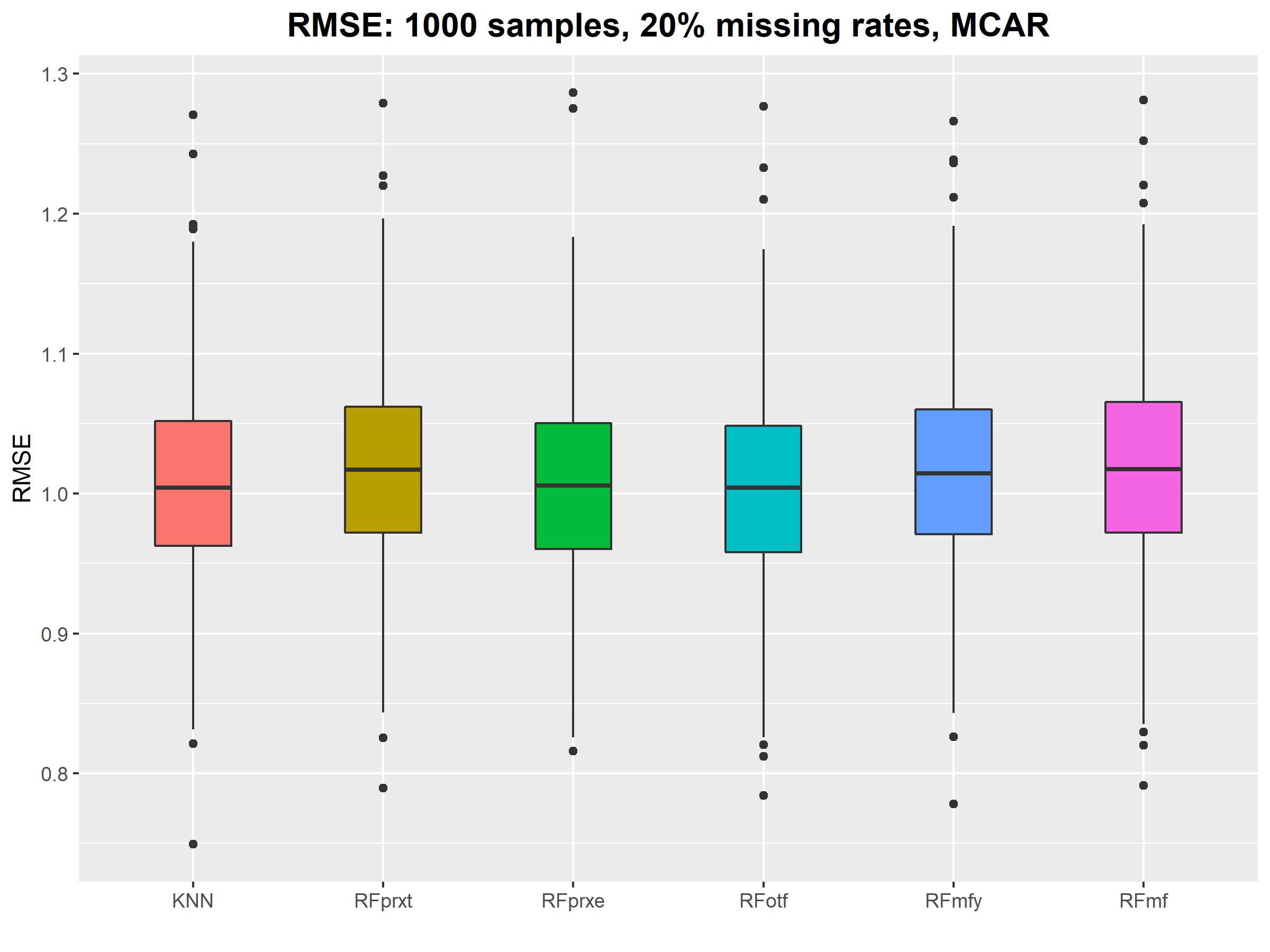

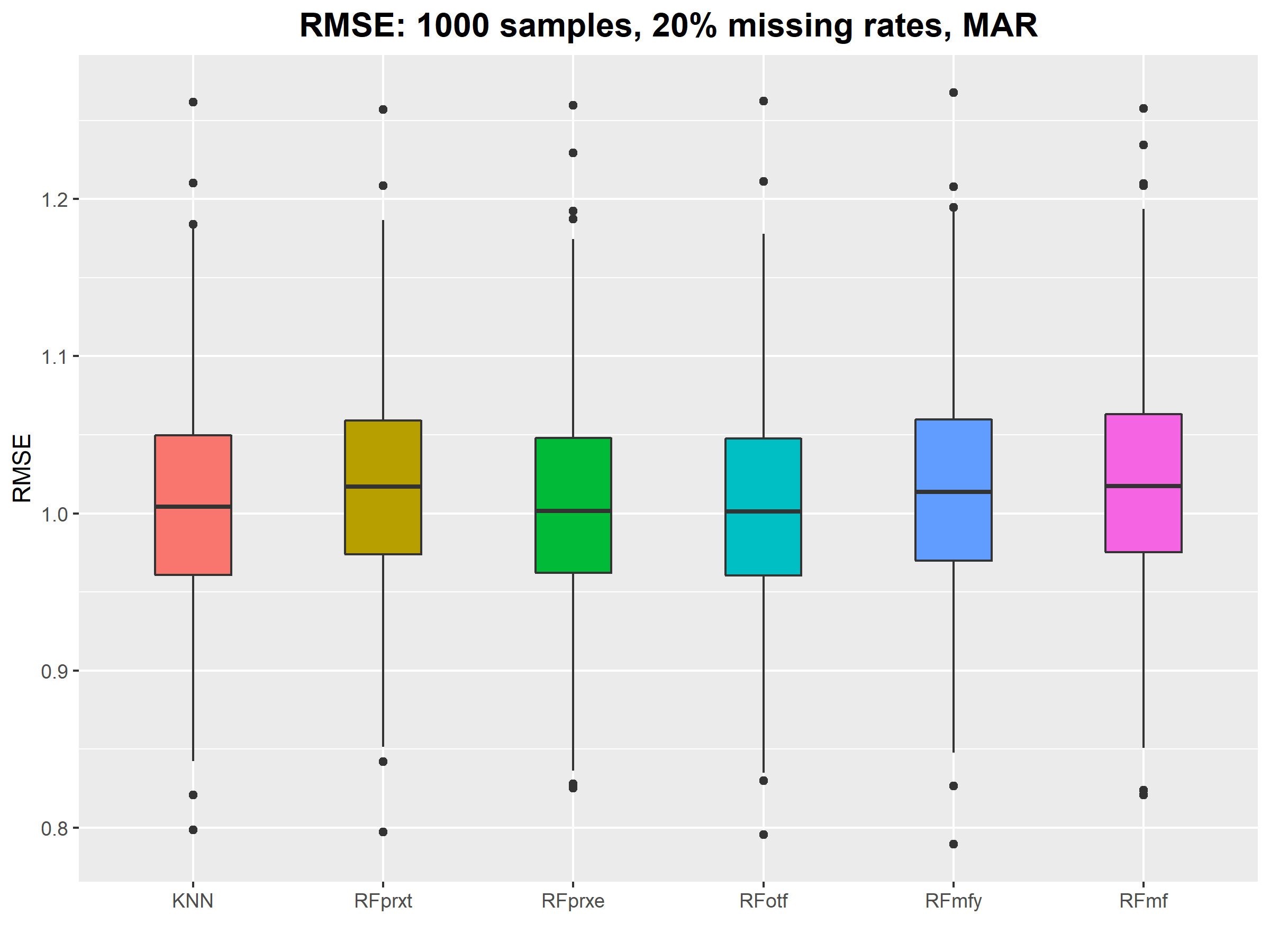

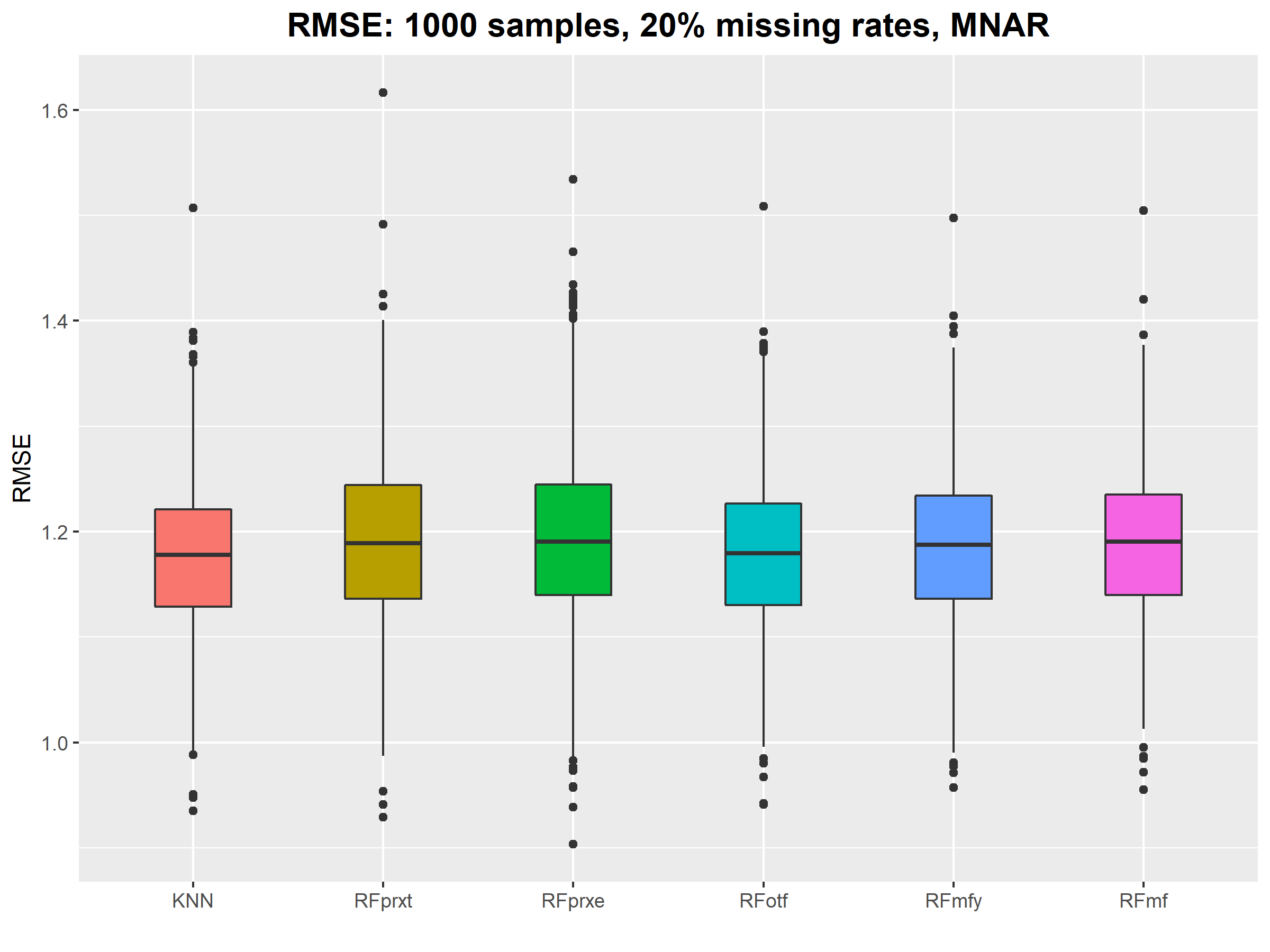

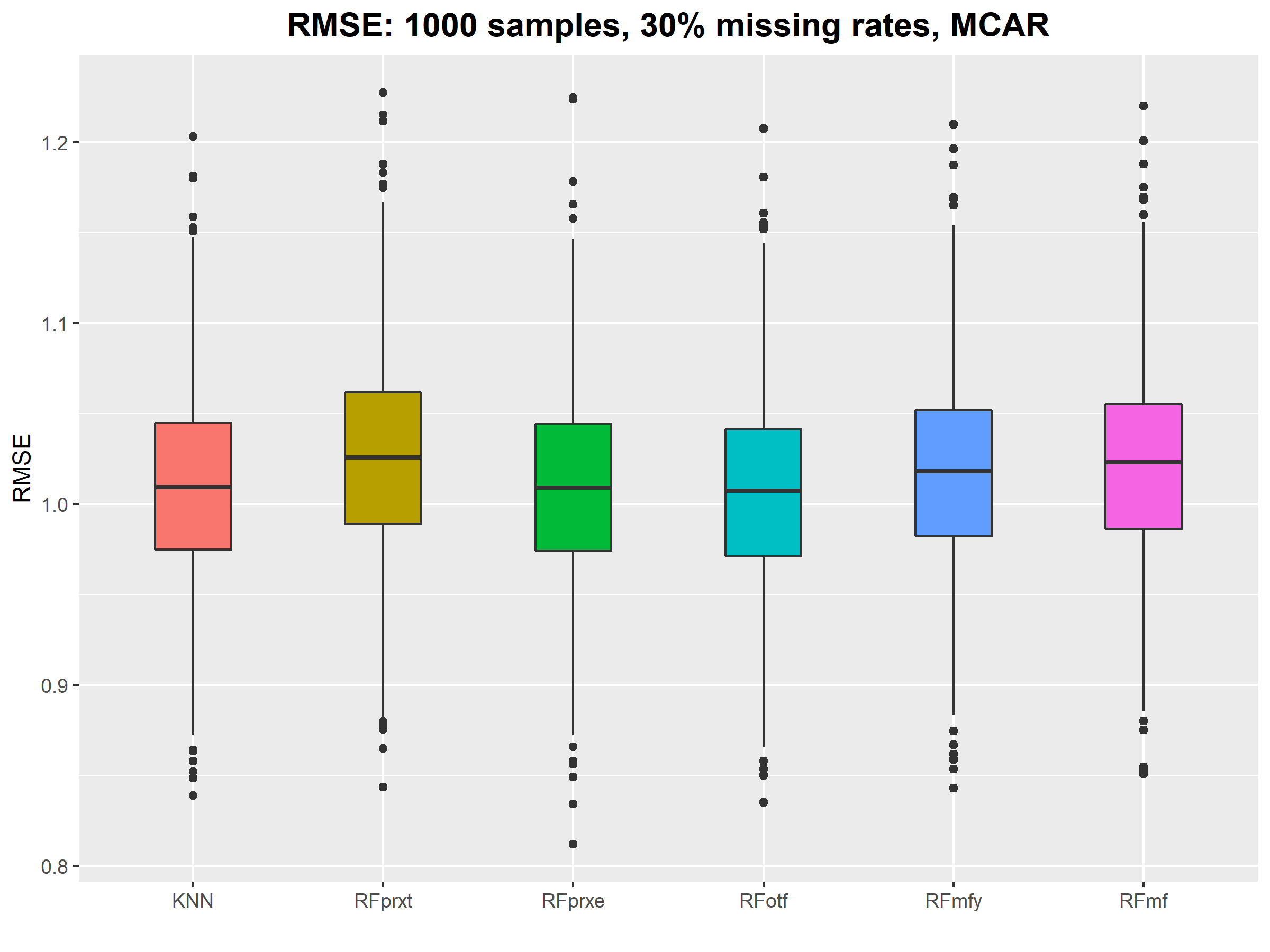

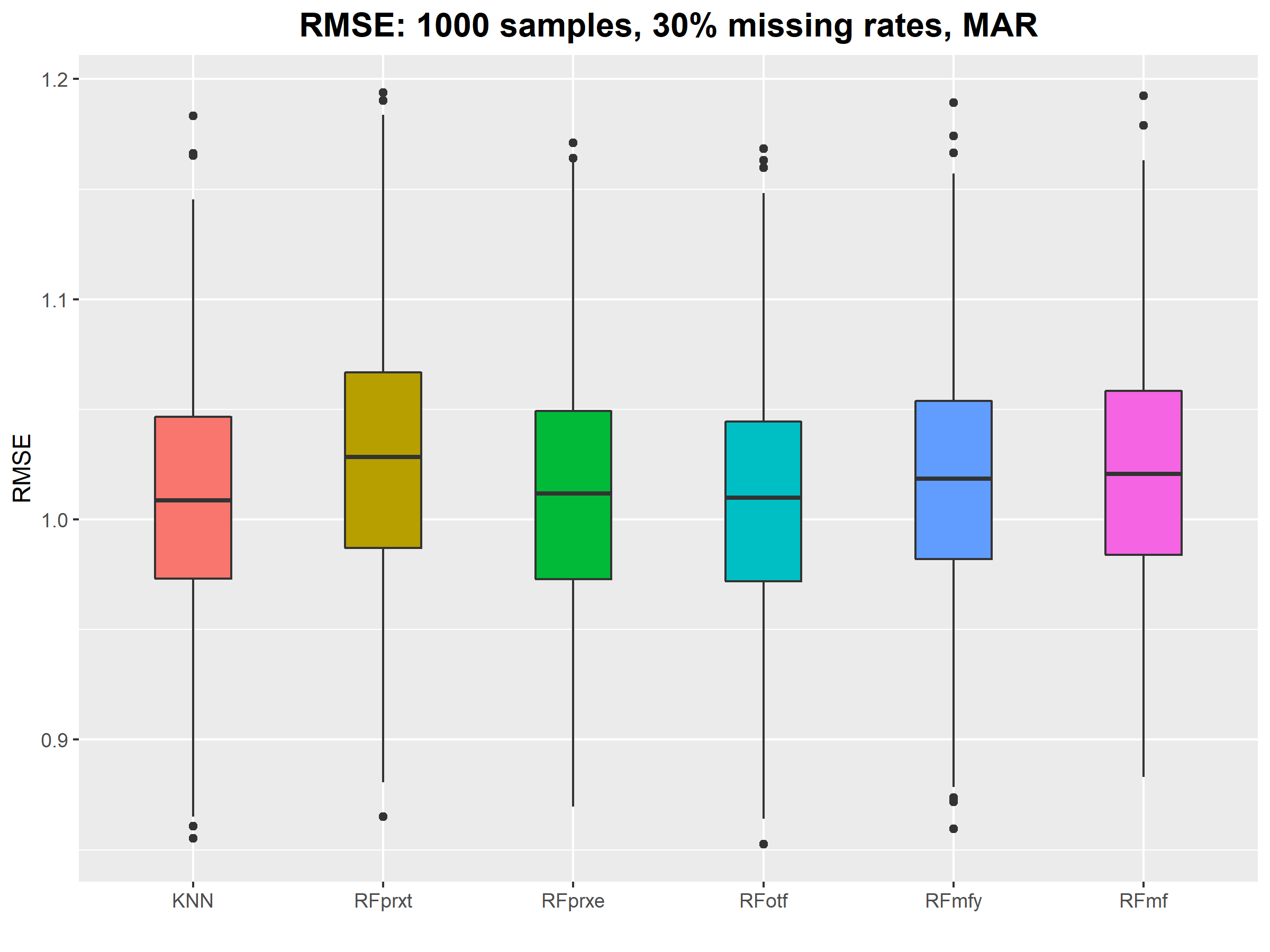

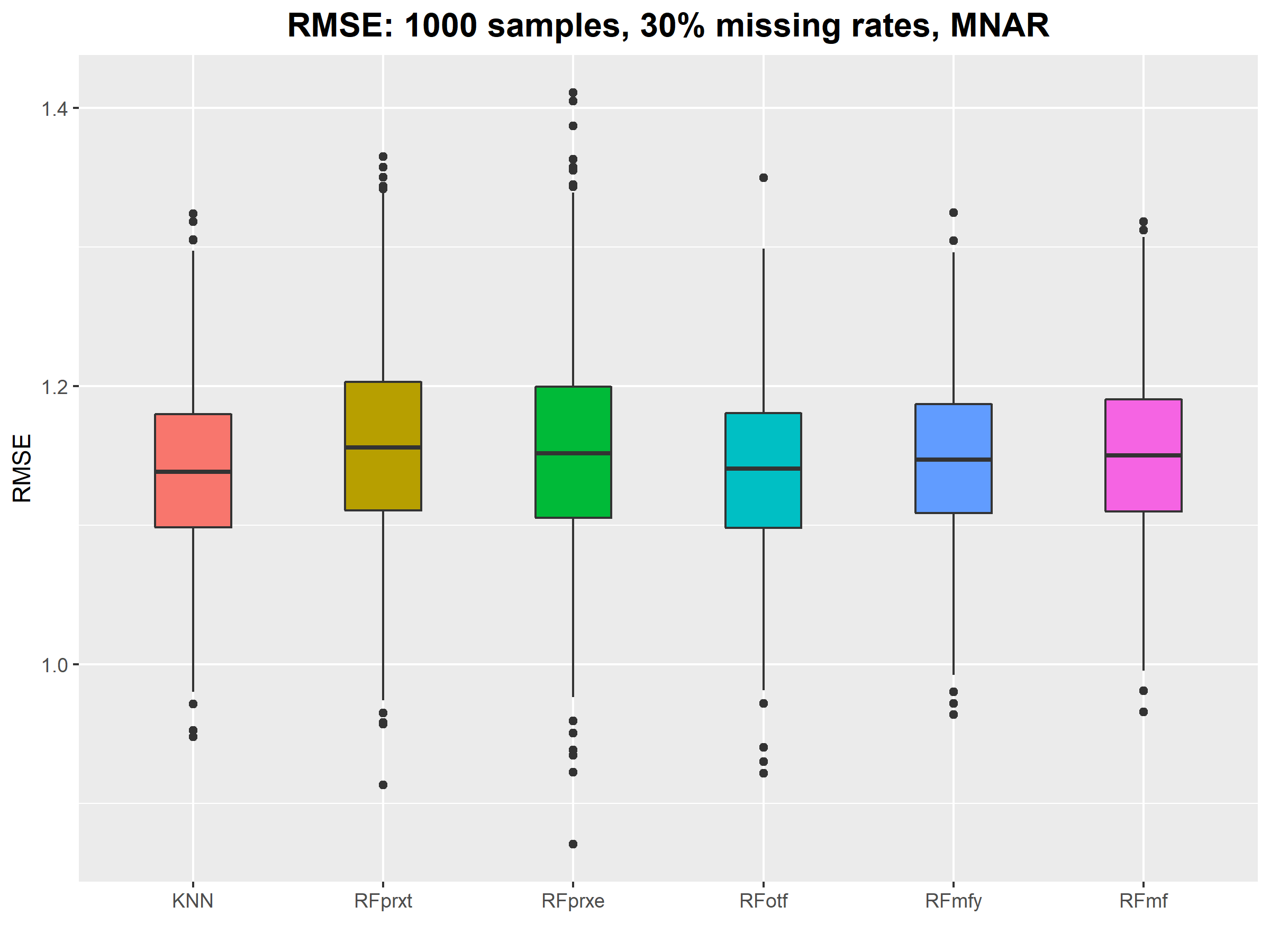


Table S1: Detailed results of the PFC

| n_sample | missing_rate | amp | method | pfc |
| --- | --- | --- | --- | --- |
| 100 | 0.1 | MCAR | KNN | 0.36468302 |
| 100 | 0.1 | MCAR | RFprxt | 0.43841847 |
| 100 | 0.1 | MCAR | RFprxe | 0.47805835 |
| 100 | 0.1 | MCAR | RFotf | 0.30615842 |
| 100 | 0.1 | MCAR | RFmfy | 0.33860324 |
| 100 | 0.1 | MCAR | RFmf | 0.34521592 |
| 100 | 0.1 | MAR | KNN | 0.37804527 |
| 100 | 0.1 | MAR | RFprxt | 0.44589749 |
| 100 | 0.1 | MAR | RFprxe | 0.47837893 |
| 100 | 0.1 | MAR | RFotf | 0.3147041 |
| 100 | 0.1 | MAR | RFmfy | 0.34041915 |
| 100 | 0.1 | MAR | RFmf | 0.35055224 |
| 100 | 0.1 | MNAR | KNN | 0.63615815 |
| 100 | 0.1 | MNAR | RFprxt | 0.52747884 |
| 100 | 0.1 | MNAR | RFprxe | 0.49830112 |
| 100 | 0.1 | MNAR | RFotf | 0.7024686 |
| 100 | 0.1 | MNAR | RFmfy | 0.67188728 |
| 100 | 0.1 | MNAR | RFmf | 0.66078123 |
| 100 | 0.2 | MCAR | KNN | 0.36814763 |
| 100 | 0.2 | MCAR | RFprxt | 0.44298666 |
| 100 | 0.2 | MCAR | RFprxe | 0.48908403 |
| 100 | 0.2 | MCAR | RFotf | 0.30713588 |
| 100 | 0.2 | MCAR | RFmfy | 0.34207528 |
| 100 | 0.2 | MCAR | RFmf | 0.34557008 |
| 100 | 0.2 | MAR | KNN | 0.38538187 |
| 100 | 0.2 | MAR | RFprxt | 0.44880555 |
| 100 | 0.2 | MAR | RFprxe | 0.49846121 |
| 100 | 0.2 | MAR | RFotf | 0.31835649 |
| 100 | 0.2 | MAR | RFmfy | 0.35026805 |
| 100 | 0.2 | MAR | RFmf | 0.35500821 |
| 100 | 0.2 | MNAR | KNN | 0.61170234 |
| 100 | 0.2 | MNAR | RFprxt | 0.51000474 |
| 100 | 0.2 | MNAR | RFprxe | 0.48621261 |
| 100 | 0.2 | MNAR | RFotf | 0.64703097 |
| 100 | 0.2 | MNAR | RFmfy | 0.63111025 |
| 100 | 0.2 | MNAR | RFmf | 0.63044938 |
| 100 | 0.3 | MCAR | KNN | 0.3645221 |
| 100 | 0.3 | MCAR | RFprxt | 0.45887949 |
| 100 | 0.3 | MCAR | RFprxe | 0.48928124 |
| 100 | 0.3 | MCAR | RFotf | 0.30491435 |
| 100 | 0.3 | MCAR | RFmfy | 0.33997283 |
| 100 | 0.3 | MCAR | RFmf | 0.34470376 |
| 100 | 0.3 | MAR | KNN | 0.36573318 |
| 100 | 0.3 | MAR | RFprxt | 0.4518071 |
| 100 | 0.3 | MAR | RFprxe | 0.48850743 |
| 100 | 0.3 | MAR | RFotf | 0.3058132 |
| 100 | 0.3 | MAR | RFmfy | 0.33712053 |
| 100 | 0.3 | MAR | RFmf | 0.34406864 |
| 100 | 0.3 | MNAR | KNN | 0.57081175 |
| 100 | 0.3 | MNAR | RFprxt | 0.50868515 |
| 100 | 0.3 | MNAR | RFprxe | 0.49980566 |
| 100 | 0.3 | MNAR | RFotf | 0.59051775 |
| 100 | 0.3 | MNAR | RFmfy | 0.58105625 |
| 100 | 0.3 | MNAR | RFmf | 0.5787665 |
| 250 | 0.1 | MCAR | KNN | 0.35017755 |
| 250 | 0.1 | MCAR | RFprxt | 0.42671109 |
| 250 | 0.1 | MCAR | RFprxe | 0.4816913 |
| 250 | 0.1 | MCAR | RFotf | 0.30604801 |
| 250 | 0.1 | MCAR | RFmfy | 0.32666903 |
| 250 | 0.1 | MCAR | RFmf | 0.33041494 |
| 250 | 0.1 | MAR | KNN | 0.36171523 |
| 250 | 0.1 | MAR | RFprxt | 0.43088937 |
| 250 | 0.1 | MAR | RFprxe | 0.48282124 |
| 250 | 0.1 | MAR | RFotf | 0.30686877 |
| 250 | 0.1 | MAR | RFmfy | 0.32876449 |
| 250 | 0.1 | MAR | RFmf | 0.33242207 |
| 250 | 0.1 | MNAR | KNN | 0.66102863 |
| 250 | 0.1 | MNAR | RFprxt | 0.55862877 |
| 250 | 0.1 | MNAR | RFprxe | 0.49868687 |
| 250 | 0.1 | MNAR | RFotf | 0.71214789 |
| 250 | 0.1 | MNAR | RFmfy | 0.6913236 |
| 250 | 0.1 | MNAR | RFmf | 0.68469728 |
| 250 | 0.2 | MCAR | KNN | 0.34344186 |
| 250 | 0.2 | MCAR | RFprxt | 0.43158144 |
| 250 | 0.2 | MCAR | RFprxe | 0.48270245 |
| 250 | 0.2 | MCAR | RFotf | 0.30218527 |
| 250 | 0.2 | MCAR | RFmfy | 0.32502655 |
| 250 | 0.2 | MCAR | RFmf | 0.32923179 |
| 250 | 0.2 | MAR | KNN | 0.35637449 |
| 250 | 0.2 | MAR | RFprxt | 0.43951309 |
| 250 | 0.2 | MAR | RFprxe | 0.48934182 |
| 250 | 0.2 | MAR | RFotf | 0.3078793 |
| 250 | 0.2 | MAR | RFmfy | 0.33087226 |
| 250 | 0.2 | MAR | RFmf | 0.33782148 |
| 250 | 0.2 | MNAR | KNN | 0.62862097 |
| 250 | 0.2 | MNAR | RFprxt | 0.53571354 |
| 250 | 0.2 | MNAR | RFprxe | 0.49986128 |
| 250 | 0.2 | MNAR | RFotf | 0.65226143 |
| 250 | 0.2 | MNAR | RFmfy | 0.64194965 |
| 250 | 0.2 | MNAR | RFmf | 0.63786894 |
| 250 | 0.3 | MCAR | KNN | 0.3468841 |
| 250 | 0.3 | MCAR | RFprxt | 0.43535979 |
| 250 | 0.3 | MCAR | RFprxe | 0.47793639 |
| 250 | 0.3 | MCAR | RFotf | 0.31071359 |
| 250 | 0.3 | MCAR | RFmfy | 0.33537357 |
| 250 | 0.3 | MCAR | RFmf | 0.34055575 |
| 250 | 0.3 | MAR | KNN | 0.36144333 |
| 250 | 0.3 | MAR | RFprxt | 0.44458168 |
| 250 | 0.3 | MAR | RFprxe | 0.49013721 |
| 250 | 0.3 | MAR | RFotf | 0.31425626 |
| 250 | 0.3 | MAR | RFmfy | 0.33624829 |
| 250 | 0.3 | MAR | RFmf | 0.34330139 |
| 250 | 0.3 | MNAR | KNN | 0.5837997 |
| 250 | 0.3 | MNAR | RFprxt | 0.51163531 |
| 250 | 0.3 | MNAR | RFprxe | 0.49711108 |
| 250 | 0.3 | MNAR | RFotf | 0.59405943 |
| 250 | 0.3 | MNAR | RFmfy | 0.58862952 |
| 250 | 0.3 | MNAR | RFmf | 0.58848186 |
| 500 | 0.1 | MCAR | KNN | 0.34396351 |
| 500 | 0.1 | MCAR | RFprxt | 0.40884804 |
| 500 | 0.1 | MCAR | RFprxe | 0.4838517 |
| 500 | 0.1 | MCAR | RFotf | 0.31143481 |
| 500 | 0.1 | MCAR | RFmfy | 0.32640517 |
| 500 | 0.1 | MCAR | RFmf | 0.3309138 |
| 500 | 0.1 | MAR | KNN | 0.34767592 |
| 500 | 0.1 | MAR | RFprxt | 0.41803998 |
| 500 | 0.1 | MAR | RFprxe | 0.49061519 |
| 500 | 0.1 | MAR | RFotf | 0.30566847 |
| 500 | 0.1 | MAR | RFmfy | 0.32145069 |
| 500 | 0.1 | MAR | RFmf | 0.3248081 |
| 500 | 0.1 | MNAR | KNN | 0.66999443 |
| 500 | 0.1 | MNAR | RFprxt | 0.57881391 |
| 500 | 0.1 | MNAR | RFprxe | 0.50717268 |
| 500 | 0.1 | MNAR | RFotf | 0.70395471 |
| 500 | 0.1 | MNAR | RFmfy | 0.69266163 |
| 500 | 0.1 | MNAR | RFmf | 0.68884932 |
| 500 | 0.2 | MCAR | KNN | 0.33132899 |
| 500 | 0.2 | MCAR | RFprxt | 0.41247675 |
| 500 | 0.2 | MCAR | RFprxe | 0.48275483 |
| 500 | 0.2 | MCAR | RFotf | 0.30690016 |
| 500 | 0.2 | MCAR | RFmfy | 0.32309913 |
| 500 | 0.2 | MCAR | RFmf | 0.32656784 |
| 500 | 0.2 | MAR | KNN | 0.34510503 |
| 500 | 0.2 | MAR | RFprxt | 0.42069138 |
| 500 | 0.2 | MAR | RFprxe | 0.48946092 |
| 500 | 0.2 | MAR | RFotf | 0.30707635 |
| 500 | 0.2 | MAR | RFmfy | 0.32264272 |
| 500 | 0.2 | MAR | RFmf | 0.32842011 |
| 500 | 0.2 | MNAR | KNN | 0.64218305 |
| 500 | 0.2 | MNAR | RFprxt | 0.5501401 |
| 500 | 0.2 | MNAR | RFprxe | 0.50464605 |
| 500 | 0.2 | MNAR | RFotf | 0.65560057 |
| 500 | 0.2 | MNAR | RFmfy | 0.64984474 |
| 500 | 0.2 | MNAR | RFmf | 0.64742498 |
| 500 | 0.3 | MCAR | KNN | 0.32828607 |
| 500 | 0.3 | MCAR | RFprxt | 0.41549084 |
| 500 | 0.3 | MCAR | RFprxe | 0.48083329 |
| 500 | 0.3 | MCAR | RFotf | 0.30734417 |
| 500 | 0.3 | MCAR | RFmfy | 0.3240346 |
| 500 | 0.3 | MCAR | RFmf | 0.32929211 |
| 500 | 0.3 | MAR | KNN | 0.34200546 |
| 500 | 0.3 | MAR | RFprxt | 0.42497643 |
| 500 | 0.3 | MAR | RFprxe | 0.48257927 |
| 500 | 0.3 | MAR | RFotf | 0.30876375 |
| 500 | 0.3 | MAR | RFmfy | 0.32784751 |
| 500 | 0.3 | MAR | RFmf | 0.33161249 |
| 500 | 0.3 | MNAR | KNN | 0.59441869 |
| 500 | 0.3 | MNAR | RFprxt | 0.53055192 |
| 500 | 0.3 | MNAR | RFprxe | 0.50195845 |
| 500 | 0.3 | MNAR | RFotf | 0.59805816 |
| 500 | 0.3 | MNAR | RFmfy | 0.59532801 |
| 500 | 0.3 | MNAR | RFmf | 0.59401624 |
| 1000 | 0.1 | MCAR | KNN | 0.32704203 |
| 1000 | 0.1 | MCAR | RFprxt | 0.39227799 |
| 1000 | 0.1 | MCAR | RFprxe | 0.45609048 |
| 1000 | 0.1 | MCAR | RFotf | 0.30928478 |
| 1000 | 0.1 | MCAR | RFmfy | 0.32023849 |
| 1000 | 0.1 | MCAR | RFmf | 0.32386832 |
| 1000 | 0.1 | MAR | KNN | 0.33807961 |
| 1000 | 0.1 | MAR | RFprxt | 0.3991297 |
| 1000 | 0.1 | MAR | RFprxe | 0.46068453 |
| 1000 | 0.1 | MAR | RFotf | 0.30665034 |
| 1000 | 0.1 | MAR | RFmfy | 0.31671811 |
| 1000 | 0.1 | MAR | RFmf | 0.31940142 |
| 1000 | 0.1 | MNAR | KNN | 0.68699173 |
| 1000 | 0.1 | MNAR | RFprxt | 0.60311684 |
| 1000 | 0.1 | MNAR | RFprxe | 0.52770479 |
| 1000 | 0.1 | MNAR | RFotf | 0.70834048 |
| 1000 | 0.1 | MNAR | RFmfy | 0.70126147 |
| 1000 | 0.1 | MNAR | RFmf | 0.69974076 |
| 1000 | 0.2 | MCAR | KNN | 0.31992044 |
| 1000 | 0.2 | MCAR | RFprxt | 0.39466951 |
| 1000 | 0.2 | MCAR | RFprxe | 0.44604541 |
| 1000 | 0.2 | MCAR | RFotf | 0.30743306 |
| 1000 | 0.2 | MCAR | RFmfy | 0.31786818 |
| 1000 | 0.2 | MCAR | RFmf | 0.32159333 |
| 1000 | 0.2 | MAR | KNN | 0.33967029 |
| 1000 | 0.2 | MAR | RFprxt | 0.40243167 |
| 1000 | 0.2 | MAR | RFprxe | 0.46278779 |
| 1000 | 0.2 | MAR | RFotf | 0.31020278 |
| 1000 | 0.2 | MAR | RFmfy | 0.32085909 |
| 1000 | 0.2 | MAR | RFmf | 0.32504425 |
| 1000 | 0.2 | MNAR | KNN | 0.64694803 |
| 1000 | 0.2 | MNAR | RFprxt | 0.56994787 |
| 1000 | 0.2 | MNAR | RFprxe | 0.52744216 |
| 1000 | 0.2 | MNAR | RFotf | 0.65194742 |
| 1000 | 0.2 | MNAR | RFmfy | 0.64889997 |
| 1000 | 0.2 | MNAR | RFmf | 0.64745685 |
| 1000 | 0.3 | MCAR | KNN | 0.31896566 |
| 1000 | 0.3 | MCAR | RFprxt | 0.39873764 |
| 1000 | 0.3 | MCAR | RFprxe | 0.44690062 |
| 1000 | 0.3 | MCAR | RFotf | 0.30888775 |
| 1000 | 0.3 | MCAR | RFmfy | 0.32028857 |
| 1000 | 0.3 | MCAR | RFmf | 0.32376735 |
| 1000 | 0.3 | MAR | KNN | 0.3351531 |
| 1000 | 0.3 | MAR | RFprxt | 0.4056387 |
| 1000 | 0.3 | MAR | RFprxe | 0.46016945 |
| 1000 | 0.3 | MAR | RFotf | 0.31006913 |
| 1000 | 0.3 | MAR | RFmfy | 0.32218923 |
| 1000 | 0.3 | MAR | RFmf | 0.32604754 |
| 1000 | 0.3 | MNAR | KNN | 0.59836275 |
| 1000 | 0.3 | MNAR | RFprxt | 0.54215094 |
| 1000 | 0.3 | MNAR | RFprxe | 0.5142012 |
| 1000 | 0.3 | MNAR | RFotf | 0.59970969 |
| 1000 | 0.3 | MNAR | RFmfy | 0.59866352 |
| 1000 | 0.3 | MNAR | RFmf | 0.59726165 |

Table S2: Detailed results of the RMSE

| n_sample | missing_rate | amp | method | rmse |
| --- | --- | --- | --- | --- |
| 100 | 0.1 | MCAR | KNN | 0.878158347 |
| 100 | 0.1 | MCAR | RFprxt | 0.953075754 |
| 100 | 0.1 | MCAR | RFprxe | 0.951305681 |
| 100 | 0.1 | MCAR | RFotf | 0.94647202 |
| 100 | 0.1 | MCAR | RFmfy | 0.966958947 |
| 100 | 0.1 | MCAR | RFmf | 0.968407101 |
| 100 | 0.1 | MAR | KNN | 0.874479761 |
| 100 | 0.1 | MAR | RFprxt | 0.967153967 |
| 100 | 0.1 | MAR | RFprxe | 0.960328325 |
| 100 | 0.1 | MAR | RFotf | 0.957143023 |
| 100 | 0.1 | MAR | RFmfy | 0.978425014 |
| 100 | 0.1 | MAR | RFmf | 0.978274161 |
| 100 | 0.1 | MNAR | KNN | 1.058439367 |
| 100 | 0.1 | MNAR | RFprxt | 1.171833431 |
| 100 | 0.1 | MNAR | RFprxe | 1.172098736 |
| 100 | 0.1 | MNAR | RFotf | 1.164073108 |
| 100 | 0.1 | MNAR | RFmfy | 1.175851241 |
| 100 | 0.1 | MNAR | RFmf | 1.178050539 |
| 100 | 0.2 | MCAR | KNN | 0.952217611 |
| 100 | 0.2 | MCAR | RFprxt | 0.98887598 |
| 100 | 0.2 | MCAR | RFprxe | 0.978772524 |
| 100 | 0.2 | MCAR | RFotf | 0.97576484 |
| 100 | 0.2 | MCAR | RFmfy | 0.997967098 |
| 100 | 0.2 | MCAR | RFmf | 1.001043079 |
| 100 | 0.2 | MAR | KNN | 0.951882868 |
| 100 | 0.2 | MAR | RFprxt | 0.993075085 |
| 100 | 0.2 | MAR | RFprxe | 0.982448576 |
| 100 | 0.2 | MAR | RFotf | 0.980859032 |
| 100 | 0.2 | MAR | RFmfy | 1.005008607 |
| 100 | 0.2 | MAR | RFmf | 1.009500248 |
| 100 | 0.2 | MNAR | KNN | 1.110143825 |
| 100 | 0.2 | MNAR | RFprxt | 1.160612009 |
| 100 | 0.2 | MNAR | RFprxe | 1.162171167 |
| 100 | 0.2 | MNAR | RFotf | 1.150673725 |
| 100 | 0.2 | MNAR | RFmfy | 1.165333162 |
| 100 | 0.2 | MNAR | RFmf | 1.167621937 |
| 100 | 0.3 | MCAR | KNN | 0.987098499 |
| 100 | 0.3 | MCAR | RFprxt | 1.010953287 |
| 100 | 0.3 | MCAR | RFprxe | 0.998530913 |
| 100 | 0.3 | MCAR | RFotf | 0.992894144 |
| 100 | 0.3 | MCAR | RFmfy | 1.014620571 |
| 100 | 0.3 | MCAR | RFmf | 1.017997281 |
| 100 | 0.3 | MAR | KNN | 0.994196092 |
| 100 | 0.3 | MAR | RFprxt | 1.018303981 |
| 100 | 0.3 | MAR | RFprxe | 1.007053247 |
| 100 | 0.3 | MAR | RFotf | 1.003290583 |
| 100 | 0.3 | MAR | RFmfy | 1.026258154 |
| 100 | 0.3 | MAR | RFmf | 1.031944042 |
| 100 | 0.3 | MNAR | KNN | 1.09734942 |
| 100 | 0.3 | MNAR | RFprxt | 1.134810276 |
| 100 | 0.3 | MNAR | RFprxe | 1.127852737 |
| 100 | 0.3 | MNAR | RFotf | 1.119349801 |
| 100 | 0.3 | MNAR | RFmfy | 1.136623362 |
| 100 | 0.3 | MNAR | RFmf | 1.143087665 |
| 250 | 0.1 | MCAR | KNN | 0.960266662 |
| 250 | 0.1 | MCAR | RFprxt | 0.997704131 |
| 250 | 0.1 | MCAR | RFprxe | 0.992346001 |
| 250 | 0.1 | MCAR | RFotf | 0.989654813 |
| 250 | 0.1 | MCAR | RFmfy | 1.009191126 |
| 250 | 0.1 | MCAR | RFmf | 1.012964279 |
| 250 | 0.1 | MAR | KNN | 0.96694266 |
| 250 | 0.1 | MAR | RFprxt | 1.004642524 |
| 250 | 0.1 | MAR | RFprxe | 0.998032746 |
| 250 | 0.1 | MAR | RFotf | 0.995463409 |
| 250 | 0.1 | MAR | RFmfy | 1.014247701 |
| 250 | 0.1 | MAR | RFmf | 1.019903052 |
| 250 | 0.1 | MNAR | KNN | 1.175060139 |
| 250 | 0.1 | MNAR | RFprxt | 1.224808004 |
| 250 | 0.1 | MNAR | RFprxe | 1.2199194 |
| 250 | 0.1 | MNAR | RFotf | 1.217943709 |
| 250 | 0.1 | MNAR | RFmfy | 1.230307485 |
| 250 | 0.1 | MNAR | RFmf | 1.233090064 |
| 250 | 0.2 | MCAR | KNN | 0.997658805 |
| 250 | 0.2 | MCAR | RFprxt | 1.016671412 |
| 250 | 0.2 | MCAR | RFprxe | 1.004840703 |
| 250 | 0.2 | MCAR | RFotf | 1.001812172 |
| 250 | 0.2 | MCAR | RFmfy | 1.021674273 |
| 250 | 0.2 | MCAR | RFmf | 1.025783895 |
| 250 | 0.2 | MAR | KNN | 0.993766326 |
| 250 | 0.2 | MAR | RFprxt | 1.011934531 |
| 250 | 0.2 | MAR | RFprxe | 1.001676208 |
| 250 | 0.2 | MAR | RFotf | 0.997759328 |
| 250 | 0.2 | MAR | RFmfy | 1.017102468 |
| 250 | 0.2 | MAR | RFmf | 1.018731771 |
| 250 | 0.2 | MNAR | KNN | 1.148899482 |
| 250 | 0.2 | MNAR | RFprxt | 1.176901843 |
| 250 | 0.2 | MNAR | RFprxe | 1.170958064 |
| 250 | 0.2 | MNAR | RFotf | 1.165284241 |
| 250 | 0.2 | MNAR | RFmfy | 1.18133829 |
| 250 | 0.2 | MNAR | RFmf | 1.184499947 |
| 250 | 0.3 | MCAR | KNN | 1.008325784 |
| 250 | 0.3 | MCAR | RFprxt | 1.020636165 |
| 250 | 0.3 | MCAR | RFprxe | 1.004791364 |
| 250 | 0.3 | MCAR | RFotf | 1.004150461 |
| 250 | 0.3 | MCAR | RFmfy | 1.020880458 |
| 250 | 0.3 | MCAR | RFmf | 1.026399872 |
| 250 | 0.3 | MAR | KNN | 1.009856597 |
| 250 | 0.3 | MAR | RFprxt | 1.025369055 |
| 250 | 0.3 | MAR | RFprxe | 1.008376254 |
| 250 | 0.3 | MAR | RFotf | 1.0070389 |
| 250 | 0.3 | MAR | RFmfy | 1.025244187 |
| 250 | 0.3 | MAR | RFmf | 1.030128685 |
| 250 | 0.3 | MNAR | KNN | 1.127842189 |
| 250 | 0.3 | MNAR | RFprxt | 1.144079726 |
| 250 | 0.3 | MNAR | RFprxe | 1.138175215 |
| 250 | 0.3 | MNAR | RFotf | 1.131159214 |
| 250 | 0.3 | MNAR | RFmfy | 1.145009043 |
| 250 | 0.3 | MNAR | RFmf | 1.149369299 |
| 500 | 0.1 | MCAR | KNN | 0.980080631 |
| 500 | 0.1 | MCAR | RFprxt | 1.001363943 |
| 500 | 0.1 | MCAR | RFprxe | 0.992025509 |
| 500 | 0.1 | MCAR | RFotf | 0.992391926 |
| 500 | 0.1 | MCAR | RFmfy | 1.008214361 |
| 500 | 0.1 | MCAR | RFmf | 1.011969434 |
| 500 | 0.1 | MAR | KNN | 0.985158948 |
| 500 | 0.1 | MAR | RFprxt | 1.008526383 |
| 500 | 0.1 | MAR | RFprxe | 0.998159068 |
| 500 | 0.1 | MAR | RFotf | 0.997311708 |
| 500 | 0.1 | MAR | RFmfy | 1.014123544 |
| 500 | 0.1 | MAR | RFmf | 1.018729521 |
| 500 | 0.1 | MNAR | KNN | 1.209162169 |
| 500 | 0.1 | MNAR | RFprxt | 1.238230343 |
| 500 | 0.1 | MNAR | RFprxe | 1.238676615 |
| 500 | 0.1 | MNAR | RFotf | 1.230740427 |
| 500 | 0.1 | MNAR | RFmfy | 1.24336986 |
| 500 | 0.1 | MNAR | RFmf | 1.245888729 |
| 500 | 0.2 | MCAR | KNN | 1.004518092 |
| 500 | 0.2 | MCAR | RFprxt | 1.017556495 |
| 500 | 0.2 | MCAR | RFprxe | 1.002746846 |
| 500 | 0.2 | MCAR | RFotf | 1.002819774 |
| 500 | 0.2 | MCAR | RFmfy | 1.019586121 |
| 500 | 0.2 | MCAR | RFmf | 1.022933923 |
| 500 | 0.2 | MAR | KNN | 1.004391318 |
| 500 | 0.2 | MAR | RFprxt | 1.020981205 |
| 500 | 0.2 | MAR | RFprxe | 1.006162193 |
| 500 | 0.2 | MAR | RFotf | 1.006714819 |
| 500 | 0.2 | MAR | RFmfy | 1.02149189 |
| 500 | 0.2 | MAR | RFmf | 1.025799384 |
| 500 | 0.2 | MNAR | KNN | 1.17350925 |
| 500 | 0.2 | MNAR | RFprxt | 1.191043567 |
| 500 | 0.2 | MNAR | RFprxe | 1.191468436 |
| 500 | 0.2 | MNAR | RFotf | 1.180537915 |
| 500 | 0.2 | MNAR | RFmfy | 1.192562269 |
| 500 | 0.2 | MNAR | RFmf | 1.195130673 |
| 500 | 0.3 | MCAR | KNN | 1.009957305 |
| 500 | 0.3 | MCAR | RFprxt | 1.023051728 |
| 500 | 0.3 | MCAR | RFprxe | 1.005486231 |
| 500 | 0.3 | MCAR | RFotf | 1.0049592 |
| 500 | 0.3 | MCAR | RFmfy | 1.019486813 |
| 500 | 0.3 | MCAR | RFmf | 1.023223592 |
| 500 | 0.3 | MAR | KNN | 1.009839169 |
| 500 | 0.3 | MAR | RFprxt | 1.025464434 |
| 500 | 0.3 | MAR | RFprxe | 1.006606476 |
| 500 | 0.3 | MAR | RFotf | 1.006982205 |
| 500 | 0.3 | MAR | RFmfy | 1.021949752 |
| 500 | 0.3 | MAR | RFmf | 1.026261774 |
| 500 | 0.3 | MNAR | KNN | 1.13668086 |
| 500 | 0.3 | MNAR | RFprxt | 1.153041286 |
| 500 | 0.3 | MNAR | RFprxe | 1.15111241 |
| 500 | 0.3 | MNAR | RFotf | 1.137969743 |
| 500 | 0.3 | MNAR | RFmfy | 1.149876358 |
| 500 | 0.3 | MNAR | RFmf | 1.153201557 |
| 1000 | 0.1 | MCAR | KNN | 0.995789747 |
| 1000 | 0.1 | MCAR | RFprxt | 1.009229435 |
| 1000 | 0.1 | MCAR | RFprxe | 0.999370376 |
| 1000 | 0.1 | MCAR | RFotf | 0.999557067 |
| 1000 | 0.1 | MCAR | RFmfy | 1.012461302 |
| 1000 | 0.1 | MCAR | RFmf | 1.016702563 |
| 1000 | 0.1 | MAR | KNN | 0.996323778 |
| 1000 | 0.1 | MAR | RFprxt | 1.011213647 |
| 1000 | 0.1 | MAR | RFprxe | 1.001618692 |
| 1000 | 0.1 | MAR | RFotf | 1.001864381 |
| 1000 | 0.1 | MAR | RFmfy | 1.01404236 |
| 1000 | 0.1 | MAR | RFmf | 1.017220897 |
| 1000 | 0.1 | MNAR | KNN | 1.220765584 |
| 1000 | 0.1 | MNAR | RFprxt | 1.238853436 |
| 1000 | 0.1 | MNAR | RFprxe | 1.245902158 |
| 1000 | 0.1 | MNAR | RFotf | 1.232359249 |
| 1000 | 0.1 | MNAR | RFmfy | 1.240916533 |
| 1000 | 0.1 | MNAR | RFmf | 1.244084636 |
| 1000 | 0.2 | MCAR | KNN | 1.003364554 |
| 1000 | 0.2 | MCAR | RFprxt | 1.016428122 |
| 1000 | 0.2 | MCAR | RFprxe | 1.003698198 |
| 1000 | 0.2 | MCAR | RFotf | 1.002516389 |
| 1000 | 0.2 | MCAR | RFmfy | 1.013717638 |
| 1000 | 0.2 | MCAR | RFmf | 1.017887739 |
| 1000 | 0.2 | MAR | KNN | 1.005551366 |
| 1000 | 0.2 | MAR | RFprxt | 1.018460523 |
| 1000 | 0.2 | MAR | RFprxe | 1.005466424 |
| 1000 | 0.2 | MAR | RFotf | 1.004990899 |
| 1000 | 0.2 | MAR | RFmfy | 1.016516294 |
| 1000 | 0.2 | MAR | RFmf | 1.020220401 |
| 1000 | 0.2 | MNAR | KNN | 1.176583391 |
| 1000 | 0.2 | MNAR | RFprxt | 1.191346815 |
| 1000 | 0.2 | MNAR | RFprxe | 1.193075584 |
| 1000 | 0.2 | MNAR | RFotf | 1.179963985 |
| 1000 | 0.2 | MNAR | RFmfy | 1.187512668 |
| 1000 | 0.2 | MNAR | RFmf | 1.190486427 |
| 1000 | 0.3 | MCAR | KNN | 1.008111339 |
| 1000 | 0.3 | MCAR | RFprxt | 1.024905943 |
| 1000 | 0.3 | MCAR | RFprxe | 1.009373817 |
| 1000 | 0.3 | MCAR | RFotf | 1.00603146 |
| 1000 | 0.3 | MCAR | RFmfy | 1.016699792 |
| 1000 | 0.3 | MCAR | RFmf | 1.020206286 |
| 1000 | 0.3 | MAR | KNN | 1.010192424 |
| 1000 | 0.3 | MAR | RFprxt | 1.027679877 |
| 1000 | 0.3 | MAR | RFprxe | 1.011600735 |
| 1000 | 0.3 | MAR | RFotf | 1.00878145 |
| 1000 | 0.3 | MAR | RFmfy | 1.018526979 |
| 1000 | 0.3 | MAR | RFmf | 1.021976557 |
| 1000 | 0.3 | MNAR | KNN | 1.138536716 |
| 1000 | 0.3 | MNAR | RFprxt | 1.156068045 |
| 1000 | 0.3 | MNAR | RFprxe | 1.15297704 |
| 1000 | 0.3 | MNAR | RFotf | 1.138813665 |
| 1000 | 0.3 | MNAR | RFmfy | 1.147335313 |
| 1000 | 0.3 | MNAR | RFmf | 1.150288831 |

Table S3: The run time with sample size 100 (seconds)


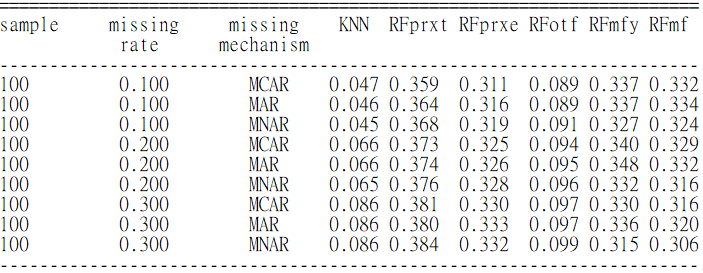


The run time with sample size 250 (seconds)


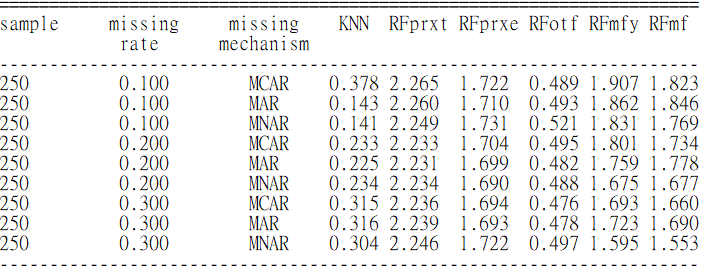


The run time with sample size 500 (seconds)


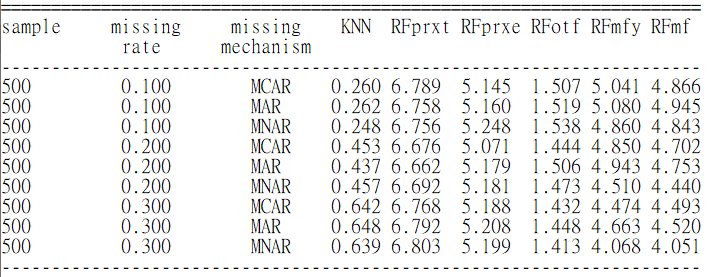


The run time with sample size 1000 (seconds)


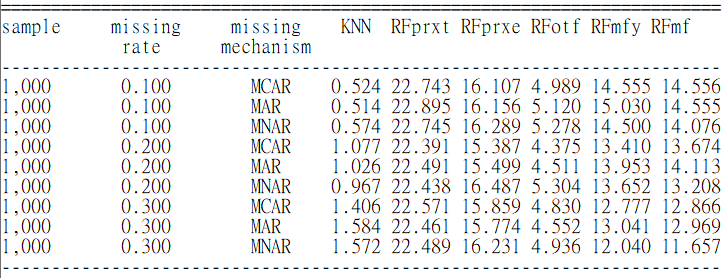

Supplement: Supplementary file 1 [file Data_Sheet_1.docx]
