# Supplementary material for: The Optimal Machine Learning-Based Missing Data Imputation for the Cox Proportional Hazard Model
Source: Front Public Health. 2021 Jul 5;9:680054. doi: 10.3389/fpubh.2021.680054 (PMC8289437; doi:10.3389/fpubh.2021.680054)
Supplement: Supplementary file 2 [file Data_Sheet_2.docx]

### STEP 1

### USER_UTILITY CODE IN R ENVIRONMENT

### The Optimal Machine Learning-Based Missing Data Imputation for

### the Cox Proportional Hazard Model

### Chao-Yu Guo^1,*^, Ying-Chen Yang^1^, Yi-Hau Chen^2^

### 1Institute of Public Health, National Yang-Ming University

### 2Institute of Statistical Science, Academia Sinica

### *Address Correspondence

## R Core Team (2017). R: A language and environment for

## statistical computing. R Foundation for Statistical Computing,

## Vienna, Austria. URL [https://www.R-project.org/](https://www.r-project.org/).

library(mice)

library(simstudy)

library(magrittr)

library(dplyr)

library(coxed)

library(DMwR)

library(caret)

library(Hmisc)

library(randomForest)

library(randomForestSRC)

library(missForest)

library(VIM)

library(laeken)

library(simsurv)

# ====================================================================================

# Define useful functions

# ====================================================================================

RMSE <- function(m, o) {

sqrt(mean((m - o)^2))

}

RMSE_dataframe <- function(m, o) {

data.substracted <- unlist(m - o, use.names = FALSE)

data.substracted %>%

extract(data.substracted != 0) %>%

`^`(2) %>%

mean() %>%

sqrt()

}

NRMSE_dataframe <- function(m, o) {

data.substracted <- unlist(m - o, use.names = FALSE)

o.sd <- o %>%

unlist(use.names = FALSE) %>%

extract(data.substracted != 0) %>%

sd()

data.substracted %>%

extract(data.substracted != 0) %>%

raise_to_power(2) %>%

mean() %>%

sqrt() %>%

divide_by(o.sd)

}

function_complete_binary <- function(value) {

if (value > 0.5) 1 else 0

}

PFC_variable <- function(predict, actual, na.rows) {

mean(predict[na.rows] != actual[na.rows])

}

RMSE_variable <- function(predict, actual, na.rows) {

(predict[na.rows] - actual[na.rows]) %>%

`^`(2) %>%

mean() %>%

sqrt()

}

PFC_result <- function(ori, amp, imp) {

indicator <- is.na(amp)

real <- ori[indicator == TRUE]

pre <- imp[indicator == TRUE]

sum(real != pre) / length(real)

}

RMSE_result <- function(ori, amp, imp) {

indicator <- is.na(amp)

real <- ori[indicator == TRUE]

pre <- imp[indicator == TRUE]

sqrt(mean((real - pre)^2))

}

VIMKNN_result <- function(ori_df, amp_df, k, n_binary, n_continuous) {

library(VIM)

data.vimknn <- NULL

data.vimknn$imputed <- kNN(

k = k,

amp_df,

variable = colnames(amp_df)[1:(n_binary + n_continuous)],

numFun = weightedMean,

weightDist = TRUE

)

# ori, amp, imp

data.vimknn$pfc <- PFC_result(

ori_df[, 1:n_binary],

amp_df[, 1:n_binary],

data.vimknn$imputed[, 1:n_binary]

)

# cat("\nPFC: ", data.vimknn$pfc)

data.vimknn$rmse <- RMSE_result(

ori_df[, (1 + n_binary):(n_binary + n_continuous)],

amp_df[, (1 + n_binary):(n_binary + n_continuous)],

data.vimknn$imputed[, (1 + n_binary):(n_binary + n_continuous)]

)

# cat("\nRMSE: ", data.vimknn$rmse)

return(data.vimknn)

}

get_data_pfc_rmse <- function(ori_df, amp_df, imp_df, n_binary, n_continuous) {

data <- NULL

data$imputed <- imp_df

# ori, amp, imp

data$pfc <- PFC_result(

ori_df[, 1:n_binary],

amp_df[, 1:n_binary],

imp_df[, 1:n_binary]

)

# cat("\nPFC: ", data.vimknn$pfc)

data$rmse <- RMSE_result(

ori_df[, (1 + n_binary):(n_binary + n_continuous)],

amp_df[, (1 + n_binary):(n_binary + n_continuous)],

imp_df[, (1 + n_binary):(n_binary + n_continuous)]

)

# cat("\nRMSE: ", dat

return(data)

}

gen_missing_pattern_weights <- function(simdata, n_binary, n_continuous) {

missing.pattern_weights <- NULL

missing.pattern <- NULL

missing.pattern$binary <- rep(1, n_binary)

missing.pattern$continuous <- rep(1, n_continuous)

missing.pattern$y <- rep(1, 2)

missing.pattern$binary.function <- function(values, index) {

if (index == simdata$missing.binary) 0 else 1

}

missing.pattern$continuous.function <- function(values, index) {

if (index == simdata$missing.continuous) 0 else 1

}

missing.pattern$pattern <- as.data.frame(rbind(

c(

mapply(missing.pattern$binary.function, missing.pattern$binary, seq_along(missing.pattern$binary)),

mapply(missing.pattern$continuous.function, missing.pattern$continuous, seq_along(missing.pattern$continuous)),

missing.pattern$y

),

c(

missing.pattern$binary,

mapply(missing.pattern$continuous.function, missing.pattern$continuous, seq_along(missing.pattern$continuous)),

missing.pattern$y

),

c(

mapply(missing.pattern$binary.function, missing.pattern$binary, seq_along(missing.pattern$binary)),

missing.pattern$continuous,

missing.pattern$y

)

), stringsAsFactors = FALSE)

names(missing.pattern$pattern) <- colnames(simdata$data)

# missing.pattern <- as.data.frame(rbind(

# c(0, 1, 1, 1, 0, 1, 1, 1, 1, 1, 1, 1),

# c(1, 1, 1, 1, 0, 1, 1, 1, 1, 1, 1, 1),

# c(0, 1, 1, 1, 1, 1, 1, 1, 1, 1, 1, 1)

# ), stringsAsFactors = FALSE)

# names(missing.pattern) <- colnames(df)

missing.weights <- NULL

missing.weights$y <- rep(0, 2)

missing.weights$weights <- as.data.frame(rbind(

c(

mapply(missing.pattern$binary.function, missing.pattern$binary, seq_along(missing.pattern$binary)),

mapply(missing.pattern$continuous.function, missing.pattern$continuous, seq_along(missing.pattern$continuous)),

missing.weights$y

),

c(

missing.pattern$binary,

mapply(missing.pattern$continuous.function, missing.pattern$continuous, seq_along(missing.pattern$continuous)),

missing.weights$y

),

c(

mapply(missing.pattern$binary.function, missing.pattern$binary, seq_along(missing.pattern$binary)),

missing.pattern$continuous,

missing.weights$y

)

), stringsAsFactors = FALSE)

names(missing.weights$weights) <- colnames(simdata$data)

# missing.weights <- as.data.frame(rbind(

# c(0, 1, 1, 1, 0, 1, 1, 1, 1, 1, 0, 0),

# c(1, 1, 1, 1, 0, 1, 1, 1, 1, 1, 0, 0),

# c(0, 1, 1, 1, 1, 1, 1, 1, 1, 1, 0, 0)

# ), stringsAsFactors = FALSE)

# names(missing.weights) <- colnames(df)

missing.pattern_weights$pattern <- missing.pattern$pattern

missing.pattern_weights$weights <- missing.weights$weights

return(missing.pattern_weights)

}

gen_amputation_data <- function(simdata, missing_freq, missing_rate, n_binary, n_continous) {

simdata.amputaion <- NULL

missing <- gen_missing_pattern_weights(simdata, n_binary, n_continous)

simdata.missing$MCAR <- ampute(simdata$data, mech = "MCAR", pattern = missing$pattern, prop = missing_rate, freq = missing_freq)

simdata.missing$MAR <- ampute(simdata$data, mech = "MAR", pattern = missing$pattern, prop = missing_rate, freq = missing_freq, weights = missing$weights)

simdata.missing$MNAR <- ampute(simdata$data, mech = "MNAR", pattern = missing$pattern, prop = missing_rate, freq = missing_freq)

return(simdata.missing)

}

getmode <- function(v) {

uniqv <- unique(v)

uniqv[which.max(tabulate(match(v, uniqv)))]

}

# generate.survdata <- function(nData, maxTime, nBinary, nContinuous) {

# counter <- 1

# def <- NULL

# if (nBinary > 0) {

# for (i in c(1:nBinary)) {

# def <- defData(def, varname = paste("x", counter, sep = ""), formula = 0.5, dist = "binary", link = "logic")

# counter <- counter + 1

# }

# }

# if (nContinuous > 0) {

# for (i in c(1:nContinuous)) {

# def <- defData(def, varname = paste("x", counter, sep = ""), formula = 0, variance = 1, dist = "normal")

# counter <- counter + 1

# }

# }

# defData <- genData(nData, def) %>%

# select(-1)

# survData <- sim.survdata(N = nData, T = maxTime, X = defData, num.data.frames = 1)

# survData$data$failed <- survData$data$failed %>%

# as.logical() %>%

# as.integer()

# return(survData)

# }

generate.survdata <- function(nData, maxTime, nBinary, nContinuous) {

counter <- 1

def <- NULL

nVariable <- nBinary + nContinuous

for (i in c(1:nVariable)) {

def <- defData(def, varname = paste("x", counter, sep = ""), formula = 0, variance = 1, dist = "normal")

counter <- counter + 1

}

# Generate X 0310

defData <- genData(nData, def) %>%

as.data.frame() %>%

select(-1)

# Changed 0324

uniData <- NULL

test_idx <- 1

for (i in c("x1", "x2", "x3")) {

uniData[[i]] <- runif(nData, min = 0, max = 1)

}

uniData = data.frame(uniData)

for (i in c(1:3)) {

uniData[, i] <- mapply(function_complete_binary, uniData[, i])

}

# Use the uniData to generate y

survStatus <- simsurv(lambdas = 0.5, dist = "exponential", maxt = maxTime, x = uniData, tdefunction = "log") %>% select(-1)

# survStatus <- sim.survdata(N = nData, T = maxTime, X = uniData, num.data.frames = 1)

# survStatus <- survStatus$data[c(11, 12)]

colnames(survStatus) <- c("time", "event")

survData <- NULL

# Merge the y w/ the generated y

survData$data <- merge(defData, survStatus, by = "row.names", all = TRUE) %>%

arrange(as.integer(Row.names)) %>%

select(-1)

# Use R squared to compare correlation

survData$r_squared <- survData$data %>%

select(1:(nBinary + nContinuous)) %>%

cor() %>%

`^`(2)

# Find 10 largest correlation

find_cor <- survData$r_squared

max.cor <- NULL

for (i in c(1:(nBinary + nContinuous))) {

find_cor[i, i] <- 0

max.cor[i] <- 0

}

for (i in c(1:10)) {

max.cor.idx <- which(find_cor == max(find_cor), arr.ind = TRUE)

max.cor[max.cor.idx[1]] <- max.cor[max.cor.idx[1]] + 1

max.cor[max.cor.idx[2]] <- max.cor[max.cor.idx[2]] + 1

find_cor[max.cor.idx[1], max.cor.idx[2]] <- 0

find_cor[max.cor.idx[3], max.cor.idx[4]] <- 0

}

cat("\nTop 10 correlation appear time: ", max.cor)

survData$missing.binary <- max.cor[1:nBinary] %>% which.max()

survData$na.rows.binary <- is.na(survData$data[, survData$missing.binary])

survData$missing.continuous <- max.cor[(nBinary + 1):(nBinary + nContinuous)] %>% which.max()

survData$na.rows.continuous <- is.na(survData$data[, survData$missing.continuous])

# Convert to binary

if (nBinary > 0) {

for (i in c(1:nBinary)) {

survData$data[, i] <- mapply(function_complete_binary, survData$data[, i])

}

}

return(survData)

}

impute_by_mean_and_mode <- function(missing.data, missing.binary, missing.continuous) {

data.imputed <- missing.data

# Impute binary by mode

na.rows.binary <- is.na(missing.data[, missing.binary])

data.imputed[

na.rows.binary,

missing.binary

] <-

getmode(missing.data[!na.rows.binary, missing.binary])

# Impute continuous by mean

na.rows.continuous <- is.na(missing.data[, missing.continuous])

data.imputed[

na.rows.continuous,

missing.continuous

] <-

mean(missing.data[!na.rows.continuous, missing.continuous], na.rm = TRUE)

return(data.imputed)

}

generate.missing <- function(df, missingRate, missingPattern, missingMech, missingWeights = NULL, missingFreq = NULL) {

names(missingPattern) <- colnames(df)

if (length(missingWeights) == 0) {

dtMissing <- ampute(df, pattern = missingPattern, prop = missingRate, mech = missingMech)

} else {

names(missingWeights) <- colnames(df)

dtMissing <- ampute(df, pattern = missingPattern, prop = missingRate, mech = missingMech, weights = missingWeights)

}

return(dtMissing)

}

crossvalidate_knnimputation <- function(missing.data, first.inputed.data, full.data) {

set.seed(123)

training.samples <- missing.data$y %>%

createDataPartition(p = 0.7, list = FALSE)

train.data <- missing.data[training.samples, ]

test.data <- missing.data[-training.samples, ]

validate.data <- first.inputed.data[-training.samples, ]

rownames(train.data) <- NULL

rownames(test.data) <- NULL

test.imputed <- NULL

imputed.rmse <- NULL

for (i in 1:floor(sqrt(nrow(train.data)))) {

# https://www.rdocumentation.org/packages/DMwR/versions/0.4.1/topics/knnImputation

test.imputed <- knnImputation(

test.data,

distData = train.data,

scale = T, meth = "weighAvg", k = i

)

imputed.rmse[i] <- RMSE_dataframe(validate.data, test.imputed)

}

min.k <- which.min(imputed.rmse)

cat("Minimal k value:", min.k)

imputed.data <- knnImputation(missing.data, scale = T, meth = "weighAvg", k = min.k)

rmse <- RMSE_dataframe(full.data, imputed.data)

return(list(

"rmse" = rmse,

"k" = min.k,

"data" = imputed.data,

"training.rmse" = imputed.rmse,

"training.k" = 1:floor(sqrt(nrow(train.data)))

))

}

crossvalidate_randomforest <- function(missing.data, first.inputed.data, full.data) {

imputed.rmse <- NULL

imputed.nrmse <- NULL

sample_leaf_options <- c(1, 5, 10, 50, 100, 200, 500)

for (i in 1:length(sample_leaf_options)) {

# https://www.rdocumentation.org/packages/missForest/versions/1.4/topics/missForest

data.imputed <- missForest(

missing.data,

xtrue = first.inputed.data,

nodesize = c(sample_leaf_options[i], 5)

)

imputed.rmse[i] <- RMSE_dataframe(first.inputed.data, data.imputed$ximp)

imputed.nrmse[i] <- data.imputed$error %>%

as.numeric()

}

# Find mininum RMSE when nodesize = i

min.leaf <- which.min(imputed.rmse)

imputed.data <- missForest(

missing.data,

xtrue = first.inputed.data,

nodesize = c(sample_leaf_options[min.leaf], 5)

)

error <- imputed.data$error

ooberror <- imputed.data$OOBerror

cat("Minimal leaf value:", sample_leaf_options[min.leaf])

rmse <- RMSE_dataframe(full.data, imputed.data$ximp)

return(list(

"error" = error,

"ooberror" = ooberror,

"rmse" = rmse,

"leaf" = sample_leaf_options[min.leaf],

"data" = imputed.data$ximp,

"training.rmse" = imputed.rmse,

"training.nodesize" = sample_leaf_options

))

}
